# Evolution of tonal organization in music mirrors symbolic representation of perceptual reality. Part-1: Prehistoric

**DOI:** 10.3389/fpsyg.2015.01405

**Published:** 2015-10-16

**Authors:** Aleksey Nikolsky

**Affiliations:** Braavo EnterprisesLos Angeles, CA, USA

**Keywords:** music perception, evolution of music, musical and verbal intonation, tonality, musical mode, horizontal/vertical harmony, diatonic/chromatic music, pentatonic/heptatonic music

## Abstract

This paper reveals the way in which musical pitch works as a peculiar form of cognition that reflects upon the organization of the surrounding world as perceived by majority of music users within a socio-cultural formation. The evidence from music theory, ethnography, archeology, organology, anthropology, psychoacoustics, and evolutionary biology is plotted against experimental evidence. Much of the methodology for this investigation comes from studies conducted within the territory of the former USSR. To date, this methodology has remained solely confined to Russian speaking scholars. A brief overview of pitch-set theory demonstrates the need to distinguish between vertical and horizontal harmony, laying out the framework for virtual music space that operates according to the perceptual laws of tonal gravity. Brought to life by bifurcation of music and speech, tonal gravity passed through eleven discrete stages of development until the onset of tonality in the seventeenth century. Each stage presents its own method of integration of separate musical tones into an auditory-cognitive unity. The theory of “melodic intonation” is set forth as a counterpart to harmonic theory of chords. Notions of tonality, modality, key, diatonicity, chromaticism, alteration, and modulation are defined in terms of their perception, and categorized according to the way in which they have developed historically. Tonal organization in music, and perspective organization in fine arts are explained as products of the same underlying mental process. Music seems to act as a unique medium of symbolic representation of reality through the concept of pitch. Tonal organization of pitch reflects the culture of thinking, adopted as a standard within a community of music users. Tonal organization might be a naturally formed system of optimizing individual perception of reality within a social group and its immediate environment, setting conventional standards of intellectual and emotional intelligence.

The phenomenon of tonal organization in music has attracted attention of scholars from numerous fields: music theory, history, ethnomusicology and, more recently, cognitive psychology. Each of these disciplines has elaborated its own framework of study, with its own taxonomy and terminology, making it hard to cross-relate findings from different areas of research. To add to the confusion, there is little correlation between theories that originated in countries of the former Soviet block and Western research. This paper attempts to bring the vast data to a common denominator, based on the framework of cognitive psychology, and identify the principal models of tonal organization in the course of its evolution—from its origin to the rise of Western tonality.

Szabolcsi (1965) came closest to drafting this evolutionary outlook up until the twentieth century, however, he barely touched upon the earliest forms (crucial for separation of music from speech), and limited his research to pentatonic, heptatonic, and chromatic systems—primarily from musicological perspective, based on melodic analysis. Significant gains of archeological (Morley, 2013) and ethnomusicological (Sheikin, 2002) research in the past half-a-century, as well as technological progress in sound analysis tools (Schneider, 2013), allow to draw a much finer picture of typology of early music and relate existing musical traditions to known prehistoric cultures. This paper identifies 12 known stages of tonal development—pentatony being chronologically the 7th in this order.

Emergence of biomusicology (Wallin, 1991) triggered interest in matters of origin of music (Wallin and Merker, 2001), bringing in disciplines of evolutionary biology and neurophysiology (Altenmuller et al., 2015). Such input allowed to reduce controversy that balked the development of evolutionary theory in Western ethnomusicology: when scholars refused to accept the idea of a single “world music” passing through evolutionary changes—instead, envisaging multiple “musics,” each passing its own course of development (Blacking, 1974). In such view, any imposition of cross-cultural categorization would misrepresent native music theories (Nettl, 2005, p. 112). Biological sciences have answered this objection by identifying features of musical perception shared by all humans. These features can establish the foundation for cross-cultural investigation of tonal organization. As such, typology of tonal perception can be linked to typology of tonal composition—materializing Riemann's “relational thinking” that governs listener's ability to realize coherence of melodic contours and intervals (Neuhaus, 2013); and Handschin's (1995) “tone-character” that enables listener to distinguish one pitch from another.

People hear certain tones as matching each other by processing frequency in a particular way. These ways are finite in number, cultivated within a particular social group and are determined by the interaction of individuals within this group and with their environment. One of such methods—Western tonality—has been successfully investigated by cognitive science (Krumhansl, 1990). The methodology of its research can be adapted to study other methods of unification of musical sounds into a perceptual sonic ensemble. This paper drafts the foundation for such research and is broken into two parts: prehistoric and historic. The historic is based on the examination of documented music theory (Christensen, 2008) and organology (Dumbrill, 2005), correlated with analysis of music samples, wherever possible. The prehistoric is based primarily on generalizations from comparative morphological analysis of multiple music samples by experts in a given folk culture. Here, despite speculation and risk of misrepresenting the native music theory, reliance on experts' interpretation is inevitable. Comparative scientific study of different cultures is only possible when data is presented in terms of a coherent comprehensive music theory, and processed by uniformed analytical procedures (Schneider, 2006).

Rational analysis and speculation have been the instruments of scientific investigation of music—kept in check by empirical examination of conclusions (Schneider, 2010). Ideally, the inferred tonal principles should be tested on native listeners to see whether or not they authenticate production of music according to hypothesized rules (Arom, 2010). The established models of tonal unification can then be cross-examined to see if one is derived from another—decided by geographic (Zemcovskij, 2005) and ethnic distribution of certain musical features (Grauer, 2006) vs. estimation of mental processing involved in perception of that music^1^. Finally, the discovered type of mental operation can be related to other cultural activities that have been dated by archeologists^2^—thus, I compare tonal organization with spatial representation in art works. Appendix II (Supplementary Material) offers a novel method of inferring tonal organization from musical instruments based on the methodology by Beliayev (1990). Applied to archeological finds, this allows drafting approximate timeline of the introduction of each of the tonal models. Similarity between social organization in a modern ethnic community and the one revealed by archeological research of the past suggests similarity between their music systems (Both, 2009). Tonal evolution can be as helpful for anthropology as the study of technological modes of manufacturing stone tools (Foley and Lahr, 2003).

Biological and physiological constraints, together with laws of psychoacoustics, determine commonality in music production across different synchronic and diachronic cultures. In this broadest sense, “music” can be defined as *the arrangement of sounds in relation to their amplitude, frequency, duration, and spectral content, which entrains groups of people, and is used to transpose intentions in order to emotionally stir the listener in a certain way by means of vocal and/or instrumental performance*. Such definition encapsulates pitchless timbre-driven vocalizations that are still encountered in Siberia embedded in pitched music (Ojamaa, 2005), and allows culture-historic comparison of different “musics.”

The proposed stages of tonal organization should not be viewed as phenomenological laws, but as cognitive constructs similar to Piagetian stages of mental development, where each stage represents a particular style of integration of cultural data (Goodman, 1976, p. 11). The idea of applying Piaget's framework onto the evolution of human intelligence was introduced by Wynn (1985) and accepted by many anthropologists as useful means of interpretation, albeit without consensus regarding how exactly the prehistoric cultural periods correspond to Piaget's stages. The progression of “associational,” “logical,” and “hypothetical” stages in culture of thinking (Parker and Jaffe, 2008, p. 188) roughly matches three general “ages” of music:

- indefinite pitch organization that supports timbre and articulation;
- elementary definite pitch organization limited to small sets;
- hierarchical organization that requires parallel top-to-bottom/bottom-to-top operation, exercised through frequent categorization assumptions and their confirmation/negation.

Tonal models appear to be cumulative—music representative of each of them can be encountered within the same culture (Alekseyev, 1986).

Arranged according to their lineage, stages of tonal organization provide unique outlook on development of human consciousness, and establish a frame of reference for understanding the role of music and language as biological markers of Homo sapiens. Opposition of language, as bearer of cognitive dissonance, to music (Perlovsky, 2014) which then accepts the function of “cognitive consonance,” leads one to believe that “cognitive consonance” is that elusive adaptive value of music which has been sought after since Darwin and Spencer (Honing and Ploeger, 2012). Music's “consonant” function is evident in the mode of its default perception: we tend to integrate concurrent musical sounds, but segregate sounds of speech (Bregman, 1994, pp. 461–589), especially phonetics involves heavy fission (Staun, 2013); we sing together, but take turns in speech (Brown, 2007).

1. Audio: Shagay Kharvakh, collective dice game with singing, Mandalgovi, Gobi desert. This example illustrates how dice players spontaneously vocalize by “tuning-in,” each in his own way reflecting upon the mental activity the group is engaged in. Today such “musicking” aloud has given way to audiation, but cognitive consonance still takes place in an act of “self-other merging” (Tarr et al., 2014). http://chirb.it/PL6PJO

I see pitch organization as a unique mechanism for simultaneous processing of large number of signals with relative ease (McDermott et al., 2010a). Pitch medium is indispensable to optimizing cognitive schemes suitable for a particular environment, and reinforcing the cultural reproduction of this scheme within the community (Cross, 2007).

Instrumental for building the pre-tonal timeline is the Russian research. The Soviet regime committed enormous resources to investigation of folk cultures. During the 1940s, dedicated centers of folkloric studies were created at major conservatories, leading to accumulation of substantial databases and scholarly research. The Moscow Conservatory collection alone contains over 140,000 units of folk recordings (Giliarova, 2010). All major musicologists active in the USSR territory from the 1930s wrote on folkloric music. All graduate students in musicology and composition were required to take an ethnomusicology course and participate in field-studies.

I must underline that the goal of this paper is not to report on a theory of particular Russian scholar in his exact terms, but to present his findings to the English-speaking cognitive scientists in a format comfortable for implementation in their own research. Since cognitive science resorts to the terminology of pitch set theory, I explain all forms of tonal organization that use definite pitch in terms of set theory.

Following Wiora (1962), I use ethnic music to illustrate prehistoric music. Audio examples illustrate points of tonal organization crucial for my presentation; and to those interested in testing my writings experimentally, they indicate which music is suitable for testing. I look at my paper as a preliminary outline where many theoretic postulates might be corrected or found specific to certain conditions. Nevertheless, I feel it necessary to re-initiate in the Western science the line of research that became interrupted after the 1960s (Nettl, 2010, p. 108)^3^.

The large scope of this paper leaves little room for detailed explanations, which is addressed by provision of reference to bibliographic sources with fuller information.

## The cognitive science framework of study of tonal organization

At the foundation of cognitive study of tonal organization lies the concept of pitch set [PS]. It originates from the theory of atonal music (Babbitt, 1955). Allen Forte formalized the PS theory, defining PS as “any collection of unique pitches” (Forte, 1964). Although the original concept of PS was very specific in its reference to the order of appearance of 12 tones in an atonal composition, cognitive scientists have accepted this term in relation to any kind of music—understanding it as a set of tones used to constitute a particular music work (Balzano, 1982).

The adoption of PS elevated the importance of octave equivalence, since a set is assembled from pitches that are categorized into *pitch-classes* [PC]—presuming that all tones an octave apart represent the same pitch class. This principle sets forth another crucial concept—*interval set* [IS]: the distance between all pairs of PS tones within an octave. This distance is calculated in increments of the equal temperament semitone. Hence, the notion of PC is synonymous with *pitch chroma* (Hutchinson and Knopoff, 1978): division of an octave into 12 equal parts reduces each tone in a work to one of 12 tones, despite the original spelling of the tone in the score and its exact tuning in performance (*enharmonic equivalence* rule). Represented in this way, a PC defines an *interval class* [IC]—distance between two PCs reduced to a single representation (E/C = C/E).

PS can be transposed—thus, the sameness of IS between the original PS and its chromatic transposition forms *pitch-class set* [PCS]. Numerous music works can be based on the same PCS, and share the same *interval-class content* (Lewin, 1960)—which I prefer to call *interval class set* [ICS] (by the analogy with PCS). Such works are regarded as sharing the same tonal organization and expressive properties.

Perhaps the biggest contribution of cognitive psychology to musicology is the identification of the principal factors that contribute to the “experience” of a key (Krumhansl, 1990, p. 60). Tones contrast each other in *stability*—the sensation of a relative state of finality. Uniformity of distribution of stability/instability, with categorization of tonic, dominant, median, the rest of the diatonic, and the chromatic tones into five stability ranks (Lerdahl, 2009), constitutes *tonal hierarchy*, and defines tonality. Hierarchic organization can substantially vary, making it necessary to distinguish the stability profile of a particular PS from a PCS (Bigand, 1997).

Tonal hierarchy enables the perception of tonal melody in terms of fluctuations in *tonal tension* (Lerdahl and Krumhansl, 2007). Harmonic and melodic structures contained in music are responsible for the experience of tension in listeners (Lehne et al., 2013). Whenever unstable tones receive metric, rhythmic, dynamic, or textural stress, the listener perceives increase in tension (Krumhansl, 1996). This tension is quite objective: recent MRI study has identified the left lateral orbitofrontal cortex as the site responsible for this (Lehne et al., 2014). Metro-rhythmic leaning on stable tones decreases tension—perceived as momentary relaxation. Hence, unstable tones act as a driving force that raise “expectancy-tension” in the listener. Looking forward toward an unknown melodic continuation heightens attention for the subsequent events, which transpires into an impression of greater forward-directedness in melody (Margulis, 2005). Fluctuations in tonal tension are experienced in terms of locomotor impulses.

Steve Larson's model of “musical forces” provides a detailed framework in describing tonal “locomotion.” Drawing the analogy between mechanical laws that govern the motion of a body, and tonal laws that govern melodic motion from tone to tone (Larson and McAdams, 2004), Larson elaborates the “energetics” theory introduced by Ernst Kurth (Rothfarb, 1988). Tendency of unstable tone to resolve into the closest stable tone, Larson calls *magnetism*. Magnetism of unstable tones compliments the gravity of stable tones, generating melodic motion with assistance from *inertia:* the tendency to proceed in the direction set by the resolution of an unstable tone into a stable one. Kurth's idea that instability charges melodic motion has received experimental support: Larson and Vanhandel (2005) found magnetism to present a greater force than gravity and inertia; Vega (2003) discovered that the tendency of unstable tones to move exceeded the tendency of stable tones to stand; Hubbard and Ruppel (2013) show how gravity affects inertia.

Bharucha's (1996) notion of “anchoring” complements Larson's scheme by accounting for a harmonic grouping mechanism that binds an unstable tone with a stable tone that follows it. Music theory explains this by the integrating effect of “resolution.”

## Distinction between vertical and horizontal harmony

There is, however, an important distinction between “consonance” and “stability” (Kholopov, 1988, p. 22). *Vertical harmony* organizes simultaneous combination of tones, whereas *horizontal harmony* organizes succession of tones. Both types remain “harmony”—that is, a method of ordering the pitches according to a certain principle of euphony (pleasant-sounding combination of tones)—however, each operates on a different plane. Thus, for horizontal intervals, timbral contrast between two successive tones presents an obstacle for their integration in the same perceptual unit—whereas for vertical intervals it poses no problems (Borchert et al., 2011). The specificity of a plane causes different processing: melodic intervals *trace*—the first tone leaves a perceptual after-sound that sums with the following tone—except the interval of a 2nd. Tiulin (1966, p. 49) was first to note (1937) that a *harmonic* 2nd is a harsh dissonance, but a *melodic* 2nd is pleasant to the ear due to the peculiar short-memory phenomenon of “erasing the trace.” Komar (1971) elegantly explained this as *displacement* of the resolving tone by the resolved tone.

Larson incorporated displacement in his “musical forces” model. When the melody *leaps*, the first tone perceptually protrudes and overlaps with the new tone. If the melody *steps*, the new tone completely eradicates the previous tone's memory (Larson, 1997). Processing of melody involves the same harmonization-bias (in most cultures) as processing of harmony. The melodic progression is euphonized, when the gap between the two adjacent tones is smoothened by the mental prolongation of the first tone. Wider leaps are associated with stronger emotional connotations—perhaps, based on the speech prototype (Johnson-Laird and Oatley, 2010, p. 107). Tracing might yet serve the purpose of registering the exact size of a leap, semantically important, by caching the previous tone.

So, opposition of *tracing* and *displacement* in *horizontal* harmony should be viewed as the equivalent of the *opposition* of *dissonance* and *consonance* in *vertical* harmony. On the *vertical* plane, compliance of two tones in their harmonic spectrum determines their accord/discord (McDermott et al., 2010b). On the *horizontal* plane, stepwise progression of tones binds them into one stream of information, whereas leaps suggest bifurcation into two parallel streams (Bregman, 1994, p. 496). The leap then undergoes examination: whether it indeed marks the entrance of a new part, or it constitutes an “exclamation” within the same melodic part. Such discrimination makes all leaps “complex,” by definition, and associates them with melodic unease and tension (Rags, 1980, p. 19). “Displacement” serves as a *sequential consonance* in the progression of pitches—in contradistinction to “tracing” that works as a *vertical buffer* to compensate for disruption in the melodic smoothness (Tiulin, 1966, p. 33). Consonance is used more often than dissonance (Huron, 1994)—respectively, steps prevail over leaps (Zivic et al., 2013), especially in vocal music (Ammirante and Russo, 2015). Melodic 2nd is the principal binding agent in the music tissue (Tiulin, 1966, p. 49).

Melodically, large intervals contrast the 2nd by their capacity for stability. Each non-chromatic 2nd, as a rule, contains a stable tone^4^, whereas all other intervals can have both tones unstable. Therefore, 2nd is inherently associated with resolution (stability), whereas other intervals are not. Displacement is crucial for cadences: in melody without rests, displacement works best for resetting the “pitch integration window” (Plack and Watkinson, 2010) to mark the ultimate resolution.

Consonance/dissonance define vertical harmony, while stability/instability—horizontal harmony. Since both serve the same purpose of harmonization, they stay interconnected. In Western tradition, horizontal harmony is processed through mediation by vertical harmony. Listeners infer vertical harmonic relations upon hearing melodic progressions, and surmise the “chords” implied by the melody—in an effort to anticipate the melody (Holleran et al., 1995). This might work as a harmonic error-correction tool in verifying perceived pitch contour (Povel and Jansen, 2002, p. 83).

*Stability/instability* guides the melodic assessment—only adjusted for a consonance/dissonance relationship (Bytchkov, 1997). Musical texture, in contrary, is estimated primarily in terms of *consonance/dissonance*; only correlated with stability/instability where the intervallic content of melody mismatches the vertical harmony (as in dissonant non-chordal tones in embellishments).

## Toward taxonomy of melodic intervals

*Melodic* consonance can be defined as euphony of successive tones, and must be distinguished from *harmonic* consonance. Thus, for harmonic intervals, frequency-ratio discrimination depends on ratio simplicity: octave, 5th, and 4th are identified more easily than 7th. For melodic intervals ratio simplicity is found to have no effect (Bonnard et al., 2012). Dissonance of vertical intervals is determined by fusion. Dissonance of horizontal intervals originates from:

- the extent of melodic disruption;
- the capacity to mark the resolution.

Tones that fuse well necessarily appear melodically weak, since fusion reduces tones' autonomy (Huron, 2001, p. 19)^5^. Unison is a primary harmonic consonance, but a secondary melodic consonance. Melodic unison often falls on an unstable degree, appearing weak and giving poor resolution, unlike 2nd. That is why despite greater smoothness in pitch, unison does not match 2nd in its “gluing” power and capacity to mark a tonal center. Unison might be considered an “imperfect melodic consonance,” whereas 2nd—a “perfect melodic consonance.” This can be validated by listeners' general preference for melodic 2nd (Dowling, 1967, p. 21) and their expectation for a melodic contour to be completed by a 2nd (Carlsen, 1981).

The phenomenon of “implied polyphony” presents the best measure of melodic consonance. Whenever the melody features frequent leaps up and down, the listeners perceive two melodic lines: the upper line unites the crests of the leaping tones, the lower line—their base. This effect is not specific to Western music: also used by Japanese koto players (Burnett, 1980). The melodic dissonance of an interval is revealed through its capacity to generate an alternative melodic stream. Such testing was conducted and established the Temporal Coherence Boundary, above which segregation occurs (van Noorden, 1975, pp. 40–67). In slow tempo, minor 3rd serves as the bifurcation point, while in very fast tempo major 3rd can keep the integrity of the melodic line, delegating bifurcation to the 4th (Huron, 2001, p. 23).

2. Audio: Bach J.S. - Prelude for cello BWV 1007. Melodic consonance and dissonance. http://bit.ly/1QQmkFt.

Major 2nd champions melodic consonance^6^, followed by unison and minor 2nd—all permanently consonant. The statistic analysis of folk samples of seven nations reveals that unison and major 2nd are by far the most frequently used intervals, followed by minor 2nd, major 3rd, 4th, and 5th (25). Vos and Troost (1989) received the same results for classical and popular music.

Minor and major 3rd are consonant in faster hemitonic music. They are permanently consonant in pentatony, where they can outnumber 2nd (Kolinski, 1967, p. 14). In passages, 4th can become consonant. These intervals make a special class of *intersonance*: state of being melodically unsteady - sometimes disruptive, and sometimes not.

Larger intervals always disturb the melodic line. However, they differ in their capacity to terminate it. Octave and 5th provide a good cadence, making them an “imperfect dissonance”^7^. Tritone, 6th, and 7th produce incomplete-sounding endings. They constitute “perfect dissonance”—including melodic 6th^8^ which listeners report as high in tension (Maher and Berlyne, 1982) and difficult to identify by ear (Hall and Hess, 1984).

The following seems plausible for ranking of the melodic consonance:

$$\begin{array}{l} 2nd,unison,3rd,4th,5th,octave,6th,tritone,7th. \end{array}$$

Consonant ranking is influenced by *melodiousness* of the corresponding melodic intonation, which is a cultural factor. However, the ability to distinguish melodic consonance/dissonance appears to have genetic roots—just as its harmonic counterpart—according to the EEG measurements during newborn infants' sleep (Stefanics et al., 2009). The newborns can segregate concurrent tones into separate audio streams by detecting inharmonic relations between the co-occurring sounds (Bendixen et al., 2015).

Musicians know that melodic intervals bring about stronger emotional reaction than do harmonic intervals. Music training includes teaching “well”-tuned melodic intervals. Performers and listeners consider a dissonant melodic interval well-tuned when it is slightly wider than that which is prescribed by music theory—and this discrepancy becomes greater for larger intervals—responsible for their association with tension, harshness, and irritability (Rags, 1980, p. 19). Tracing determines larger intervals' valence. The “trace” is subject to the same rules as vertical intervals. So, melodic tritone is usually considered harsher than 5th despite being smaller. The aggregate data of all the spectral content of a particular “musical moment” is collected and converted into a rate-based code in the brainstem (Plack et al., 2014). Therefore, contribution of harmonic consonance/dissonance to melodic categorization is perhaps inevitable.

Yet another principal difference between vertical and horizontal harmony is that the concept of ISC is not applicable to melodic intervals (Tiulin, 1966, p. 49). Inversion of a melodic interval does not retain its tonal properties. Thus, 2nd is consonant, while 7th dissonant; so are unison and octave; 3rd can be consonant, while 6th is always dissonant; so are 4th and 5th.

## Virtual music space

Vertical and horizontal axes, together, define a virtual music space, where “musical forces” control the melodic and harmonic progressions within a music work. Although this reality remains “virtual” and exists only in the listener's mind, by no means should it be considered “subjective” in a sense that every listener imagines tonal tension in his own arbitrary way. Through a series of stem completion tasks, priming tasks, and continuation rating tasks, Larson (2012, pp. 212–310) was able to demonstrate uniformity in estimation of musical gravity, magnetism, and inertia amongst the listeners of tonal music. His findings are corroborated by the line of research on locomotor entrainment through music^9^.

Musical sounds are not just abstract auditory signals—they are spatial constructs that exist on a 3-D plane (time/pitch/texture) and specify fictional movement every time musical tones are bound together by tonal tension. Pitch changes generate melodic motion, where “pitch contour” and “distance” act as psychoacoustic correlates of “turn” and “displacement” of physical space (Ammirante and Thompson, 2012). Despite its illusiveness, melodic motion constitutes a fundamental aspect of music's impact and meaning (Clarke, 2001). Music is a motion-abstraction scheme that has a life of its own: “Music is an auditory fiction in which the sounds of voices or instruments are combined to produce sounds that never appear in nature” (Bregman and Woszczyk, 2004). In fact, the modus operandi of music opposes that of real life sound: the default state for musical perception is *fusion*, whereas natural sounds usually trigger *fission*.

Music is a unique and peculiar form of constructing quasi-spatial relations between auditory objects—taking after the relations of physical objects. The entrainment mechanism links the musical and physical universes. Rhythm is not the only property that connects musical and physical organizations. Dynamics is also involved in musical modeling. Dynamics contributes to the impression of relative “mass,” relying on the synesthetic connection between the perceived “size” of a sound and the actual size of the object that produces it (Marks, 1978, p. 53). The cross-modal mapping of height-to-pitch and thickness-to-pitch is already observed in 4-month-old infants (Dolscheid et al., 2014). This percept can be titled “virtual mass”: humans selectively entrain specific parts of their body to music depending on the distribution of periodic metric stress—heavier pulses engage axial body parts, whereas lighter pulses act more on lighter distal parts (Toiviainen et al., 2010).

Musical gravity imitates physical gravity. However, their correspondence is not strict. Eitan and Granot (2006) established that listeners, in their spatial representation of music, relate pitch contour to verticality, and loudness to distance and energy. But a number of cross-modal correspondences was found to work asymmetrically: descending pitch contour was perceived as spatial descent, whereas ascending contour was not nearly as strongly associated with ascent. Correspondence of increase in velocity with intensification was equally asymmetric. Evidently, musical gravity only partially follows its physical analog (Hubbard and Courtney, 2010), influenced by cultural factors and perceptual differences between senses of vision and hearing.

Musical “virtual space” should be regarded as a medium of autonomous organization that generalizes information known to an individual about the world in which he lives, and negotiates this generalization within the community of music users (Eitan, 2013). Through a series of cultural interactions music users form consensus on how their motion control and motor coordination are affected by observable physical laws—and take the established relationship as a prototype for relationship between musical tones in a PS (Gruhn, 1998).

Since musical gravity operates on principles that only partially imitate principles of physical gravity, dogmatic reliance on gravitational correspondence might lead to error. The recent theory of evolutionary origin of tonality (Doğantan-Dack, 2013) leans on universality of resolution: claiming that melodic motion is meant to end in a stable state analogous to physical unstable states, terminated by stable states. Even for Western tonality this is not necessarily the case. Ending on a stressed dissonant chord prevails in jazz/blues, setting a stereotype in popular music—altogether with unstable “vamp” fading-out. In folk practice unstable ending is just as good as stable.

3. Audio: Harvest Song, Bulgaria. Otglas (a break-off tone) marks the end by instantaneously throwing off the reference frame for stability (Kholopov, 2005). http://bit.ly/1IY0NV7

Folk-song can stop on the leading tone. Performers do it deliberately: “as though I lost my track” (Rudneva, 1994, p. 171). Unstable ending often works similar to ellipsis in punctuation.

4. Audio: Olonkho Oso Tuigun, Sakha. Ending of music on unstable tone corresponds to the standard formula of ending in Yakut epic tale: “saying this, he departed.” http://chirb.it/bb59c5

Musical forces manifest themselves not so much in cadence, but in the choice and functionality of the tones—the uncovering of which is impossible by the PS theory alone and requires the modal theory.

## Investigation of melodic harmony: mode and intonation

The concepts of PC, PS, and IS impose analytic restrictions which limit the scope of musical material that can be effectively investigated using these notions alone. Assumptions of PC are made based on harmonic analysis of a score. But folklore is oral. Many genres are characterized by continuous music-making (Maghreb *n**u**bah* can last for a few days). Where does one “song” bridge to another? And where are the two contrasting sections of the same “song”? Even ethnomusicology has not yet coined a comprehensive definition of a song (Zemtsovsky, 1983). Structural features alone make it difficult to delineate song from speech (Mang, 2000; List, 2008).

The way of universally covering tonal organization is to incorporate melodic harmony in the notion of PS. Traditional musicology addresses this with the concept of “mode.” The Grove dictionary defines mode as the interaction of certain hierarchy of pitch relationship with a certain melody type, which results in setting a compositional norm that can be understood as a “particularized scale” or/and a “generalized tune”—depending on the musical context (Powers et al., 2001). Despite its progress, this definition still has shortcomings. It reserves the possibility for a mode to be “a scale,” restricts it to a single central tone, and disregards intervallic typology. This leads to poor distinction between “scale” and “mode,” as well as “mode” and “key,” which becomes an issue when dealing with music of folk origin^10^. In general, modes have had little connection with scales until the High Middle Ages, and “then only in the minds of theorists” (Wulstan, 1971).

In Russian musicology, mode was not a prerogative of Medievalists, but a backbone for study of *any* music—at least since 1908 (Yavorsky, 1908)—including folk and non-Western cultures. Beliayev (1990, p. 225) carved the most laconic definition:

- “mode is the generalization of *types of melodic motion* in relation to *intervallic structure* of these types.”

More elaborated definitions emphasize the organic coherence of tones in a mode^11^. Russian Musical Encyclopedia defines mode as “pleasant to ear concordance of tones in their pitch” manifested in “systemic relations of pitches, united in a set by a central tone or a *group of tones*—as well as *concrete combinations of tones* that embody such systemic relations” (Kholopov, 1982)^12^. This definition puts forward the criteria of complex gravity, intervallic system, and characteristic *melodic intonations*.

“Intonatsiya” theory is another achievement of Russian musicology, poorly understood abroad^13^. Although “intonatsiya” became associated with Asafyev's name (Tull and Asafyev, 2000), who understood it as a complex semiotic and cultural phenomenon, the underlying concept of melodic “intonation” was introduced by Yavorsky (1908, p. 4)^14^ as: the “elementary unit of music structure that binds its semantic content to similar verbal intonation.” Modern research generally confirms that melodic contour, interval, and tonal organization are analogous to linguistic direction, slope, and height (Bradley, 2013), and are engaged in emotional communication—where the “audio resolution” is quite high, to the semitone level (Cook, 2002, p. 104).

Intonatsiya theory connected the abstract notion of mode to the concrete implementation of tonal order in a given music work—revealed by means of intonational analysis (Zemtsovsky, 1980) (see the sample analysis at the end of Appendix I). Asafyev (1952, p. 289) describes the structural aspect of intonatsiya—which I am going to call “intonation”—as a “tone-cell” that in its simplest form presents a 2-tone melodic interval, and possesses three attributes:

- intervallic distance;
- melodic direction;
- gradation in *melodiousness*.

The latter reflects the psycho-physiological ease of singing of a given interval, and a cultural preference for it.

Mazel (1982) elaborated the theory of “intonation” as the elementary structural unit in the organization of horizontal harmony—the counterpart of “chord” in vertical harmony. The succession of intonations comprises melody, and charges it with tension at points critical for expression. A single intonation represents a time-point in a “form-process” (the experience of changes in expression of music) while simultaneously serving as a brick in a “form-crystal” (a structure derived upon completing audition of a work)—something akin to “quantal element in musical experience” (Godøy, 2013). Thus, intonation “glues” musical structure to experience, opening gates to semantic interpretation, and mediating between memory and attention: the listener decodes melody by recognizing familiar intonations, while identifying and memorizing new ones.

Intonation charges the melodic contour with stability/instability values, pollinating the vertical harmony: traceless and tracing intervals interact with each other, creating zones of greater verticality (traces in melodic leaps) and greater horizontality (displacement in melodic steps). The contrast in traces of consonant and dissonant intervals further differentiates the melody. Music-users devise maps of melodic tension to navigate through music. The most common intonations comprise maps of standard reference within a given culture.

Musicians intuitively pick on those intonations that are important in their social group. Use and re-use of the same pool of most common pitch contours forges *melodic idioms*—fixed patterns of melodic intervals placed in the metric and harmonic space—which obtain their semantic referents through association with specific genres (Orlova, 1984). Thus, ascending anacrusis 4th characterizes a march, associated with determination and purposefulness, whereas descending downbeat 3rd characterizes a lullaby, associated with comforting, and supporting. Such correspondences were noticed by Cooke (1959, p. 89)—and received some experimental confirmation (Maher and Berlyne, 1982).

Competent music users intuitively build their glossaries of musical intonations peculiar to a given cultural context. Those glossaries merge into a mega-glossary of conventions shared by all music-users within a social group (Shakhnazarova, 1966). Entire nations can be described in terms of “intonational culture”—and in fact, for music of numerous Siberian ethnicities that is the only rational way of description (Sychenko, 2009). Each historic formation can be characterized by an assortment of particular intonations (Szabolcsi, 1965, p. 205). And frequency of distribution of these intonations shapes a mode. The ultimate selection of tones for a composition is determined by a set of intonations most important for expression in a particular genre. Typology of content leads to typology of form—crystallizing a mode (Skrebkov, 1967)—which then, in turn, starts formatting the content.

Recent exploration of statistic methods in melodic analysis supports Asafyev's claim that certain styles of music can be defined by their intonation prevalence (Asafyev, 1971, p. 281). Zivic et al. (2013) report that Classicistic melodies are characterized by prevalence of double unison—which is rather rare in the Romantic repertoire. Eitan (1993) confirms marked differences in contour typology between historic styles. Different types of music use specific “theoretically important tones” more frequently than other tones, and guide the listeners unfamiliar with a given style to the tonal organization (Castellano et al., 1984). Juhász (2012) analyzes pitch contours and segmentation of 30,000 melodies from 25 different cultures, and demonstrates significant differences between certain national types in their use of melodic intervals.

Asafyev's “tone-cell” is remarkably close to what Brown and Butler (1981) identified as a “cue-cell” in their experiments, when they discovered that listeners do not have to hear the tonic in order to detect the tonal center^15^. Quinn and Mavromatis (2011) also concluded that “pairs of neighboring harmonic states, demarcated by note onsets, are sufficient as windows for key-finding.” They specified that harmonic dissonance had no contribution to stability—rather, that the tonal center was defined by the fact that cadential progressions utilized *few* motifs that used the *same few pitches*, whereas other progressions used *many* motifs that were distributed *across pitches* transpositionally. Evidently, the knowledge of characteristic intonations helps listeners navigate across tonal maps, following the compass of tonal gravity. Huron (2006, p. 160) came closest to Asafyev, when he inferred the scheme for typical scale-degree successions in the corpus of German folksongs. He calculated the probabilities for each of the major key degrees to proceed into other degrees, and identified those for which a single continuation dominated all other possibilities—calling them “tendency tones.” What Huron discovered were Asafyev's “tone-cells” that characterize the major key mode.

## Modality vs. tonality

Key is a mode, too: the unity of its tones is generated by melodic harmony as much as by vertical harmony—“tendency tones” are not any less important for perception of tonality than are the functions of implied chords. Temperley and Marvin (2008) put this condition under test and discovered that listeners performed poorly in finding the key of a melody when it was generated by the distribution of PCS alone. Listeners needed structural cues produced by the *ordering* of tones within a sequence, to successfully define a key.

The same key can host different modes: during the 1800s string players employed two tuning standards, *Gamme europeenne* and *Gamme grecque* (Barbieri and Mangsen, 1991) that differed in their treatment of the VII degree. Both gammas represented the same key, yet presented distinctly different modes. Evidently, the difference was determined by the prevalence of certain melodic progressions: prevalence of VI–VII turned the major key into *Gamme grecque*,—while the prevalence of VI-V made it into *Gamme europeenne.* “Tendency tones” produced modal inflection.

*Every key is a mode, but not every mode is a key* (see Part-2). Hence, it is cardinal to distinguish between modality and tonality—following Choron and Fayolle (1810), who opposed their contemporary “key” to the Greek mode (Blum, 1985).

*Tonality* (in a narrow historic sense) is such principle of organization where all tones in a PCS are subordinated to the tonic and the tonic triad, and are categorized through their functional relations to one another, expressed in the formation of chords that execute functions of stability (tonic), instability (dominant), or neutrality (subdominant) in distribution of harmonic tension. Such organization is typical for classical and popular Western music, as well as more recent folk music. Major and minor keys constitute tonality—which includes the natural, harmonic, and melodic *modes* of these keys.

*Modality* can be defined as a principle of tonal organization where all tones in a PCS are united by *melodic* relations—that is, by frequency of occurrence of certain intonations and their melodic functionality: capacity to initiate, finalize, or develop melodic phrases. Such organization is characterized by weak tonicity: it is normal for such music to have multiple anchoring tones of variable gravity. If to compare tonality to electric DC, then modality would be AC: an unstable tone can turn into stable, or vice versa, and be attracted by a different tone—fluidity of such alternation distinguishes modality from tonality. Just as much as tonality is characterized by *permanence of tonic function* and abundance of alterations (sharpened/flattened degrees); modality is characterized by *permanence of scale* (scarcity of alterations) and fluctuations in gravity (Kholopov, 1975). Western music prior to the seventeenth century, and most of world music, constitute the modal domain.

Modality and tonality can coexist. Examples of this are found in music composed in Church modes after the eighteenth century, as well as in modern jazz, rock, and post-tonal classical music. The share of tonality varies depending on whether it is *harmonic* or *melodic* consonance that governs organization. Modal gravity depends on *melodic* consonance (Kholopov, 2005). The leaning tones in characteristic intonations magnetize other tones. Modal gravity is a function of rhythm and meter, frequency of repetition, and sequential position in melodic phrases (especially in starting and ending points).

## Problem of intervallic typology

The difference between modality and tonality transpires into the difference in intervallic priority: *modality* relies on *melodic* consonance, while *tonality*—on *harmonic* consonance (Von Hornbostel, 1948). This difference is not obvious. Modal music has its own taxonomy of organization, different from tonality (see Appendix I)—especially early forms of modality cannot be parsed in accordance to Lerdahl/Jackendoff theory (Ojamaa and Ross, 2011).

The biggest obstacle for applying tonal methodology on modal material is the difference in *intervallic typology*: a principle used to define the reference pitch-points in a melodic contour (Kholopov, 1988, p. 115). Intervallic typology is influenced by the tuning system and the mode, but presents its own aspect of tonal organization, deliberately managed by the creator of music—at least since the Hellenic era (West, 1992, p. 162). Greeks distinguished between 3 types: diatonic, chromatic, and enharmonic—each associated with specific semantics (Pont, 2008). In addition to the 3 Greek types, there are 5 other types (see Appendix I in Supplementary Material)—each characterized by its own expression.

The problem is that different models of tonal organization subscribe to different methods of tracking intervallic relations. Not every music system recognizes the concept of interval. Even such sophisticated music system as Indian raga does not reserve a term for “interval”: in raga, the exact position of one tone in relation to another is processed not in terms of pitch-distance but as membership in a PCS combined with a *numerical value* of the degree within a mode (Rowell, 1981). Such thinking, in fact, prevails in early folk music.

That is why it is essential to account for pitch order in PS of a mode (ascending, descending, symmetric). Three earliest forms of tonal organization use indefinite pitch and disallow application of PS framework. Six stages of it are based on non-octave interval typology, requiring adaptation of the PS theory to account for other types of equivalence.

Octave equivalence must have been discovered during the Neolithic Era, limited to selective tones, and acquired formative power in tonal organization only by the Middle Ages. Contrary to the widespread belief based on confusion over the historic transformations of the term “mode” (Cazden, 1971), Ancient Greek music was built on equivalence of not octave but 4th^16^. Aristoxenus described modulation by an *octave*—which indicates octave *inequivalence* (Hagel, 2009, p. 4). Music systems that succeeded the Greek were non-octave^17^ in their design: Byzantine oktōēchos, Daseian notation, Persian dastgah, Mediterranean and Central Asian maqam—all feature non-octave naming scheme and tetrachordal/trichordal principle of music-making.

Just like folk songs, Medieval art-music followed what Sachs (1960) terms the “*chain* principle”: their melody had a formative 3–4-tone kernel, which expanded whenever a singer became excited—adding a similar interval above/below the kernel's margin. This expansion disregarded octave equivalence, because the singer tended to leave out the distant tones and operate only on nearby pitches.

5. Audio: Samai. The chain principle: melody starts on the tetrachord Saba on D, ascending to tetrachord Hijaz on F, and further up to tetrachord Hijaz on C—where upper Db mismatches lower D-natural. http://bit.ly/1YCPVqZ

Chain principle often produces what appears as *false relation* according to Western music theory: a degree is permanently tuned noticeably higher or lower than its octave counterpart.

6. Audio: Maqam Saba. “False” relation between upper Db and lower D. In maqamat, relations between adjacent tetrachords tend to outweigh octave relationship, evident in practice of adding “false-related” leading tones at the tetrachord margins (Shumays, 2013). http://bit.ly/1KwKy5g

Unfortunately, there formed a trend in Western musicology to elevate octave equivalence to the rank of cognitive universal, and retroactively ascribe it to early stages of tonal organization, when music was governed primarily by the melodic harmony. Such are the evolutionary theories by Fink (2003) and Kolinski (1990), proposing spontaneous discovery of natural harmonics^18^ and the circle of 5ths by a hominid – following Pythagorean lineage. Pythagoreanism is inherently achronic and therefore unsuitable for study of evolution of musical perception (Cazden, 1958). 5th and 4th are melodically difficult for intonation and would have required a long time-line of development. To this day children still acquire the ability to sing them in tune after mastering 2nds and 3rds (Davidson, 1985). Until they do so they tend to scale down wide intervals to the size close to 2nd (Kvitka, 1971, p. 235)—practice observable in infants' cry-melodies (Wermke and Mende, 2009) and first songs (McKernon, 1979)—despite their ability to vocalize across a wider range (Fox, 1990). Gradual interval expansion characterizes both, infant and “primitive” musics (Nettl, 1956). Hominids were unlikely to have vocally reproduced wide intervals sufficiently precise to establish the reference pitch and stability axis. And instrumental music usually follows vocal models (Kvitka, 1973, p. 21). Examples of dichordal and trichordal folk melodies based on 4th, 5th, or octave are scarce, whereas there is no shortage of them for 2nd and 3rd (Alekseyev, 1986, p. 119). Numerous archaic cultures employ scales narrower than 4th (Jordania, 2006, p. 69, 73, 110–113, 146): i.e., Lamaholot duet singing in Flores uses no intervals larger than 3rd (Rappoport, 2011).

Simple-ratio preference is a *local* Western feature—not a universal, against some claims (Burns and Ward, 1999). Even amongst native Westerners, ability to reliably identify intervallic relations is present mostly in musically trained listeners—, many non-musicians have difficulty distinguishing even between vertical 3rd and 4th, instead, they process pitch changes primarily by melodic intervals (Smith, 1997).

Butler and Brown (1994) note that listeners “pick up information about tonal harmony from *one* or several tones *at a time* as the music *unfolds* perceptually across time”—lamenting that this phenomenon has received little attention. They identify two reasons for this:

- Assertion that harmony is intrinsically related to the harmonic spectrum of periodic tones.
- Excessive credit given to abstractions such as scale and chordal structures.

There is abundant evidence that melodic consonance plays a more important role than harmonic consonance in many cultures across the globe. There is abundant evidence that melodic consonance rather than harmonic consonance determines concordance in music in many cultures across the globe. Such is Lithuanian *sutartinë*. Its setting includes 2-part polyphonic imitations in major 2nd: one part leans on C-E, whereas another—on D-F#. The vertical harshness, however, is *apophatic*: “sutartinė” means “fitting in agreement,” requiring great peacefulness and concurrence from female singers (Raciuniene-Vyciniene, 2006).

7. Audio: Sutartinė “Lioj liepa,” Lithuania. Musical apophasis: tender melody in harsh harmony. The singers are well-familiar with the standard Western harmony, yet carry their own style. http://bit.ly/1NXok0i

As apophatic is Papuan weii, with parallel minor 2nds, described by participants as nice “bell-like.” Messner (1981) coined the term *Schwebungsdiaphonie* to refer to this dissonant music-whose wide spread spanned from Western Europe through Balkans, Afghanistan, Central East Africa to Indonesia, suggesting its origin from a vast archaic proto-culture (Brandl, 2008).

8. Audio: Oe Bala, weeding work-song, Flores Timur. Its cluster-based vertical harmony, voice quality, warbling technique, and melodic patterns, especially cadences, are surprisingly similar to Bulgarian (compare Ex.3), Bosnian, and Macedonian multi-part singing (Yampolsky, 1995a) http://chirb.it/cOLsKH

Apparently, such proto-culture prioritized melodic consonance over harmonic. Moreover, Messner (2006) emphasizes that *Schwebungsdiaphonie* often engages “maximal roughness” (80–165 cents) and the same contrasting functionality of parts.

9. Audio: Teo Ne Wea-Dioe, Ngada wrestling music, West Flores. 3-part singing in parallel major and minor 2nd is learned by the participants, part by part, as accompaniment to the bass melody, where the upper part is supposed to keep the other two “in-tune” (Yampolsky, 1995b) http://bit.ly/1MrBLBd.

The capacity to hear the difference between harmonic consonance and dissonance is most likely genetically embedded in primates (Koda et al., 2013), however, the notion of tension related to consonance/dissonance is exclusive to humans and depends on the culture. The necessity for harmonic dissonance to resolve into consonance is realized following the negative affect generated by the incongruence between pitch processing on the one hand, and melodic priming mechanisms on the other (McLachlan et al., 2013). When the melodic template (PS) heard in a piece of music does not match the modal template (PCS) known to the listener, he experiences cognitive dissonance and binds it with harmonic dissonance. That is why diaphony is possible in PCS based on 2nd and 4th.

## Pre-mode

We know that there are folk cultures without instrumental music, but there are none without vocal music. Moreover, in many cultures instrumental folk music does not serve to conserve an implicit music theory, but merely imitates the vocal models (Kvitka, 1973, p. 21). The very mechanism of sound production in wind and string instruments imitates vocal production (Terhardt, 1987). The vocal tract is designed for tonality: lung and trachea work as a primary linear resonating system, non-linear coupling occurs in glottis, and the entire vocal tract serves as a secondary linear resonating system^19^. Human pinna, ear canal, and basilar membrane are all optimized for transmission of human vocalizations, suggesting that the sense of tonal integrity evolved in response to vocal sounds (Pierce, 1992). The most biologically relevant and frequently processed tonal stimuli are those that are produced by the representatives of the same species. And human ear is remarkably effective in extraction of behaviorally relevant information from the sound of human voice (i.e., speaker's gender, age, emotional state)—testifying to the centrality of spectral data to human life (Bowling, 2012).

Anthropological evidence shows that *Homo heidelbergensis* had modern hearing capabilities as well as modern vocal anatomy, which sets the time-frame for origin of music 700,000–300,000 years ago (Wurz, 2010). Singing must have been the prime reason for the descent of larynx which enabled sustenance of pitch throughout vocalization—without dropping it, as non-human primates do (Maclarnon and Hewitt, 2004)^20^.

Why did the hominids need to upgrade their vocalization to sonorous holding of a pitch? Isn't singing in the savanna dangerous for an animal that neither outruns nor overpowers predators, and is mediocre at hiding? Jordania (2011, p. 85) notes that out of 5400 species that can sing, Homo is the only land animal—most other “singers” habituate on trees, in relative safety, and do not sing when they are on the ground. Jordania suggests a good reason for learning to sing—safety: as soon as hominids left their shelters, they could keep their predators away by loud sounds collectively made by the entire tribe. Good syncing would have been a must to project the impression of a single big creature—forming the distinguishing hominoid trait (Merker, 2000).

10. Audio: Dance of the Elephant Mask, Côte d'Ivoire. Representation of the elephant by a masked dancer and a choir in a Baule village; (Zemp, 1967). http://bit.ly/1bhwH6c

The counterpart of *collective* aggressive music-making was *individual* caretaking. A simple laryngeal vocalization, grunt, found in most primates, is a good candidate for “lyrical” proto-music—it is also employed as the earliest form of vocal behavior in human newborns (McCune et al., 1996). Grunts are the artifacts of bodily movement and physical straining (Oller, 2000, p. 251). In this capacity, grunts likely accompanied the first forms of dance—McNeill (2008, p. 16) describes a group of chimpanzees' jointly swaying and rocking to the sounds of rain. Grunting during grooming is a common behavior amongst baboons. Such behaviors could have become ritualized by hominids, with the accompanying vocalizations learned and reproduced in the absence of grooming motions (Dunbar, 2012). Then, reuse of the learned vocalization in new social settings, associated with a different emotional state, would promote abstraction of vocal expression, turning it into a *symbol* of a specific activity, and attaching to it a certain emotional denotation (Cross and Morley, 2009).

11. Audio: Tespeng Khoomei, Tuva. This introduction for a love song shows what “grunt intonation” could have sounded like. http://bit.ly/1bcHoXf

Jordania (2008) notes that humming vocalization is more wide-spread across modern population than is singing, and that this humming is probably the remnant of the grunt-like vocalizations (Mithen, 2005, pp. 221–245). Jordania explains that many animals lack a dedicated “danger call”—for them the sound of *silence* acts as a danger signal. For such species humming can serve as a “contact call,” signaling safety. Ability to hum with a closed mouth, even while eating, as well as the ease of humming, makes it favorable as a candidate for a universal safety signal. A semiotic stance obtained through contact-calling makes humming a probable prototype for musical vocalization. It is quite likely that the hominid motherese was initially hummed rather than sung—and only later developed into pitched vocalization, perhaps following suit of the caretaker in a proliferated tribe.

Rubtsov (1973) laid out the theory of song's genesis, emphasizing that it was neither physiological nor acoustic rules that brought to life tonal organization, but verbal intonation^21^. Mode is nothing but generalization of the practice of intoning by the majority within a community—sustained over an extended period of usage. And the source material for musical intonations comes from intonations of speech. The immediate cause for musical implementation must have been the need to engage a greater number of individuals in sharing the same emotional experience. By “speech” here is meant not only words, but also interjections and other utterances like weeping—capable of bearing emotional denotations without words.

- Sighing (care),
- shouting (aggression),
- narrative (neutral)

Provided three archetypes that are most contrasting to one another in their pitch contour, rhythm, and metric organization. Similar intonational prototypes are found in “cry melodies” of babies, pitch contours of which are typified by their native tongues (Mampe et al., 2009). The formative role here is played by vowels that map to similar sites in auditory cortex as pitch (Lidji et al., 2010; Gutschalk and Uppenkamp, 2011).

Initially, musical proto-intonations could be fixed to specific utterances, but then they obtained their own semantic significance and became re-texted. The moment the meaning of a vocalization was decided not by text but by typological melodic contour, was the birth of song (Rubtsov, 1962).

12. Audio: Funeral lament, Tuva. Melodic contour of indefinite pitch, which carries its dedicated emotional expression. http://bit.ly/1F3B40h
13. Audio: Kilamê ser, Yezidis, Armenia. The remnant of proto-language must be the tradition of “melodized speech,” that is reserved for expression of negative feelings amongst the Yezidis—in contrast to positive feelings expressed in songs (Bretèque, 2012). http://bit.ly/1e4P8Ms

Multiple folkloric traditions all over the world employ *formulaic* organization of melody independent of lyrics. In fact, some cultures do not employ lyrics at all (Abkhazian, Georgian, Chuvash, Udmurt), instead, they use meaningless syllables or base an entire song on a single word—(Zemtsovsky, 1983).

14. Audio: Lullaby, Tuva. Use of vocables (hushabye); (Alekseyev and Levin, 1990). http://bit.ly/1O5Wyde

Such detachment of singing from speaking typifies substantial stock of early folk music—and is still evident in the existing practice of re-texting the same melodic formula with different, completely unrelated, lyrics—found in many traditions. Thus, numerous Dagestani, Tartarian, and Evenki songs receive different lyrics every time a tune is performed (ibid.)^22^.

Repetition of familiar melodic formula, laid on unfamiliar text, is likely to create a semantic clash, when the semantic content associated with the music would push the interpretation of new verses of text in the direction away from their verbal meaning. Clashing, in fact, could very well be the re-texting goal: testing the power of melodic formula by imposing it on unrelated textual material.

Identification of a song by its melody rather than by its lyrics in such cultures confirms the prominence of melodic formula that should be viewed as musical implementation of *ritual* (Zemtsovsky, 1987). Any ritual is a culture of action—an algorithm of strict repetition in a prescribed order, applicable to histrionics, phonation, and religious thought. Fragmentation of a peculiar melodic contour and accurate reproduction of it from different pitch levels, and on different utterances, constituted an important achievement for human civilization. Ritualization of a melodic contour marked enculturation of semantic content peculiar to music—it was the birth of strictly musical cognitive typology, alternative to typology of speech, and a starting point in tonal organization—in the absence of fixed pitches.

15. Audio: Aije, Brazil. Sacred bull-roarer music of Bororo Indians, performed by Tugarege men as part of Death rite, while women and children are hiding in the huts (Canzio, 1989). http://bit.ly/1FYpqQj

An important reason for intonation to bifurcate into speech and music, evident in the opposite valence of high and low pitches for speech vs. music (Ilie and Thompson, 2006), must have been the issue of *cognitive dissonance*, as explained by Perlovsky (2012). Conceptually oriented, verbal language tends to bring to awareness discrepancies between interests of different language users, since linguistic processing occurs in terms of opposites (in order to define a concept we have to envisage what it is *not*). Music users, on the other hand, tend to share a common emotional state and the same mental attitude toward the goals of a musical behavior in which they are collectively engaged. Hence, linguistic semiosis is prone to generate cognitive dissonance, whereas musical semiosis—to resolve it. Music counterbalances language in pragmatics of communication: music focuses on “affective meaning,” whereas language only accounts for it (Gussenhoven, 2002).

Development of music compliments the development of language. There is some experimental support for “consonance effect” of music (Masataka and Perlovsky, 2012). Also, 6-month old infants display different reaction to music vs. speech: they babble, point, and move in a way suggestive of their attempt to socialize in response to speech—but not to music, which causes them to quiet down and listen (Fais et al., 2010). Perhaps, children are born with the knowledge of what constitutes sounds of speech, and what—music. Such suggestion is not unreasonable (Papoušek, 1996), since the ability to discriminate between relevant and irrelevant sounds is essential for survival right from birth. The ability to distinguish speech from non-speech is functional at the time of birth (Winkler et al., 2003), and segregation of musical sounds seems to follow suit (Háden et al., 2015).

Yet another distinction is the disposition of language toward rapid change, vs. the conservative tendency of music: there are numerous examples of ethnicities that lost their original tongue yet retained their unique music—which should be explained by the music's power to continually reaffirm one's connection to the group (Grauer, 2007)—a form of “cognitive consonance.” Comparative musicology has revealed cultures where music traits remained essentially unchanged over extremely long periods of time, wide geographical areas, and different environments (Grauer, 2007).

Opposition of music to speech is manifested in the manner of sound production. Musical vocalization usually reserves the register and spectral characteristics, contrasting to phonetics of the language native to the singer (Presentation 1 in Supplementary Material).

As contrasting is the manner of vocal articulation between the two: frequent caesuras and emphasis on phrasal ends in speech, vs. few caesuras, generous ornamentation, drastic timbral transformations, vibrato, and pronounced pitch-bending in early music (Graf, 1967)^23^.

16. Audio: The 4-year-old light tan horse, praising song, Mongolia. Deep throat singing. http://bit.ly/1DqAPad

Artificiality of sound production in such singing prompted to characterize it as “timbral” (Sheikin, 2002, p. 245) because of the prominent role of timbral inflections, often of onomatopoeic nature^24^.

17. Audio: Geese Katajjait, Canada. Vocal imitation of the geese cries. http://bit.ly/1O63ywe

Even non-alive objects could be imitated in sound.

18. Audio: Borbangnadyr, Tuva. Vocal imitation of the sound of the brook (Levin, 1999). http://bit.ly/1D36LSJ

Opposition of melodic intonation to speech was also achieved by deliberate flattening of the pitch contour and excessive rhythmisizing.

19. Audio: Katajjait, Baffin Land. Monotonous style of singing on stressed rhythmic pattern of the vocables. http://bit.ly/1Ga2lja

Many ethnicities of Siberia, Far East, and Amerindian tribes use *personal songs* to spiritually represent an individual^25^. Sheikin emphasizes that it is not the configuration of pitch and rhythm that makes such song personal, but specifically the *manner of vocalization*, where timbre plays a pivotal role. The “owner” is recognized by his spectral signature—in the same way we recognize a familiar speaker—but expressed in an exaggerated style. Songs of Chukchis, Koryaks, Yukaghirs, Evens, Nganasans, Entses, Nenets, Mansies, and Khants are all personalized in this way, while reflecting the regional differences between different colonies. The Ancestor Cult, common across the entire Siberia, contributes to formation of musical styles—because one's individual song tends to stay close to his father's song.

Like family name, individual songs were often inherited. Ojamaa (2002) describes how in infancy, along with the name, the Nganasan child receives a brief song descriptive of his personal traits from his parents. Upon reaching adulthood, every Nganasan youth creates an individual song that accompanies them throughout their life. Their acquaintances know that this melody represents its owner, and often sing that melody while thinking about him/her. In parallel, the adult Nganasan may use his parent's song as a family memorabilia. Often such song carries signs of ethnicity or geographic origin of the family ancestors through its melodic features.

20. Audio: For Topahti, Nootka song of Kwaktiutl origin. An inherited ceremonial song, given as a dowry, and permitted for performance only by its owner (Halpern, 1974). http://bit.ly/1DZ5TlS

“Personal song” appears to represent a *virtual self* : an imaginary twin-person used to emotionally examine the interaction between the self and the environment as though from aside. A comparison of personal songs by the same performer recorded at different times shows great variability in text and emotional states, but permanence in melodic structure (Ojamaa and Ross, 2004), suggesting association between “self” and melody. Amongst a number of Siberian ethnicities, personal song functions like “passport”: different melodies represent the same individual in childhood, adolescence and old age—often also carrying information about his family and birthplace (Novik, 2004, p. 80).

The initial division of proto-music on “militant” hunting vociferation and “lyrical” caretaking grunts upgraded into two proto-genres: collective “for-others” and individual “for-oneself” (Alekseyev, 1986, p. 12). Songs “for-others” were consumed collectively, and promoted the development of tonal organization. Songs “for-oneself” remained frozen in their morphology, as revealed by comparative analysis of Siberian field studies over the last century (Alekseyev and Nikolayeva, 1981). The reason for such conservation was the self-communication functionality: the singer remains half-conscious of his performance, humming a tune in spontaneous release of his emotional energy rather than trying to “convince” listeners. Sheikin (2002, p. 304) nicknames personal singing tradition as “Cartesian”: “I sing therefore I am.” The manner of such singing reminds of “safety signals” employed by social animals.

21. Audio: Xöömei on Horseback, Tuva. Spontaneous singing while riding. http://bit.ly/1JWKwm7

Little need in perfection of musical communication discourages variation and innovation, preserving “song for oneself” in inherited from ancestors state, making it a monument of early tonal organization.

## Khasmatonal mode

The main formative principle in early individualized singing appears to be *khasmatonal*^26^ interval organization (Wiora, 1959), characterized by the stressed leaps (4th or larger), which are fixed for a particular registral span in a mode. Usually, a register with a bunch of close pitches opposes a register entered by a leap. Sometimes, mode includes two leaps.

22. Audio: High song, Bulgaria. Today there are no purely khasmatonal songs in use, and khasmatonal leaps are embedded in pitched context. http://bit.ly/1EhypRM

Russian ethnomusicology holds khasmatonal organization as the first genuine type of tonal organization—tones half-spoken/half-sung, with *intense timbral/pitch modifications*^27^.

23. Audio: Menerik Yryata. Trance-song, Sakha. This reproduction of a song of a psychotic woman, sung by her repeatedly in semi-conscious state must be representative of khasmatonal style—with its glissando, vibrato, leaps, talk (Alekseyev and Nikolayeva, 1981, p. 58). http://chirb.it/vmIwaf

MRI measurements demonstrate that while listening to a song the brain is sensitive to discrete pitch changes in singing as opposed to gliding pitch in speech (Merrill et al., 2012)—a likely mechanism to promote khasmatonal leaps.

It is arguable whether or not a strictly pitchless khasmatonal mode contains “degrees,” because every occurrence of the “same” (by lyrics and contour) musical tone is tuned differently. What constitutes “sameness” here is the successive order of a tone in a melodic contour which imposes a specific function of starting, terminating, climaxing, or supporting a particular tone within a melody—prompted by registral position (Alekseyev, 1976, p. 120). Therefore, khasmatonal tones are in fact correlated “in pitch,” which makes them a peculiar form of degrees.

The main idea behind khasmatonal melodies is timbral contrast and variation. The pitch here merely supports the timbre: melodic steps accompany timbral variation, while leaps—timbral contrast^28^.

24. Audio: Night chant, Navajo. Falsetto contrast. http://bit.ly/1O68q4s

A noteworthy chasm occurs as a result of abrupt timbral/pitch change, and serves as *principal* means of tonal organization. In the absence of fixed intervals and pitches, the contrast between registers remains the only strictly musical structural parameter usable for coordination of musical tones and their integration into mode. The other two—rhythm and music form—originate from lyrics. Syllabification of melodic line is confirmed to serve as grouping tones together (Sundberg, 1992), by turning stressed syllables into tonal anchors.

Khasmatonal intonation was born the instance the majority in a hominid tribe began recognizing the *same* timbral color applied to the *same* melodic contour in the *same* vocal register—memorizing the spectral characteristics and the approximate frequency of that vocalization as a signal. Most likely this happened during the Middle Pleistocene, in parallel with the newly developed ability to recognize unusually shaped or marked stones as “special” (Dissanayake, 2013). Mammoth bones painted with ochre were found at Mousterian sites (Demay et al., 2012). Straight lines, engraved on stone tools, dated between 350 and 250,000 BP, are characterized by rhythmic distribution: equality of size, intervals, angles (Frolov, 1992, p. 74). The skill of turning “ordinary things” into “extra-ordinary” is no different than turning “ordinary” sounds into “extra-ordinary.” And shaping timbre, pitch, and rhythm works essentially in the same way ochre helped cover familiar objects with attractive ornaments.

Vocal music presided in shaping the musical mode at its cradle. Individual song must have set the standard for the musical use of voice—in contrast to speech. Primitive instruments readily available to hominids before the Middle Stone Age did not allow individualization of timbre on the range of pitches. Sheikin (2002, p. 46), overviews over 150 instrumental types used by 31 Siberian ethnicities, and infers two characteristic traits: *commonality* of objects used as musical instruments and their *dispensable* use. Tuvans insert a twig in their mouth akin to a Jew's-harp; Yakuts hold wood chips by their jaws; taiga ethnicities whistle through the bark (116)—such “instruments” are discarded after a single use (which explains scarcity of archeological finds). Siberian folk instruments in modern use have changed little from the ones found in Neolithic settlements in middle Lena region (Sheikin, 2002, p. 86). Similar indication comes from comparison of records of the first ethnographers who visited Siberian region, with the current findings (Ojamaa, 2005).

First instruments were used to imitate sounds of nature—from “realistic” birdcalls or wind emulators, such as Tuvan xirlee, to more “abstract” xomuz.

25. Audio: Symysky call, Khakassia. Imitation of the cry of the male maral made by symysky—a piece of birch bark. http://chirb.it/8zt1tw
26. Audio: Pyrgy call, Khakassia. Imitation of the cry of the wild baby-goat made by pyrgy—a wooden cone. http://chirb.it/aegPcy
27. Audio: Xomuz imitating water stream. http://bit.ly/1DrvnDR

Commonality of an instrument and its timbral idiosyncrasy typify all archaic organology. Each object as though possesses its unique recognizable “voice,” discovered by accident, from everyday usage.

28. Audio: Sukute, Solomon Islands. Struck and occasionally blown bamboo tubes. http://bit.ly/1L9FJ5m

What keeps such an instrument alive is the uniqueness of its voice. Just as a person is recognized by the sound of his voice, archaic instruments are recognized by their “personal song.” When interviewed by ethnographers, instrument makers could not give their reason for the choice of specific size and makeup in construction of an instrument—they took common objects “as they were” (Sheikin, 2002, p. 160): a leaf, a stalk, a wooden chip made during cutting of a tree, or a common tool like a bow. This music seems to originate from “playing-for-oneself” just as in “singing-for-oneself”—half-consciously, and as self-entertainment. Once the unique voice of an object is discovered, it is preserved through reproduction on other dispensable objects of the same class—very much like the contour formula of a “self-song” is repeated by different singers from different pitches. Archaic instrumental music is as formulaic as the archaic song.

Similar to two flavors of lyrical and militant vocal proto-music, instrumental proto-music also had its aggressive counterpart. Almost all the oldest instruments known amongst Siberian peoples were, in one way or another, originally related to hunting, and retained mythological connections to aggression^29^. Lawergren (1988) explains that earliest musical instruments either looked similar to weapons, served as signals between hunters, or used to frighten animals, and/or attract them in order to trap them. Jordania tells how musical instruments could be useful for scaring away predators in order to scavenge on the prey killed by them—revealing common etiology between hunting “instrument” and music “instrument” (Jordania, 2011, p. 102).

Not all applications of hunt-related music had to be loud and scary. Mastering the art of imitation of an animal's sound meant gaining control over that animal. Also, for a human to be able to produce “non-human” sound was a form of “super”-natural experience. Quiet music representative of hunted animals could have easily been an object of cult similar to the petroglyphs of hunted animals: it is not accidental that the greatest number of pictures are found in the most resonant cave areas—in Paleolithic French (Reznikoff, 2008) and Neolithic Spanish caves. Furthermore, acoustic measurements suggest that the painted wall was intended as a sound-reflecting surface (Díaz-Andreu and García, 2012). Placement of open-air rock art also seems to comply with the sound design concerns, evident in Didima Gorge, South Africa (Mazel, 2011), and canyons in Utah and Arizona (Waller, 2006).

If a cave or a megalith was selected for its acoustics conducive to human vocalization, then music must have been part of important daily activities back then. Likely, it was music that inspired artistic expression: earliest musical instruments predate the earliest known cave art (Morley, 2014). It seems that the generalization that less artistic species, Neanderthals, were supplanted by more artistic species, Homo Sapiens, is in fact accurate (Pettitt, 2008)^30^. Greater proficiency in arts and music must have contributed to the development of social-cultural systems that put Homo Sapiens at a biological advantage as compared to Neanderthals (Conard, 2011). Symbolically mediated social systems allowed to expand social networks, thereby reducing personal risk, and music performance helped build and calibrate mechanisms for emotional mediation between an individual and a social group.

Cave culture served as a powerful catalytic factor that contributed to the radical acceleration in genesis of music. Living in near total darkness puts a much stronger importance on hearing. Many archeological megalithic sites were found to exhibit a primary acoustic resonance at 110 Hz peak—which is close to the average fundamental frequency of an adult male voice (Devereux, 2006). Resonance and echo aids navigation in complex cave structures. Greater attention to auditory detail could have stimulated more intense tonal development. Reznikoff (2004), who conducted extensive research of cave culture around the world, is convinced that cavemen constantly used vocalization as a sonar method to prompt locomotion in darkness, and placed marks on the walls in spots where resonance was most noticeable—which led to the emergence of cave art. Reznikoff rightfully stresses that vocalizing in a chamber with strong echo would necessarily amplify the vertical harmonic aspect in horizontal harmony by extending the reverberation and increasing tracing in melodic intervals. Therefore, the intonations that were cultivated outdoors would have transformed their sonic properties: consonant horizontal 2nd suddenly turned into dissonant vertical 2nd. Echo would encourage leaps over steps, favoring such leaps as harmonious 5th and octave. Echo could very well be the primary reason for promotion of khasmatonal music.

Lithophone music could have provided the model for frequent continuous leaps in the melodic line—which are quite unnatural for speech. Many Paleolithic caves in France, Spain, and Portugal contain stalactites painted and covered by marks—which emit pitched tones once they are hit with a stick. It is very possible that cavemen accidentally discovered that rocks had a “voice,” too, and decided to use them to support their own singing. Most lithophones that are within reach of one another generate pitches separated by a leap.

## Genesis of pitch

Singing along with the lithophonic music would encourage singers to tune up their voice and match the stalactite pitch—following the same tuning instinct that governs vocal imitation in primates and cetaceans (Mercado et al., 2014). FMRI testing of singers' performance in response to the accompanying tone which shifted in frequency demonstrated that singers had voluntary control of their voice when the shift was over 200 cent (=2 semitones), but engaged in involuntary pitch-matching response when the shift was 25 cents (Zarate et al., 2010). It is possible that early humans had rougher discrimination of pitch, and involuntarily matched intervals in the order of a semitone (see Part-2).

Dams (1985) undertook a field study of “singing rocks,” and reported the following lithopone scales: F-C-Eb (Roucador), B-D-E-G (Cougnac), C-Eb-F-G-A-C (Nerja). Perhaps, the hexatonic Nerja scale could be the result of human interference: carving the stalagmitic edge to tune a rock higher to his liking^31^. Lithophones could have triggered aspiration for mode making in humans, materializing the concept of pitch, and supplying non-vocal intonations.

Sheikin (2002, p. 30) believes that the first intonations were “*psychophysiological”*: “natural,” determined by human anatomy and cognitive algorithms that originated from everyday non-musical behaviors. The pre-modal singer discovered capacities of his voice by experimentation.

29. Audio: Assalalaa, Baffin Land. Children game that involves singing until exhaustion of a single breath while heavily wiggling one's body (Nattiez, 1976). http://bit.ly/1FZ2a4J

He learned how to add whistling, growling, and hawking components to a sustained vocal tone (to differentiate it from speech).

30. Audio: Katajjait solo, Hudson Bay. Intense use of timbral variation. http://bit.ly/1F4PL35

These sounds were formatted according to the rhythms of heart-beat and respiration, inherent curves of acceleration/deceleration of the locomotor motions (Honing, 2003), and extraneous rhythms typical to the environment.

31. Audio: Marido paru, Brazil. Bororo work song illustrates rhythm of flint knapping as a prototype organizer of early music (Zubrow and Blake, 2006). http://bit.ly/1HJNHOS

Repertories of common vocal intonations were imitated on early instruments.

32. Audio: Xomuz, Tuva. Imitation of the Khoomei tune on the Jew's harp. http://chirb.it/efber4

The echolaliac instincts motivated attempts to imitate environmental sounds on instruments.

33. Audio: Igil Fantasy, Tuva. Imitation of horse's neighing and trotting on igil, a 2-string fiddle (Levin, 1999). http://chirb.it/1NDkpE

At this point, *organophonic* intonation—a “song” typical for the voice of particular instrument—was formed. New instrumental intonations were incorporated in an *accompanied song*.

34. Audio: Vocal imitation of animal calls, the sounds of chomuz and drum, along with instrumental accompaniment, Tuva. http://chirb.it/4w3Gge

In the reverse loop of influence, the brightest instrumental intonations prototyped the vocal ones. Thus, Croatian flat nasal *tarankanje* singing style imitates the sound of sopile (Boersma and Kovacic, 2006). Notable was the influence of chomuz on the Siberian and Mongolian singing styles (Alekseyev, 1976, p. 107). A resonant fundamental tone of chomuz must have modeled “tonicity” in Khoomei songs.

## Ekmelic mode

Kharlap (1972) traced the interaction of melodic line with folk lyrics and identified the influence of verbal rhyming on rhythmic parallelism. Rhyme's impact on rhythm shapes the intonation. Rhyme in itself contains important musical component: reciting poetry differs from prosaic speech by expanding the vowels, especially in stressed words, using vibrato and increasing harmonic periodicity in the spectral content of voice—all the features typical for singing (Nazajkinskij, 1972, p. 261). Moreover, rhyming reproduces the same intonation at the end of the rhymed strophes. When musical intonation duplicates parallel rhyming of the lyrics, it marks the rhymes with the *same* pitch, making it perceptually stand out. If intonational stress falls on a stand-alone rhymed syllable, the corresponding pitch obtains the quality of stability. Since cross rhyming is exceedingly common in folklore, musical mode inherits from it *alternation* as a formative principle: pitches in such early song, unlike tonality, are united not by tonal *subordination*, but by tonal *coordination*. One stable tone serves to counterbalance another—each magnetizing a bunch of satellite unstable tones.

Western researchers of prosody also uncovered ties between intonations of speech and music in early monuments of epic poetry and religious chant, across different languages (Cable, 1975). Each language seems to have an assortment of a few rules for conversion of the phonological accents into the melodic pitch-formula, where syllables with greater linguistic stress are set to higher melodic tones. Then, fixation of selected tones in pitch—and strict observance of 3–4 pitch classes throughout the narration becomes a means of hierarchic tonal organization: a way of converting the metric order of words into pitch order of tones. In essence, epos and chant *organically* produce musical modes^32^.

The most thorough theory of origin of pitch organization in an early mode was laid out by Eduard Alekseyev. Based on his life-long research of his native Yakut music and neighboring Siberian cultures, Alekseyev identified what appears to be the earliest form of mode with an IS. Such mode is characterized by unfixed tuning of all degrees, where some degrees show more permanence in their tuning, presenting less pitch variants upon their reproduction within a song—as compared to other degrees.

Kholopov (1988, p. 117) proposed the term ekmelic^33^ to refer to a mode whose PCS includes tones that are unfixed or variable in pitch.

- Melodic consonance,
- scarcity of formulaic intonations, and
- close correspondence between rhythm of the lyrics and musical rhythm (limited sing-out)

—altogether generate a sense of unity that binds the tones of ekmelic song into a mode.

Rhythmic organization in ekmelic music is strictly regular, even monotonous—to compensate for looseness of pitches (Alekseyev, 1976, p. 52). Repetition of the same musical formula for each strophe of lyrics characterizes the oldest Yakut genre, monodic epic olonkho. However, repetitions affect only the melodic contour—exact pitches substantially vary. The very same performer, when repeating the same song, sets the same lyrics to varying pitches unaware of pitch discrepancies. When interviewed, he refers to multiple melodies as “the same” in music structure and musical meaning—and his listeners also share this conviction. Similar isomorphism was found by List (1987) amongst Hopi Indians.

The mathematical problem of defining unfixed ekmelic intervals is best resolved by counting not the absolute distance in pitch, but the numerical order within the mode (Alekseyev, 1976, p. 123)^34^. Below is my realization of Alekseyev's taxonomic idea.

Ekmelic unison is a reproduction of the “*same*” degree (with possible wandering up or down).

35. Audio: Song of praise to the horse, Mongolia. Unichordal song based on a single degree—probably due to the rhetoric effect of listing all the virtues of the horse that just won the race (Desjacques, 1991). http://bit.ly/1yJDVKI

Ekmelic 2nd is the *complimentary*^35^ relationship between adjacent degrees, *different* in their melodic function (i.e., one leaning, and another supporting).

36. Audio: Old Woman's Song from olonkho Mighty Er Sogotokh, old epic Yakut style. 2-degree mode a 2nd apart (complementary relation). http://chirb.it/3cNa11

Ekmelic 3rd is the *opposing*^36^ relationship between two tones (adjacent or “over the tone”) of the *same* function (both leaning, or both supporting).

37. Audio: Baianai Yryata, Algys (invocation of taiga's spirit), dyiretii style (the oldest epic style of Yakut music). 2-degree mode with the ekmelic 3rd between adjacent degrees (opposing relation), responsible for shifting of the upper degree (Alekseyev and Nikolayeva, 1981, p. 67) http://chirb.it/JABE04
38. Audio: Usuiaana ebekkem (Song about Ust'Yan), a Sea chant from the coast of the Laptev Sea, old style. 3-degree mode with the ekmelic 3rd between I and III degrees (opposing), with II degree complementing the III (numeration proceeds in ascending pitch order). The II and III degrees keep shifting together (Alekseyev and Nikolayeva, 1981, p. 66). http://chirb.it/NpN5D5

Ekmelic 4th is the *extreme*^37^ relationship between non-adjacent degrees of *different* functions—unbound by resolution.

39. Audio: Bisik Yryata. 3-degree mode with the following intervallic set: ekmelic 2nd between II and III degrees (complementing relation), 3rd between I and II degrees (opposing), and 4th between I and III degrees (extreme)—especially the I degree strongly shifts down. http://chirb.it/zJGLkG

According to Alekseyev, ekmelic music hardly includes more than four fixed points, and therefore cannot present more than four functions (leaning, supporting, opposing, or extreme). There is no 5th in ekmelic ISC: when Yakuts encounter a 5th (filled up by 3 degrees) in Russian songs, they regard it as “foreign” (85).

Modal functions determine gravity in ekmelic mode. *Complementing* (supporting/leaning) and *neutral* (supporting/supporting) degrees retain their distances.

40. Audio: Bytta-bytta Maaryiabyn (“Beautiful Mary”), lyric song. 3-degree mode is made by adding two complementary 2nds—without forming the 3rd between the I and III degrees. As a result, none of the pitches shift. Ekmelic 3rd is not always equal to 2nd + 2nd. http://chirb.it/5mOz2N

*Opposing* leaning/leaning degrees become repelled, and tend to increase their distance throughout the song (126). The same applies to *extreme* supporting/leaning degrees.

Morphological and statistical analysis of such songs, conducted by Alekseyev, reveals the mechanism by which degrees become fixed in pitch, and subsequently shape the mode (129). It involves intonations that turn into formative *motifs*: they determine musical arrangements by virtue of articulating respiration and parsing of lyrics. Word(s) sung on a *single* breath is perceived as a *single morphological unit* by the ekmelic singer. Fenk-Oczlon and Fenk (2009) confirm that the breath cycle shapes perception of both, verbal and musical intonations.

Alekseyev identifies two earliest types of motif-intonations: *ascending* and *descending*. The ascending type assigns stability to the *initial* tone because of trochaic meter that overwhelms Yakut songs.

41. Audio: Dyakhtary Tuoyuu, Love song, ascending inclination http://chirb.it/KMFzky

The descending type leans on the tone that marks the *completion* of the contour's fall, when it slightly rolls up.

42. Audio: Tuul Yryata, Song in sleep, descending inclination http://chirb.it/znFtxL

Change in melodic direction (in conjunction with metric stress) marks the anchor point—causing the singer to stress the corresponding tone by fixing its pitch (in contrast to the rest of the tones). Majority of ekmelic songs contain two anchors, because the overall melodic motion in a song follows a sinusoid curve, where intonations only differ in phase. The sinusoid shape of ekmelic melodies contrasts the zigzag tendency of khasmatonal melodies. Ekmelic waves provide the most comfortable regulated manner of controlling the pitch. The ongoing oscillation by the same wavelength presents predictable and manageable model for ordering the pitches.

**Figure 1:**
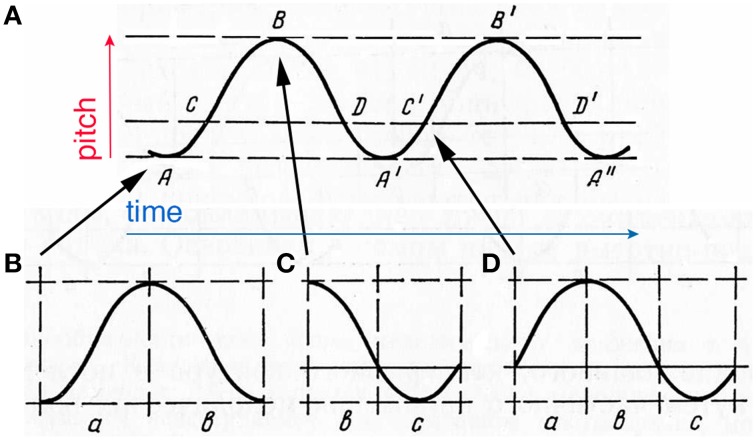
**Sinusoid melodic line and phasing of the ascending/descending phrase-intonations**. The horizontal dashed lines show the placement of the anchor tones in relation to the sine. **(A)** Typical starting and ending points for the melodic contour of the following varieties of melodic formulas: initial ascending *A-B, A-D, A-A1, A-C1*; initial descending *B-A1, B-C1, B-B1, B-D1*; initial wave figure *C-C1* and *C-B1*. The letters for pitch points reflect functionality of pitches: letters *A* and *B* represent marginal pitches, and *C* and *D* - intermediate pitches in a 4-degree ekmelic mode. **(B–D)** Melodic contours of typical ekmelic motif-intonations that comprise phrase intonations indicated by black arrow. The vertical dashed lines indicate the margins between the motif-intonations: *a*, ascending motif; *b*, descending motif; *c*, concave wave motif. This figure is based on four figures from “The Problems of Genesis of Mode” by Alekseyev (1976, p. 134). Used by permission.

Each song consists of multiple cyclic repetitions of stereotypical formula that usually corresponds to a phrase in the lyrics. There are three options for the formula's start: at the trough, at the peak, or slightly pass the trough (respectively *A, B*, and *C*, Figure 1A). The ending points are also well defined (*D, A1*, and *C1*, Figure 1A). These points are likely to house fixed degrees of ekmelic mode. Most Yakut songs are built on the framework of two degrees, unless a longer formula leaves space for the third degree.

The reversal of direction defines the margins between the motif-intonations within a formula: *a* ascending, *b* descending, and *c* wave-like intonations (Figures 1B–D). The configurations *a-b, b-c* and *a-b-c* are most common.

43. Audio: Personal song about the native land, Amga region. Wave-like *c-b-c* formula. http://chirb.it/M7Betn

Greater expenditure of air and muscular effort in ascending singing ties up ascending type with buildup of tension, and descending—with relaxation. Their contrast generates melodic consonance/dissonance:

- Tones that follow a low leaning point (A) become associated with instability and tension.
- Tones that follow an upper leaning point (B) become associated with resolution.
- Leaning point of the ascending type (A) obtains greater gravitational value as compared to the leaning point of the descending type (B).

Alekseyev qualifies such functionality as genesis of the first true modality, and speaks of ascending and descending intonations evolving into modal “inclinations”—in analogy to major and minor inclinations of a key. With the passage of time, the *ascending inclination* developed into *authentic mode*, while the *descending*—into *plagal*, both of which opposed each other semantically. Each ekmelic inclination is determined by the *opening* of the melodic phrase—in total opposite to tonality, where the *ending* determines if the key is major or minor.

As singers developed a sense of coordination in pitch, they explored the idea of going over a degree. This produced a zigzagging melodic contour—which became affiliated with genres of dance, jocular song, and tongue-twister.

44. Audio: Song of the Virgin Abaasi (comically clamorous underworld spirit), from olonkho Urung Aiyy Toyon. Zigzagging formula. http://chirb.it/10PGGL

Next came the idea of skipping over two degrees—very different from khasmatonal leaps. It observed the sequential order of degrees—rather than arbitrarily skipping into marginal registers. Energy, contained within a leap, favored ascending direction. The extra effort expended into such leap prompted an immediate fall in pitch. This is how the fifth melodic type came into being: ascending leap followed by a descending fill-up. This completed the set of five melodic standards of ekmelic music (138).

45. Audio: Devil virgin's song, from olonkho Mighty Er Sogotokh. Leaps characterize the evil character (Alekseyev, 1996). http://chirb.it/47cHHO

It appears that each of these melodic contours is cross-modally connected to spatial perception of vertical height, and associated with a particular emotional state (Hair, 1995). Two experimental studies of pictorial shapes (Lundholm, 1921; Poffenberger and Barrows, 1924) discovered that gradual descending curve is associated with sad/lazy/weak adjectives; gradual horizontal curve—with quiet/gentle; medium rising curve—with merry/playful; whereas steep rising curve—with agitating/furious adjectives.

The greatest specialty of ekmelic mode is that it is *scalable* (“unfolding”) (Alekseyev, 1976, p. 148): intervallic distances between tones can be proportionally increased or decreased, from semitone to tritone. Transposition of a song often invokes “logarithmic” scaling of intervals toward the upper register. When the singer is asked to sing the same song higher, he compresses its intervals to a smaller compass (Alekseyev, 2013).

46. Audio: Sae Dyige-dyige, comic love song of a woman who has many lovers. Two performances of the same song by the same singer: ambitus of (1) 4th and (2) 3rd. http://chirb.it/g36sC2

Many ekmelic melodic formulas demonstrate the tendency to gradually expand the utmost high and low anchors in the singer's compass further away from the fixed center (Alekseyev, 1976, p. 50) (see the end of Appendix I). Alekseyev compares this effect with the absence of gravity in cosmic interspace (162): when the gravity of anchor points is weak, the tonal inertia can push the marginal tones “out of orbit.” Musical weightlessness manifests itself as relative lack of tonal tension.

47. Audio: It Was a Very Lovely Day When the Water Was Calm, Inuit personal dance-song, Alaska (Boulton, 1955). Series of leaps reduce tonal tension. http://bit.ly/1J1APVV

Similar scalability is found in Nenets (Ojamaa, 2003) and Pueblo Indian music (List, 1985). Sachs (1962, p. 64) noted that shrinking/expanding steps characterized Amerindian music that had no scale-wise tuned instruments. Proportional expansion of ambitus was found in Aboriginal music (Will, 1997). Mpyemo use scales with “mobile degrees” that are re-assigned pitch values in the process of a song (Arom, 2004, p. 25). “Elastic scales” are described by Kubik (1985). Yasser (1932) conceptualized “sub-infra-diatonic scale” (142) based on three “regular” degrees 5th and 4th apart, and “auxiliary” scalable degrees filling in-between—as typological predecessor of pentatony.

48. Audio: Song of a messenger Soruk Bollur from the olonkho Nyurgun Bootur. This comic character is grotesquely hyperactive and is often represented by a mode with four degrees (B2-F#3-B3-D#4) where intermediate degree keeps shifting between G, G#, A and B (Alekseyev, 1976, p. 234)—very much like Yasser's formula. http://chirb.it/dtCOEz

Scalability has nothing to do with poor pitch discrimination—its origin is functional: thus, Central African musicians discriminate differences of about 20 cents, while allowing certain degrees in a PS to be more flexible in tuning than others (Léothaud et al., 1997).

## Emmelic organization and oligotonal mode

Introduction of the leap/fill-up contour marks the transition point of ekmelic music to transform into emmelic. This contour calls for going over two degrees or more. An extra degree is needed to fill the leap with obvious gradual motion. The singer must coordinate four intervals. Such operation unavoidably reduces their elasticity. The task of filling causes the singer to estimate the interval of a leap in terms of *increments*.

Calculated leaping is remarkably different from khasmatonal type here—leaps proceed from one demarcated pitch zone to another. This task is harder than jumping from one margin of the compass to another, and demands focusing on operating the pitch parameter alone. Pitch breaks away from timbre, and follows its own route to rationalization. This is a gradual process, without a hard line: old Norwegian zithers feature fixed unison, 5th and octave frets, leaving other tones variable (Sevåg, 1974).

49. Audio: Underground Bootur, olonkho Kiun Djesiuyoldzhiut. Contrast in permanence of tuning between different degrees in a mode. http://chirb.it/1eEdJz

*Solo* “song-for-oneself” could not have emerged without following a publically available prototype. Such prototype had to be the *collective* singing of the entire community. A solo intonation can be crystallized into an idiom only when it is reproduced in more or less the same way by a substantial number of singers over an extended period of time. Call-response structure, common across so many cultures, builds the framework for testing which intonations are accessible and which are not. The chorus singers “live through” the emotional experience they sense in the soloist's part, and spontaneously unleash their individual melodic responses (Alekseyev, 1976, p. 164). Their clustering or perfect-matching polishes formulas adopted by soloists, and sets the ground for definition of “stable” vs. “unstable.” Each obtains its valence through procedure of question (unstable indefinite) and answer (stable definite), instilled by the responsorial format (Jordania, 2005).

50. Audio: Evenki sedye, Indigirka region “responsorial” song-dance. http://chirb.it/GEDEqq

Rare multipart singing amongst the ethnicities of Extreme North demonstrates how melodic intonation is carved through the collective experience of trial and error in making the melodic formula more expressive—supporting pitch-matching incentive with entrainment locomotion of the social dance.

51. Audio: Osuokhai, Vilyuy River region. “Stochastic” choral singing to the spontaneous dance. http://chirb.it/hwFLH4

The “tune,” molded by the collective effort, averages vocal abilities of all the participants, and reflects their shared representation of the “same” formula. Collective singing here works analogous to morphing of photographic images—by canceling out idiosyncratic features and keeping the average—generating the most attractive image (Langlois and Roggman, 1990). Attractiveness of averaging is not limited to faces, reflecting a wider bias for things familiar (Halberstadt, 2006). The origin of this lies in processing ease (Winkielman et al., 2006), specifically in processing fluency (Trujillo et al., 2014). Morphing of pitch and timbre seems to follow suit (Bruckert et al., 2010). Then, the averaged intonation becomes attractive enough to set a model for solo singing. Solo intonation is a replica of collective intonation (Skrebkov, 1973, p. 26).

Collective singing of sentence-based lyrics uniforms the pitches, coordinates intervals, and institutes compliance to unison/octave. Octave probably precedes the 5th in emmelic PSs. Not only the combination of high- and low-range voices produces vertical octave, but melodic octave is very suitable to khasmatonal music due to the convenience of octave-leaps in falsetto breaking (Heylen et al., 2002).

52. Audio: Night chant, Navajo. Falsetto voice represents Spirits, while regular voice—humans (Rhodes, 1949). The resultant octaves are strictly melodic, and do not bear formative modal function, http://bit.ly/1O68q4s

The very idea of using pitch rather than timbre for coordination in collective melody-making necessitates tuning—in the same way as choir singers are known to instinctively match the prosody of the choir leader (Skrebkov, 1973, p. 27)—be it within a group of singers, or a set of degrees within a PCS. Noteworthy, the word “intonation” is derived from adding “in” to “tone,” implying the process of bringing one's pitch in agreement with someone else's.

This process transforms the ekmelic mode into *oligotonal*^38^ —the next evolutionary stage. Practices of leaping-over-degree and singing-in-unison forged the distinction between tracing and displacing melodic intervals^39^, reported by Merriam (1964, p. 120) on African music across varieties of tempi—leading to discovery of three basic *absolute* intervals: 2nd, 3rd, and 4th.

53. Audio: Raven song, Kwakiutl Indians. Imitation of raven, fixed 2nd, 3rd, and 4th with two stable degrees, F and A, and other variable in pitch (Halpern, 1974). http://bit.ly/1GbqWnM

Stabilization of pitches activated gravity. Thompson (2004) calls this phenomenon “pitch bunching”—a strategy of music users to perceptually join musical tones into blocks of horizontal and vertical harmony. Listeners judge tones that are closer in pitch as more similar (Krumhansl, 1979), and expect proximal unstable tones to move toward stable ones (Bharucha, 1996). Such relations within the nucleus of a mode *compress* it by pushing the unstable tones closer to stable ones. Expressive tuning effectively puts a stop on the *centrifugal* tendencies in ekmelic music. Compressed nucleus does not allow marginal tones to “float” away in pitch (Presentation 2 in Supplementary Material).

Fixation of pitch directly causes generation of mode. Experiments show that in non-conventional music, the more frequently used tones are rated by listeners as better fitting (Cuddy, 1997). The frequency of occurrence of each of the fixed pitches in a song exceeds that of each of the variable pitches—promoting a sense of tonal ensemble between the fixed degrees.

As PSs center around tones that belong to the modal nucleus, the kernel of a song simplifies, compared to ekmelic mode: variant degrees disappear, leaving out few permanent degrees, more or less fixed at their pitch values. An oligotonal style makes a rather bare impression compared to a khasmatonal style (Presentation 3 in Supplementary Material).

Bareness stems from mechanical clarity of gravity: in order for a tone to increase in stability, its neighbor must proportionally reduce in stability.

54. Audio: Address to Altai Spirits. Prominence of vocal Ab3 causes G appear unstable, similarly, Eb3 of topshuur makes neighboring Cb3 and Gb3 appear unstable. http://chirb.it/dgK5CF

Greater gravity increases attraction of neighboring tones, shrinking the intervals—the *decentralized* ekmelic mode loses its *centrifugal* melodic inertia and transforms into a *centralized* emmelic mode, governed by a *centripetal* melodic force. This substantial increase in harmonicity of tonal organization comes as a compensation for the surge in “cognitive dissonance” in Aurignacian culture: conception of the first Lunar calendars, re-orientation of life-style according to cosmic rhythms must have induced psychological stress on tribe-members who had to reconcile different notions of time (day/night, summer/winter, solar/lunar) and space, raising the need for “cognitive consonance” of music (Frolov, 2003).

## Mesotonal and multitonal modes

The emmelic PS could contain three equal 2nds, raising the need to distinguish between them. This issue was addressed by absolutization of *complimenting* function: all adjacent tones comprised pairs of principal and supporting tones. The mode expanded at first to mesotonal (5–6 tones) and then to multitonal—perhaps by the mid-Magdalenian period, according to the 9-hole reindeer horn found in 1954 in Molodova-5 (Ivanova and Zeitlin, 1987, p. 58).

Every new addition to the ambitus obeyed the same rule (Mazel, 1952, p. 61): a tone added above/below a stable tone acquired a supporting role, whereas a tone added above/below the auxiliary tone became stable. The melodic movement in such a mode proceeded symmetrically, where every even tone supported every odd tone. In a hemitonic scale this inevitably produced *triadic* functionality: three odd tones (i.e., I–III–V) were similar in sharing a stable function, while three even tones (II–IV–VI) jointly carried a supporting function. The triad induction stimulated genesis of vertical harmony by parallel homosyllabic singing that paired pitches over one degree (Jordania, 2006, p. 33). Kubik (2010, p. 172) calls this “counter-note pattern.”

55. Audio: War Song, Côte d'Ivoire. Encouraging song for warriors, parallel 3rd “over a degree.” http://bit.ly/1Jlz6aA

Modal unity was the primary force that blended vertical intervals—vertical harmony sprang from horizontal harmony. Degrees that were afforded as part of a melody in an individual part were distributed to other singers' parts—so that each participant had to hear his partners in order to make his own part. Tonal unity of a mode provided tonal unity for harmonic intervals (Arom, 2004, p. 220).

56. Audio: Mbuti elephant-hunting song. The PS tones are distributed between multiple performers in vertical (harmony) as well as in horizontal (melody) planes. http://bit.ly/1zAv0Gp

Growing importance of pitch in tonal organization resulted in gradual decrease in timbral articulation. The process of “pitch reductionism” (Schneider, 2001) began: the listener centered on a particular quality of a tone and used it along with the pitch to “fine-tune” the mode—initially, timbre still played a big role in tuning the PS, but gradually pitch outweighed it, establishing the culture of “clean” vocal production (“bel canto”).

The first emmelic tuning system was most likely “*step equivalence*.” Maintaining the same intervallic increment to build up the ambitus is the most intuitive harmonic idea in instrument making (see Appendix II). This harmonization of tuning usually is all that is necessary in such a system: any combination of tones becomes “consonant.” Hence, melodic all-permissiveness transpires into harmonic all-permissiveness.

57. Audio: Ae ‘Au, panpipe band, Solomon Islands. Parallel 2nd in equidistant heptatony. Step equivalent music usually remains immune to the triad induction because the near-equidistant degrees resist tonal resolution and formation of functional relations. http://bit.ly/1HKy84X

Kiganda and Javan near-equidistant pentachord mode presents the model where equal compression is the primary organizational factor (Kubik, 2010, p. 259). Here, the idea of tonal unity becomes reduced to intervallic symmetry and proportionality (Léothaud et al., 1997)^40^.

Compressed ambitus and asymmetric IS are imperative for odd/even induction to occur. Since stable tones are more frequent than unstable tones (Krumhansl, 1990, p. 271), the harmonic 3rd, formed between I–III, acquires the function of stability—connoting pleasure (Bidelman and Grall, 2014). Accustomed to the I–III, listeners conceptualize 3rd as a harmonic consonant entity, projecting it on III-V. However, because V degree often takes opposing function toward I, III–V subordinates to I-III, allowing their alternation as well as combination. In the last case they make a triad I–III–V, which turns into a stable consonant *chord*.

58. Audio: Soloveikia moi, Old Believer's, Southern Russia. Differentiation between permanently tuned I, III and V—in contrast with timbral and pitch variability of other degrees. The I-III-V axis stands as the “melodized” chord. http://chirb.it/K74dEP

The even degrees also form 3rd and triads, which execute an unstable function. Subsequently, their vertical harmony inherits a “less consonant” status: if the odd degrees produce a major triad, the even degrees end up with a minor triad that is often perceived as less consonant (Krumhansl, 1987, p. 40). Cognitive opposition of even II–IV–VI and odd I–III–V triads ultimately establishes the centralized tonality by defaulting all tones in a PCS to the I–III–V triad.

59. Audio: Aqausiq (children song), Baffin Land. 5-tone arpeggio major triad as the axis of stability. Emphasis on stability probably corresponds to the message of affection characteristic for aqausiq (Nattiez, 1976). http://bit.ly/1HKt7hk

**Figure 2:**
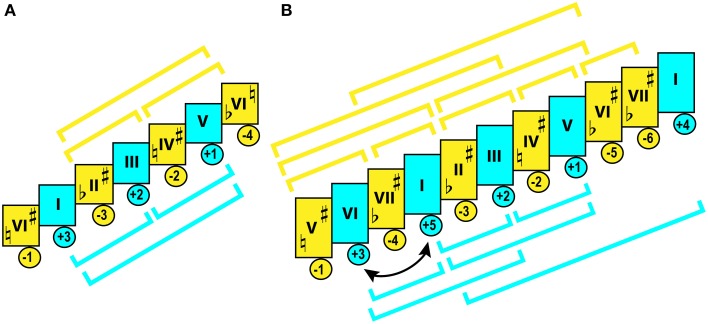
**Distribution of gravity in mesotonal and multitonal modes**. Blue represents stable, and yellow—unstable degrees. Negative values reflect instability, while positive values—stability of degrees (+5 more stable than +1, and −6 more unstable than −1). As apparent, multitonal mode generally exceeds mesotonal mode in tonal tension (it has maximal value of 6, whereas mesotonal has only 4). Roman numerals represent the order of degrees in a PCS, accepting the most stable degree as I. Sharps reflect the tendency of unstable degrees to rise in pitch toward neighboring stable degrees, flats—to lower degrees, and naturals—to stay neutral. Neutrality manifests a propensity of the unstable tone to occasionally act as an anchor in challenging the tonicity of I degree (I–IV, VI–I, or V–I). The brackets show typical functional grouping of degrees: yellow—unstable, blue—stable groups. **(A)** Hexachordal hemitonic mesotonal mode. The mode is defined by the balanced contrast of even and odd triads. Gravitational mutability can occur between I and III degrees, or alternatively, II and IV, if anchored. The VI degree is octave inequivalent: lower version is more stable. **(B)** Multitonal hemitonic octave equivalent mode. The black arrow indicates characteristic mutability between the stable low VI and I degrees. Notable is octave inequivalence of both, V and VI degrees. Upper VI and VII degrees bind in a group, either sharpened toward upper I, or flattened toward V degree. The mode is characterized by relative balance between the complex hierarchy of stable and unstable degrees.

At this point the genesis of modal chord production stops, since addition of one more 3rd I–III–V–VII introduces a dissonant 7th, depriving the odd degrees of tonicity (Figure 2A).

The even/odd principle of modal genesis is so powerful that it takes on “natural chromatic” modes that feature super-narrow intervals between the neighboring degrees. Thus, *bugarenjes* from Ćićarija are based on a row of 5–6 consecutive semitones (A-A#-B-C-C#-D, with E marking the climax phrasal points) (Marušić, 2007)—yet its 2-part singing proceeds as even+even and odd+odd degrees, despite the resultant harsh parallel 2nds.

60. Audio: Bugarenje. The principle of melodic motion in parallel interval “over a degree” prevails over concerns for harmonic consonance, which must be known to the performers, producing a raw of parallel major 2nds. https://www.youtube.com/watch?v=8rcgOiGJ6XU

Vertical harmonization can take unusual forms. Lithuanian Setu uses a decidedly symmetric organization of tones, separated by three sets of semitones that are each a tone-and-a-half apart^41^. The idea of “perfect” monointervallic consonances in 2-part polyphony matters more to Setu performers than variety of tonal functions (Ambrazevičius and Pärtlas, 2011).

61. Audio: Loikuslaul, reaping song, Setu. The mode is based on 5 tones: A, Bb, C#, D, and F, determined by the harmony of major 3rd. http://chirb.it/w2k7w7

Similar concept of monointervallic vertical harmony stands behind the Istrian 2-part singing in symmetric octatonic row (D-E-F-G-Ab-Bb-Cb) (Marušić, 2007).

62. Audio: Lovran je bili grad, Istro-Chroatian song. The mode is based on 6 tones: A, Bb, C, Db, Eb, Fb, determined by the vertical harmony of minor 3rd. http://chirb.it/1P9aLK

Such systems are built on “equivalence of 3rds” and are more functionally limited than asymmetric multitonal modes.

Triad induction theory compliments Shepard/Kameoka's consonance theory (Shepard, 2010) which demonstrates how division of octave into 12 semitones provides an optimal harmonic distribution. The simplest ratios of 5/4, 4/3, 3/2, 5/3, and 1/2 produce exactly I, III, IV, V, VI, and VIII=I heptatonic degrees. These “sweet spots” leave only two “valleys” for II and VII degrees to close the gaps and fill the octave with tones distributed diatonically. Hence, octave equivalence “initiates” with triadic genesis—heptatonic *horizontal* harmonization *vertical* harmonization (Presentation 4 in Supplementary Material).

Proof of this can be seen in the fact that consonant vertical intervals are produced by the relation of stable tones – which allow no dissonant vertical intervals (Teplov, 1947, 167). Non-musicians easily categorize intervals extracted from popular songs for which they are likely to have “melodic templates” (Smith et al., 1994)—but not when vertical intervals are presented in isolation. Melodic intervals are extracted from familiar intonations – and not mechanically “calculated” by estimation of intervallic distances (Teplov, 1947, 167).

Horizontal harmony is known to be capable of equalizing the IS. The intonation of a descending minor 3rd often forges the ambitus for strictly dichordal (2-tone only) solo songs, quite common for many cultures (Alekseyev, 1986, p. 118). Harmonic intervals in modes with equalized IS are directly inferred from melodic intervals, in the process of collective singing: slight desynchronization between parts would reveal a harmonic interval. The same applies to non-equalized IS: Beliayev considers 2-part polyphony of Russian folk music to originate from the variational deviation from singing the same tune (Beliaev, 1959).

Vertical octave and 5th are very high in fusion, and therefore produce weak relations between their tones (Huron, 2001). In opposite, melodic octave and 5th are very strong. This dissimilarity makes the inference of vertical octave and 5th from their respective horizontal versions highly unlikely. Early forms of polyphony usually evolve from the most common melodic intervals: unison, 2nd, 3rd, and 4th. Polyphony, based on octave and 5th, is likely to belong to later stages of tonal development. Computer recognition of tonal music discloses that wide vertical and horizontal intervals have competing relations (Cambouropoulos, 2008): high vertical fusion transpires into low melodic coherence.

The I–III–V are distinguished not by their fusibility but by *permanence* of stable function throughout the song (Mazel, 1952, p. 62)—in contrast to other tones that keep changing their functions under the influence of melodic context (rhythm, dynamics, articulation). The opposition of stable permanence and unstable volatility eventually finalizes the diatonic 7-tone mode scheme: unstable tones surround the stable tones (VII–II–IV–VI around I–III–V). The triadic principle inevitably forces *octave equivalence*—otherwise the lower VII degree would be unstable, while the upper VII—stable. Octave equivalence of I and VII degrees shapes the diatony. The upper register is marked by succession of unstable VI–VII, which perceptually marks I as the most stable in the mode.

Technically, *implementation of octave equivalence* to forge a multitonal mode *necessarily engages equivalence of 4th and 5th*. The modal construction requires junction of two tetrachords in order for the melody to be able to fluently run from one degree to its octave equivalent by the ladder of designated intervallic values. And the melodic bi-tetrachordal space has to be harmonically comprehended as a sum of the pentachord-based 5th (Beliayev, 1990, p. 290) and the tetrachord on top of it, realized as the 4th that is inverted from the pentachordal 5th (C-D-E-F-G + G-A-B-C). Thus, the pentachordal model of odd/even stable/unstable *alternation*, and the tetrachordal model of *enclosure* of unstable pair within the stable pair must both be integrated together to generate modally functional octave equivalence.

Chronologically, equivalence of 4th follows the step equivalence, precedes equivalence of 5th and most likely concurs with equivalence of 3rd as an alternative method of unifying modal steps into larger tonal subsets. The borderline is that tetrachordal organization tends to anchor one/both of the tetrachord's marginal tones while treating the middle tones as unstable. Equivalence of 3rd, on the other hand, favors even/odd functionality—paving the road toward pentachordal organization and equivalence of 5th. Therefore, equivalence of 4th is more typical for modes of mostly monodic cultures, whereas equivalence of 3rd and 5th—for those of polyphonic/heterophonic ones.

Modal octave equivalence follows the equivalence of 4th and 3rd and concurs with the equivalence of 5th. Octave equivalence has a washed-out time-frame, because it is realized gradually over time for different modal degrees: at first for the central anchor, then for complimentary anchors—and only afterwards for unstable tones, based on their modal importance. Complete octave equivalence characterizes professional music culture in civilizations that developed literacy (see Part-2).

Octave equivalence marks the transition from additive to divisive tuning methods: referential octave is initially built by adding “standardized” steps—and often is flexible, affording diminution or augmentation (Léothaud et al., 1997). When ambitus grows enough, causing many tones to form pair relations, music-users learn to recognize “tints” in coloration of the same PC across registers (Kolinski, 1978). Then octave becomes modally formative, and transforms from the sum of the reference intervals into an IC, eventually leading to invention of temperament.

However, the octave equivalence affects different degrees differently, depending on their melodic function. Thus, the upper-VI degree is considerably more unstable than the lower-VI degree which often acquires the function of relative “tonic”—in Western musicology this phenomenon is known as “double-tonic” (Gelbart, 2013). Odd degrees vary their tonal function between octaves as well. The upper-V degree is more stable than the lower-V, because the lower-V often serves as the infrafix “leading tone” to the I degree (Figure 2B).

Octave disparities put in place modal *mutability* (Bakulina, 2014)—the tendency of tones to change in gravity and magnetism as the melodic motion proceeds from one register to another. The most common form of mutability is alternation of centripetal function between the I and low-VI degrees: lower register spectrally increases the gravity of the lower stable tones as compared to the upper tones, while maintaining the odd/even ranking. Shift of gravity I-VI only slightly affects the functional inclinations of other degrees in a PS (i.e., IV remains unstable)—preserving the unity of the mode.

63. Audio: Ocarina solo, Bulgaria. Octave equivalent PS with two variable in pitch degrees: E-F#-G#(G)-A-B-C#-D#(D)-E with alternation of gravity between “tonic” I and lower infrafix VI A/F#. http://bit.ly/1Ga3LYa

## Pentatony vs. heptatony

Just as hemitonic organization logically leads to genesis of octave-equivalent heptatonic modes, so does anhemitonic pentatony. A trichord C-D-F forms its nucleus (Beliayev, 1990, p. 301).

64. Audio: Kyzyl Taiga, Tuva. Beliayev's trichord lies at the base of the PS: B-C#-E-F#-G#. http://bit.ly/1F5N69o

The early pentachord-based mesotonal modes are often mislabeled as “pentatonic.” The principal distinction is the *ambitus* of the song and *functionality* of the degrees within the mode.

65. Audio: Haida play song. Non-octave equivalent pentachord based mode: B-E-F#-G#-B-C#. Upper and lower Bs carry different modal functions. http://bit.ly/1eUgohm

Mesotonal degrees are register specific: when the ambitus exceeds the octave, the upper intervals differ from the lower ones (Fernando-Marandola, 2007).

**Figure 3:**
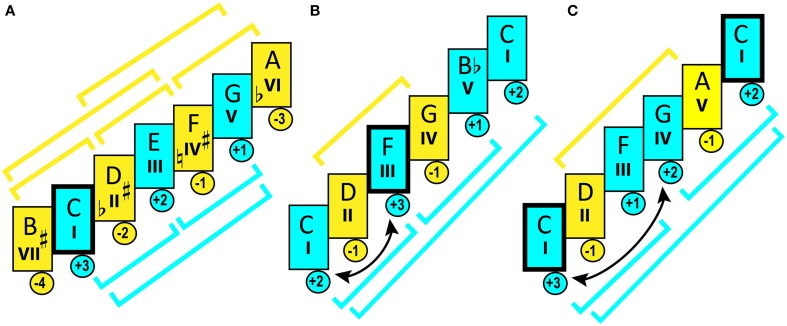
**Distribution of gravity in early pentatonic and hemitonic modes**. Yellow color indicates unstable, while blue—stable degrees. The intervallic distances between the degrees are reflected by vertical increments between the rectangles representing the degrees (as in an uneven staircase where some steps are taller than others). Thick rectangle marks the gravitational center. The I degree is determined by the lowest position in a base of a stable tetrachord or pentachord. **(A)** Heptatonic mode. Sharps and flats show strong attraction of unstable degrees to stable degrees (except the neutral IV degree). Gravity here is hierarchical: the relation of 3rds (shown by smaller brackets) is integrated into a triadic relation (as shown by larger brackets). The stable triad *opposes* both unstable triads. Diverse gradations in gravity (from −4 to +3) between the degrees. C is strongly marked as “tonic.” Unstable degrees have finer gradations than stable degrees, and reach high values of tension. **(B)** Conjunct (older) pentatonic mode. Flatness of gravitational hierarchy between the unstable degrees. F and C alternate as centers of gravity, with F prevailing. Stable 4th-chord “triad” (C-F-Bb) entraps unstable 4th dyad (D-G). Unstable degrees have low tension and no gradations. **(C)** Disjunct (newer) pentatonic mode. C and G alternate as centers of gravity, with C prevailing. Stable 4th–chord “triad inversion” (C-F-G) is offset by unstable 5th dyad (D-A) that has a stronger pull than the dyad in **(B)** because of greater harmonicity of 5th as opposed to 4th.

The earliest forms of pentatony seem to originate from conjunct (Sachs, 1962, p. 159) and then—disjunct extrapolations of the basic trichord: C-D-F+G-A-C and C-D-F+F-G-Bb (Beliayev, 1990, p. 301). Both versions rely on 4th as the primary mode-building interval, and 5th as secondary (C-G in disjunct, and F-C in conjunct trichord)^42^. From perceptual perspective, this means that early pentatonic modes lean on the lowest tone of the basic trichords and its octave equivalence: C-F-C or C-G-C. These tones define the stability axis for the two earliest pentatonic modes. They also implement the even/odd principle, discovered during the earlier mesotonal stage, but in a new way. The cycle odd-even-odd-even-odd is finalized by succession of two *stable* degrees (Figures 3B,C)—unlike the two *unstable* degrees in hemitonic mode (Figure 2B).

This difference is responsible for the strong gravity in heptatonic mode, contrary to the weaker pentatonic gravity (Figure 3A). Succession of two adjacent unstable degrees increases tension, calling for resolution, which charges the closest stable tone I, a semitone apart, with the greatest gravitational value. Its priority is further amplified by the hierarchical relations: stable I–III and I–V, as well as unstable II–I and IV–I, all stress I. The combination of two adjacent stable degrees in pentatonic mode executes an opposite effect: it creates competition between two anchors V–I, thereby reducing attraction of the closest unstable tones.

If the gravity values for stable degrees are comparable between the pentatonic and hemitonic schemes, then the magnetism values for the unstable degrees are strikingly low in pentatonic genera. Unstable degrees do not form hierarchic relations in a mode—presenting little “resistance” to the gravity of stable tones. Subsequently, pentatonic modes are distinguished by little tension, which manifests itself as “dispersed,” weakened gravity—despite the presence of some hierarchical relations between the stable degrees. This goes to confirm the greater importance of magnetism than gravity in perception of tension (Larson and Vanhandel, 2005).

Unfortunately, the issue of tonicity in pentatonic mode has slipped away from the attention of cognitive scientists. The only cross-cultural experimental study tested perception of Korean traditional court music (Lantz et al., 2014), and found that pentatonic tonicity was weaker than heptatonic^43^. A sequel analytical study (Nam, 1998) identified presence of tonal organization in pentatony—generally confirming Beliayev's “disjunct model.” Wilbanks and Pate (1979) reported that their listeners distinguished between melodies based on I- vs. V-degree pentatony, but their subjects were all Westerners.

Pentatonic ISs manifest much greater harmonicity than heptatonic ISs (Gill and Purves, 2009), somewhat obscuring the tonic. Most ethnomusicological field studies of pentatonic music do not provide clear definition of tonicity as understood by subjects of the study—which can range from a pure “reference tone” for tuning (Nguyen, 1986) to a specific tone in the ambitus, marked by 3rd or 4th (Nettl, 1953). Some researchers assume that “tonic” is obvious through mere calculation of incidence of all the tones in a song, and its finalis (McLean, 1991).

Authentic music theories of cultures based on pentatonic systems emphasize 4th and 5th as formative elements in PCS, and define tonic in their terms. Ethnomusicologists question how practical are such claims (Karpati, 1980), and propose the intervallic contrast between major 2nd and minor 3rd as the principal perceptual method of tonal orientation in anhemitonic music (Reinhard, 1958). Justification of such approach is provided by Maceda in his overview of tuning practices in Southeast Asia (Maceda, 1990).

A rational scheme for generalization of tonicity in pentatonics was proposed by Beliayev, inferred from the tuning design of Eurasian folkloric instruments—correlated with morphological analysis of music performed on these instruments. Beliayev regarded the tonal essence of pentatonics to lie in unequal division of 4th in two, and expanding the mode by reproducing this division a register higher^44^. Beliayev (1990, p. 305) held that modes grow from bottom to top, gravitating toward the lowest tone, standardized by tuning—from which the newer modes are formed by the ascending transposition of the mode. Tuning of the accompanying instruments restricts creativity of singers, directing them in leaning on instrumental anchor tones (358). Hence, C and F (Figure 3B), as marginal tones in the lowest 4th in a *conjunct* PS, compete for tonicity. However, F is dubbed as the end and beginning points in two trichords, receiving perceptual advantage over C. *Disjunct* mode (Figure 3C) replicates the bottom trichord from G, thereby instigating competition between trichords and their gravitational alternation. Then, proximity of the upper octave-equivalent C gives an edge to C. *Conjunct* mode also implies alternation—both Cs challenge the superiority of F.

Further confusion comes from the IS. Contrary to *linear* heptatonic melody, melodic motion in pentatony is *cyclic*: balance of “continuity-dispersion-continuity” (Zemtsovsky, 1998). Horizontal consonance of 2nd is offset by dissonance of 3rd and resolved back into 2nd. This symmetric scheme is reproduced across different registers, generating the characteristic pentatonic “terrace” of overlapping trichords: the relationships “C-D-F” and “D-F-G” are rebuilt from different degrees. The resulting modularity of pentatonic melodies reduces gravity, evident in allowance of extreme jumps (11th)—even in village folk-songs (van Oost, 1912, p. 167).

66. Audio: Sunzhidmaa, lyrical Bogino Duu, Mongolia, accompanied with shanza. Terrace-style melody effectively disperses tension and leaves little space here for genesis of hierarchical relations between unstable degrees. http://bit.ly/1JYrXxT

This trait makes heptatony and pentatony disagree in their melodic “philosophy.” Pentatony cultivates conservation and rigidity of functions, whereas heptatony champions diversity of functions and capacity for intense tonal development (Alekseyev, 1986, p. 174). Both systems should be regarded as competing methods of music thinking: i.e., adherence of Berber (native population of North Africa) folklore to pentatony (von Hornbostel, 1975, p. 363) despite over 1000 years of strictly heptatonic Arabic presence.

It is the correspondence of even degrees with vertical dissonances and odd degrees with consonances that empowers tonal dynamism in heptatony. In pentatony, even and odd degrees behave differently—generating “4th-chord axis” (odd C-F-Bb vs. even D-G). Poorer harmonicity of “4th-chords” fails to bind odd degrees into an acoustic conglomerate where as a result, even degrees don't develop clear functionality: they don't develop a tendency to go to a particular tone. Also, C-F-Bb presents no harmonic contrast to D-G-C, whereas heptatonic major C-E-G clearly contrasts minor D-F-A. Finally, the pentatonic ICS balances harmonic dissonance and consonance between the PCS degrees: in conjunct mode, II = 2nd and IV = 5th (from I), vs. I = unison, III = 4th and V = 7th.

Lack of contrast in odd/even degrees provides little incentive for music-makers to experiment with condensing tension. Moreover, pentatonic melody receives its gravitational “thunderbolt” in jaggedness of pentatonic “steps.” Frequent rupture between the melodically consonant 2nd and the dissonant 3rd “steps,” vents out much of the tonal tension, depriving the composer of means to build tension by accumulating a series of unstable tones, and delaying resolution. Unevenness of steps prevents melodic intervals from turning into “capacitors” of tension. Pentatonic melody is bound to glide from one “terrace” to another across the compass of a composition—no matter how wide the leaps are, and how fast is the tempo.

67. Audio: Two little ducks, lyrical urtyn duu, Mongolia. Little tonal gravity allows for great inertia in the melodic line. http://bit.ly/1JYtkNb

Opposition of pentatonic and heptatonic orders is strongest in Occidental vs. Oriental music systems. Western musicians pursued in hemitonic direction despite their awareness of pentatony at least from the early Middle Ages (Szabolcsi, 1943). From the mid-eighteenth century, pentatonic modality received attention in Western musical discourse (Day-O'Connell, 2007, p. 84), but made little impression on composers, even in Ireland and Hungary, where pentatony was prominent in folklore. In a similar vein, ancient Chinese theorists knew about the heptatonic scale as the “seven beginnings,” but chose to limit themselves to the first five 5th in their circle, in observance of “five elements” dialectics (Daniélou, 1995, p. 33). Evidently, Neolithic Chinese used hemitonic modes (see Appendix II).

## Summary: pitch organization as a general organizational scheme

There was a time when music was organized not by pitch, but by timbre (see Appendix III for the timeline of tonal organization). From this follows that pitch is a cultural product. As such, its organization can reveal important information about the culture that produced it.

Absence of concise control over pitch provides information about the mental methodology behind it, too. Navigation through pre-modal melody occurs by mapping a specific musical tone at sequential points of a contour, (start, end, peak) in relation to the singer's compass. This mapping anatomically corresponds to memorization of places of tension in the vocal folds, in successive order. Such pitch control correlates the entire vocal compass to the entire contour, and corresponds to the *syncretic* method of thinking that does not distinguish object from subject, cause from consequence, form from content. This method involves little interpretation, relying mostly on instincts in response to sensory stimulation and memorization of a vocal call as a “snapshot”—uneditable entity (see Appendix IV for the summary of structural features of tonal organization).

Khasmatonal organization presents an important upgrade: it introduces the categorization between *abrupt* vs. *gradual* change. The singer divides his compass into a few registers and defines pitch in reference to them. Individuals substantially vary in their timbral capacities, which is why identification of a particular tune by its timbral contour is hardly possible. The pitch parameter offers significant advantages for transmission of melody, providing markers that are easier to reproduce and recognize. Representing khasmatonal melody in terms of continuity within a particular pitch register vs. leap to another register brings about conscious control over pitch, albeit elemental. Khasmatony corresponds to a mindset capable of centering on a particular aspect, and tracking changes in relation to it. Khasmatonal melodies cultivate such centering and promote cultural activities that mark “extra-ordinary” things out of ordinary ones—such as coloring stones during Middle Pleistocene.

Organizing power of khasmatonal music is evident in motherese. Mothers communicate to their babies through a recitative-style speech, alternating it with vocables, sung out on a descending gliding 3rd, which is infants' preferential intonation (Reigado et al., 2011)—in contrast to the exaggerated cadential leaps. This vocabulary is learned by 0–2 years old children who pick it up from their mothers (van Puyvelde, 2010), starting by gliding indefinite pitches, and proceeding to definitely tuned 2nds, 3rds, and larger intervals (Hargreaves, 1986, 70) - according to the contour schema theory (Davidson, 1994) and other researchers (McKernon, 1979; Radynova et al., 1994; Rutkowski, 1997; Welch, 2006).

68. Audio: Lithuanian lullaby. Khasmatonal style in motherese. http://chirb.it/AKcGJG

Learning to track general changes in pitch helps in other cognitive tasks (i.e., visual tracking of motion) (Huddleston et al., 2008). Motherese constitutes perhaps the oldest surviving remnant of khasmatonal past that encapsulates multimodal interaction in a ritualized form (Dissanayake, 2004) - strongly representative of syncretic nature of khasmatonal music.

“Discovery” of pitch as an autonomous expressive parameter was groundbreaking for humanity. Essentially, pitch is the *perceived quantity of harmonization*: a grade of periodic vibration detected in a raw sound as a function of frequency, time, and timbre (Yost, 2009). The extent of pitch discrimination indicates sensitivity to the proportionality in the spectral content. Proportionality is a unique marker of music—even individuals with congenital amusia reproduce pitches more accurately in singing than in speaking (Liu et al., 2013).

Pitch is exceptional in its capacity to simultaneously convey large number of signals, in perceptually easy (McDermott et al., 2010a) and semi-automatic manner (Bidelman and Grall, 2014). Combined with the evolutionary importance of the auditory system in urgent and information-intensive communication, outlined by Jerison (1973), high throughput of pitch encoding provides unparalleled advantages for the brain. Hearing is the only distant sense that has an urgent effect during sleep (Wilson, 2000, p. 235)—its input is the most direct in delivering information about the environment. Unlike vision, hearing makes environmental information readily available for the brain right from birth. Neonates are found to have all the main auditory functionality that characterizes adults' hearing in place (Bendixen et al., 2015). This includes discrimination between melodic intervals (Stefanics et al., 2009). Moreover, the ERP response to deviant tones with variation in resonance demonstrates that the neonate auditory system represents pitch separately from timbre (Háden et al., 2009). The fetus can discriminate between the frequencies at 27–35 weeks (Litovsky, 2015).

Early pitch extraction should be seen as a genetic adaptation, based on synesthetic connection between pitch and size (Marks, 1978, p. 53). The dimensions of height and volume are cross-modally mapped to the perception of pitch in 4-month old infants (Dolscheid et al., 2014). Neonates detect changes in the size of a musical instrument by ear (Vestergaard et al., 2009). The pitch-size connection must have evolved due to its selective advantage for distant detection of larger predators. Now, after civilization has removed dangers of being preyed, the principal advantage of pitch medium is conditioning the neural paths for optimal execution under ultra-heavy loads of data processing. The specialty of the pitch domain can be observed in the exception of quite widespread phenomenon of “perfect ear” from the “seven plus or minus two” rule formulated by George Miller (1994) and recently updated to “four plus/minus one” (Mathy and Feldman, 2012). “Absolute pitch” implies instant access to at least 12 chunks^45^.

Poor pitch control in khasmatonal music corresponded to little workloads of information: limited verbal development did not allow much data to be cognized, requiring little compensation from harmonizing power of music. Scalable pitch in ekmelic mode corresponded to increased verbal skills that put pressure on music users to increase its *pitch strength* (pitch saliency)—the relative strength of the perceived frequency component of the complex sound as opposed to the overall spectral content of that sound (Shrivastav et al., 2012). Intonation progressed from psychophysiological and organophonic to *resonant* type—based on pronounced sustainment of clearly pitched tones (Sheikin, 2002, p. 30). Reduction in timbral mutability, and increase in periodic spectral content established pitch as the *standard* for melodic reference across all musical instruments and vocals. Together with pitch rose musical mode, gaining control over melodic organization across varieties of genres. Harmonicity of tone led to harmonicity of mode.

Pitch contour has been a definitive force in modal genesis. Melodic contours and intervals appear to be processed by different neural domains (Liégeois-Chauval et al., 1998). Perceptually, contours have higher plasticity than intervals (Fancourt et al., 2013), and form the base for processing not only melody, but also loudness and timbre (McDermott et al., 2008). Furthermore, it is the melodic contour that seems to supply the model for timbre/loudness contours (Graves et al., 2014). The contrast between ascending and descending contours in ekmelic music defined the transition points in the melodic line, stabilizing them in pitch. The notion of interval emerged to facilitate orientation and to secure stereotypicity in communication of contours. Along with four basic intervals came four scalable degrees. Together, they enabled *conservation* of pitch, albeit far from perfect. Orientation in pitch was only possible in reference to an ongoing smooth sinusoid cycling of the same melodic formula.

One consequence of uniformity of contour in the absence of a fixed IS is the multicultural distribution of melodic formulas. Sheikin reports that hunting and onomatopoeic songs reveal amazing similarity between remote communities—suggesting that there must be some underlying cognitive mechanisms that influence melodic thinking of people who subscribe to the same principles of tonal organization (Sheikin, 2002, p. 234). Zemtsovsky (2001) holds the idea that “manner of thought is a category that can be historically determined”—and music is only one of the implementations of the historically determined world view models.

Further linguistic advance affected lyrics: from vocables, texts evolved into rhymed sentences. Conversion of metric-markers into pitch-markers built the frame for the oligotonal mode. Fixed degrees made intervals absolute, establishing the ABC's for the faithful replication of melody. Trial-and-error reproduction of melodic intervals in collective singing highlighted the match/non-match vertical relationship. Harmonization encompassed vertical and horizontal dimensions, establishing the *mechanical* principle of functional correlation between the neighboring degrees in the PS: any increase in instability of a degree ought to be matched by equal increase of stability of an adjacent degree. The *mechanical centripetal* gravity supplanted the *centrifugal* gravity of earlier systems. Pairing degrees by tightening the interval between proximal stable and unstable tones compressed the ambitus, disallowing the marginal tones to stretch wider apart from the center. Oligotonal nucleus became simplistic yet *rational*.

Mesotonal mode applied the “proximal contrast” principle over the entire nucleus, compressing the distances between the stable/unstable pairs. This is where evolution bifurcated into pentatonic and heptatonic directions. Heptatonic scheme *harmonized* the relationship of one pair to another (see Presentation 4), whereas the pentatonic scheme—did not. Difference in harmonization strategies gave birth to contrasting musical philosophies.

Pentatonic mode divided the PS in two trichords, where the marginal tones were stable, while the medial tone—unstable. Unlike division in pairs, such method did not promote hierarchical relations between unstable tones. Trichord music lacked in tension and had a homogenizing effect on its users. Opposition of both types of music grew as their respective instrumental traditions established dedicated methods of tuning (see Appendix II). Hemitonic music relied on ternary division of 4th (three 2nd), while anhemitonic music—on binary unequal division of 4th (3rd+2nd). Ternary division made melody incremental and proportional, enabling accumulation of tension as long as it was counterbalanced by corresponding amount of release of tension. Binary division of 4th, due to gaps, did not allow for cumulative succession of unstable degrees.

Perhaps, cultural stereotype of opposing Western *dynamic* and Eastern *contemplative* worldviews reflects opposition of *hemitonic* and *pentatonic* orders. If to consider the musical mode “the DNA of musical cultures” (Jordania, 2006, p. 101), then distribution of anhemitonic pentatony along the geographic areas populated by mongoloid ethnicities has been long known (Sachs, 1962, p. 162). Jordania (158) distinguishes between hemitonic Middle Eastern group of Old High Cultures, stretching from North Africa to North India—except a few pentatonic oases^46^, and pentatonic East Asian group, encompassing China and Southeast Asia. Perhaps hemitony and pentatony present musical genomes corresponding to genomic diversion between Western Eurasians and East Asians 38,700–36,200 years ago (Seguin-Orlando et al., 2014).

The “gapped” nature of pentatony might have originated in the phonetic contrasts of tonal languages. Kubik (1985) reports that several African music cultures have music systems built upon patterns of their tonal languages. Many East Asian, Tibetan, Central, African, and Amerindian ethnicities combine use of tonal languages and pentatonic music. One study found that in Chinese, Thai, and Vietnamese music and speech, the pitch intervals are wider and more contrasting in direction, as opposed to American, German, and French (Han et al., 2011). Possibly, articulating tonal speech promotes wider intervallic spacing in pentatony.

It could be noted that many music cultures adhere to hemitony—if they prioritize innovation—or to pentatony—if they prioritize conservation^47^. Change in priority corresponds to change in tonal organization: switch from pentatony to heptatony accompanied the rise of numerous great civilizations^48^. Rejection of pentatonic order is most pronounced in Western classical tradition, which has been adopted by world music cultures along with the Western lifestyle. Its antipode is Chinese music. Chinese civilization championed outstanding achievements during the time when hemitonic music was cultivated there: two hemitonic flutes dated 6600 BC from Jiahu (Zhang et al., 2004) concurred with domestication of rice (Liu et al., 2007) and beginnings of literacy (Li et al., 2003). About the second century BC, Chinese music theory started restricting music to pentatony, eventually suppressing the heptatonic genres—in parallel, technological innovation decelerated after the Yuan dynasty (Adshead, 2004)^49^. As Confucianism, supported by the state starting from Han period, was gaining increasingly more control over state policies, the heptatonic scales became associated with suyue—vulgar entertainment—and foreign influences, incompatible with Confucian aesthetic principles (Furniss, 2009), establishing the view that heptatony is alien, less harmonious and morally inferior to pentatony.

Noteworthy is that the time when “open doors” policy eased access to Western music, raising great interest in Chinese population (Kraus, 1989), coincides with the growth in creativity and giftedness in younger Chinese generations (Qian, 2008)^50^. Understanding of creativity in traditional Chinese thought differed from Western understanding: creativity was related to *rediscovery* of the nature of things, an inspired imitation of nature, unassociated with invention or celebration of an individual's accomplishment (Rudowicz, 2004). In contrast, post-1990s polls of Chinese university students demonstrate that creativity is understood as generating an idea that never existed before, useful for society (Yue and Rudowicz, 2002)—in tune with Western understanding.

Western and Eastern traditions utilize different norms. A Westerner is eager to distinguish himself from others; An Easterner seeks to merge with others in social harmony. These standards are learned from childhood, without much awareness (Morris and Leung, 2010). Recently, their origin was explained by differences in processing alphabetic vs. phonographic literacy (Hannas, 2013), but the underlying cause could be music^51^. First writing systems emerged around 3000 BC in Mesopotamia. First record of pentatony comes from 7000 BC China (see Appendix II). Since literacy follows the establishment of oral language, and the origin of oral language concurs with the origin of music, the tonal organization of music must precede literacy, and the choice for specific characters to graphically represent the sound of speech is made under the influence of cognitive schemes developed for perception of music. Conservatism and holisticism in logographic thinking correspond to conservatism and holistic harmonicity of pentatonic order (where the entire PS is harmonized as a whole to make any combination of tones in vertical or horizontal pleasant to the ear), whereas radicalism in alphabetic thinking corresponds to radicalism of heptatonic order (where the music user has to elaborate a unique tension map for each music work).

Heptatonic tones are distinguished from one another by hierarchic tension. Pentatonic tones are blended by non-hierarchic minimized tension. Emotional component of the instinct to comply to the “norm” can be primed by the emotional response to fluctuations in tension experienced upon auditioning the music (Krumhansl, 2002). The Westerner likes resolution that brings relaxation, hence developing a need for tension—which projects into an instinct to fulfill personal aspirations, find differences between oneself and others, and “resolve” them by a dedicated action. The Easterner likes relaxation, and develops aversion to tension—which translates into an instinct to avoid social confrontation by moderating one's aspirations.

This generic opposition has exceptions. In India, anhemitonic modes during the Vedic Age evolved in a hemitonic system (Gauldin, 1983), but during the Sangam period, pentatonic modes were rediscovered (Rowell, 2000). This integration possibly reflected greater importance given to meditation and harmony. Plurality of choice between hemitonic and anhemitonic typology, selected as needed for a genre application, is found in East Asian communities (Maceda, 1990). This compromise trend is also evident in modern globalization, marked by the raise in popularity of meditation amongst Western population (Haynes, 2004) on one hand, and high investments of governments of the most developed East-Asian countries into creativity/innovation cultural programs (Xiang and Walker, 2014), on the other hand.

Hybridization of pentatonic and heptatonic tonal principles in popular Western music compliments this trend. Blues served as the foundation for a range of styles where heptatonic order remained the driving force behind the vertical harmony, while pentatonic order governed the horizontal harmony. Cazden (1971) regards Anglo-American folk tradition as a demonstration of persistence of pentatonic order (stemming from amalgam of older village cultures of Ireland/Scotland with American Indian and West African musical traditions) against the pressure from the chromatic organization of Western classical music—where *hexatony* acts as a compromise.

Most of today's world popular music is based on pentatonic melodic line that receives functional harmonization. Pentatonic melody there serves to disperse the tension generated by intense rhythmic syncopation, dissonant chords, and unstable harmonic progressions in the accompaniment—capitalizing on the decentralizing power of pentatony, and moderating harmonic development in order to keep the music pleasant to ear (Biamonte, 2010).

Music seems to set the direction for behavioral attitudes that motivate the choice for a specific strategy in approaching a mental task^52^. Commonality of music and relative ease of its processing—as compared to the other sensory modalities and speech—make music the prime choice for elaboration of optimal schemes of cognition, suitable for a particular environment, and to reinforce the cultural reproduction of this optimal scheme across a given community of music users, securing uniformity in their worldview, and building their social cohesion^53^.

### Conflict of interest statement

The author declares that the research was conducted in the absence of any commercial or financial relationships that could be construed as a potential conflict of interest.

## Footnotes

^1^Thus, music based on contrast between consonance/dissonance must be derivative from music based on contrast between variable-in-pitch and fixed-in-pitch tones, since in order to produce consonance one has to be able to generate pitched sounds.

^2^Yet another prospect for dating tonal organization is tying it to a particular ethnic group and analyzing its genetic affinities—defining “musical genomes” (Grauer, 2007).

^3^There is a need for a music theory that would “place each musical phenomenon in an ecological context motivated by evolutionary considerations” (Wiggins et al., 2010).

^4^Except the heptatonic 2nd on VI–VII degrees, where VI usually is more stable.

^5^Melodic weakness might be responsible for the asymmetry in detection of melodic intervals reported by Schellenberg and Trehub (1996): weaker relation of tones in well-fusing intervals make them appear “vague” after intervals with strong relation of tones. Perhaps, this exposes contextual distance of melodic intervals, analogous to contextual distance of tones (Krumhansl and Cuddy, 2010): progression from a higher tension interval to a lower tension interval appears psychologically more distant than its reverse—greater distance transpires in poorer detection.

^6^Non-musicians show reduced frequency discrimination at interval differences below 125 cents (Zarate et al., 2012), which promotes perception and production of major as opposed to minor 2nd in folk-based music cultures (Kolinski, 1967).

^7^Melodic octave seems to operate by a different set of principles than that of harmonic octave (Bonnard et al., 2012): in vertical octave ear is sensitive to the slightest mistuning, whereas in horizontal octave, it is surprisingly tolerant.

^8^In the thirteenth century music theory, 3rd was regarded as imperfect consonance, whereas 6th—imperfect dissonance (Crocker, 1997).

^9^There is converging evidence of strong multi-sensory interactions between physical movement and musical rhythm (Trainor, 2007): “every musical sound has the capacity to specify some kind of motion (or its opposite—stasis)” (Clarke, 2005). Recent discoveries point to the vestibular system as a mediating agency in entraining and disambiguating of musical rhythm (Trainor et al., 2009).

^10^Already, von Hornbostel (1928) warned that *mode* should not be mistaken for *scale* : the same set G-A-B-C-D-E can act as a hexatonic G-major or A-minor. The distinction is possible only upon parsing the melody into phrases and examining the melodic function of the tones.

^11^The contribution of integrative function of mode to processing of pitch is best illustrated in the famous experiments by Attneave and Olson (1971). It is the long-term memorization of IS that is responsible for operational ease—as opposed to helplessness-in handling exactly the same task at the absence of tonal unity (Rakowski, 2009).

^12^The fact that listeners are sensitive to the tonal unity of a mode (Brattico et al., 2006), and map specific melodic progressions against their short-term memory of auditioned PS, correlating the results with the long-term memory of the known PCSs (Bigand et al., 2003)—was demonstrated by Rahn et al. (2004).

^13^Prominence of Asafyev amongst the communist officials made it easy to discard his theory as “Marxist propaganda.” He indeed had serious inconsistencies in his publications, and his style of research was more of a creative artist rather than a scientist (McQuere, 1983). But the “intonation theory” was elaborated by a cohort of specialists in numerous disciplines rather than by Asafyev alone (Lukyanov, 1977).

^14^Asafyev considered himself Yavorsky’s pupil (Konson, 2010).

^15^Listening to a series of 3-member subsets of pitches with the purpose to figure out the tonic revealed that the tritone-based 3-tone intonations were found to be most indicative of the position of tonic, in agreement with Asafyev/Yavorsky’s theories (Protopopov, 1930).

^16^The fact that adding two tetrachords together would make an octave was a coincidence, and not a principle of Ancient Greek music-making: 5th was the largest interval considered in melodic composition (Kholopov, 2006, p. 64). Octave species were byproducts of joining the tetrachords together within a key—keys had names, whereas species remained nameless (Gombosi, 1951).

^17^Greeks used not 7—but 9 PCs named after lyre’s strings (West, 1992, p. 219), related to the position of a pitch not in octave, but 4th.

^18^There are few archaic vocal cultures that explore partials (Sygyt, Khoomei), but they sustain the drone fundamental, require virtuosic skills, and were not practiced by females (Alekseyev and Levin, 1990).

^19^Terhardt (1974) explains how both principles that govern instrumental tuning originate in attempts to sing a melodic intonation. In effect, the singer would be negotiating the best possible compromise between the harmonic spectrum of a musical tone and the melodic shift from pitch to pitch—setting the paradigm for tuning all instruments.

^20^There is no need for fine voluntary breathing control in speech to the extent that it is required by music: normally, speakers breath twice more frequently than do singers, and they engage only a fraction of their lungs’ capacity, whereas singers spend nearly all of their lungs’ capacity and engage all their major respiratory muscles (Fitch, 2006).

^21^Bowling (2013) reviewed all psychoacoustic theories that could account for interval-size similarities between musical and verbal vocalization, confirming Spencer’s opinion that melody imitates speech by exaggerating the verbal intonations. This connection was emphasized by Bartok and Kodaly, elucidated by Kodaly’s pupil, Szabolcsi (1965, p. 199).

^22^Different areas of the brain are engaged in making sense of melody and lyrics, and their processual independence characterizes both, musicians and non-musicians’ hearing (Bonnel et al., 2001). Parallel processing is common in perception of *familiar* songs while processing of *unfamiliar* songs includes phases of integration of melodic and verbal information during the pre-syntactic phonological processing of song’s lyrics (Sammler et al., 2010).

^23^Lomax (1978, pp. 71–73) developed a system of categorization in vocal width, nasality, tremolo, embellishment and rasp for measurements of singing styles.

^24^Noteworthy are the imitations of the babies’ babbling in Yukaghir and Nganasan lullabies, or wolves howling in Kysyl and Vadul animal songs (Sheikin, 2002, p. 169). Equally picturesque are the “throat rasping” vocal style of the Nganasan imitations of the bird calls or growling of a bear (Ojamaa, 2005).

^25^In 1930’s, every Chukcha and Eskimo had his own personal melody to be re-texted on different occasions, i.e., the change in weather, a hunt outcome (Zemtsovsky, 1983, p. 10). Permanence of melodic formula makes it into a “thing-in-itself”—to such extent that these songs were traded and given as gifts.

^26^Greek “khasma” (hollow) corresponds to the English “chasm” (fissure) in reference to such melodies where one tone breaks apart from the rest of the tones—separated with an unclosed gap that pertains throughout the melody.

^27^The modern day Australian Aborigines singing bears khasmatonal traces. Walker (1997) measured the deviations in singing the “same” pitch by 10 opera singers vs. 10 Aborigines singers. The Aborigines deviated 30% more than the opera singers, and used considerably more timbral variation—for the 4th formant, the mean bandwidth was about 250% greater than that of the opera singers’.

^28^Changes in timbre are known to be used for marking the boundaries between groups of pitches, serving as a syntactic tool in musical communication (Sundberg, 1992).

^29^Mansy/Khanty clappers were once sticks that were used for hunting, transformed into ritual representation of a crane. The drum-log of Sakhalin population was once the tree, hit in order to lurk the bear into a trap—then turned into a ritual instrument during the annual bear festival (Sheikin, 2002, p. 51).

^30^The first established samples of art (Fumane-Cave painted blocks, 30–35,000) and music (Divje-Babe pipe, 47,000, see Appendix II) concur with appearance of burials—including Neanderthal. Rarity of evidence of symbolism before the mid-Upper Paleolithic testifies that art was not common back then, so ritualistic music could have provided a prototype for artistic behaviors.

^31^Many of discovered cave lithophones are chipped. Located at spacious areas, their paint is visible only from a close distance (Morley, 2013, p. 119), suggesting that music users gathered in close proximity and likely sang.

^32^West (1981) links the Sanskrit hymnody to Homeric epics and Balkan/Nordic folklore regarding them as the Indo-European tradition of melody-making derived from words in half-singing-half-narrating performance.

^33^The term “ekmelic” was used by Cleonides, Gaudentius, and Ptolemy to refer to disproportionate sounds, opposing “emmelic”—sounds suitable for music-making, according to Aristoxenus.

^34^Ekmelic intervals can vary in absolute size from minor 2nd to tritone, reaching 3rd as an increment between the degrees in a 4-tone mode, and 4th in a 3-tone. Furthermore, any of these intervals can contract or expand throughout a music work, causing the intervallic zones between different degrees to *interlace* (Alekseyev, 1976, p. 123).

^35^“Complimenting” arises where one tone receives consistent stress, whereas another tone stays auxiliary throughout a formula.

^36^“Opposing” can be of two types: where two adjacent stressed tones compete for attention throughout the formula (then larger interval reflects disagreement between both involved tones); where two ekmelic 2nds are summed together surrounding the same principal tone (positioned on the “opposite” sides of it). The type-1 encloses two stable, while type-2—two unstable degrees.

^37^“Extreme” can be of two types: a relation between complimenting and opposing tones (2nd+3rd, or 2nd+2nd+2nd), the highest contrast in ekmelics, since one tone is supporting the principal tone, whereas another tone is denying it. However, 4th can also arise between principal and complimenting of the opposite tones (3rd+2nd), presenting a softer version, where one tone is stable and another unstable.

^38^This term (Greek “oligo”—few) was introduced by Kvitka (1971, vol. 1, p. 285) in 1927 to refer to self-contained modes with fewer than 5 degrees.

^39^That is why the contrast between the intervals of 2nd and 3rd can be considered universal across all musical cultures in the world (Nettl, 2000, p. 468).

^40^Fataleka Au-Ero panpipe-bands from Solomon Islands employ equitonic heptatonics (Kaeppler et al., 2013), so as Chopi xylophonists from Uganda (Haddon, 1952), and Nzakara people of Central Africa (Arom, 2004, p. 24).

^41^(1) C-Db, (2) E-F, and (3) G#-A. Singing Setu scale makes it hard to distinguish between symmetric I, III, and V degrees. The logic behind Setu is major 3rd–based vertical harmony: C/E-Db/F-E/G#-F/A.

^42^This method is also uncovered in tuning practices of Bedzan Pygmies (Arom et al., 2007).

^43^Since pitch organization alone could not provide cues for reliable finding of tonic, and required an occurrence of tonic on a metrically strong time.

^44^Beliayev’s disjunct and conjunct “primary” modes are considered principal in a number of Asian pentatonic music theories, i.e. Japanese gagaku system (Karpati, 1983).

^45^Certain types of absolute pitch (Bachem, 1937) are limited to specific timbres and/or pitch-classes (diatonic vs. chromatic) (Deutsch, 2013) which are handled by categorical perception (Rakowski, 1993). There is no reason to exclude non-Western pitch-classes from absolute pitch candidacy (Ross et al., 2005)—as reported by ethnomusicologists in Angola (Kubik, 1980) and Java (van Zanten, 2004) —which then expands the amount of instantly accessible chunks for a multi-cultural AP possessor beyond 12.

^46^Hungarian pentatony is explicable by the contacts of Hungarians with Kazars, Sabirs, and Onogurs (Sipos, 2005, p. 137). The Celtic and other Western islands could be the remnants of a pentatonic stage in tonal evolution, or traces of a mega-wide diaspora of pentatonic proto-culture (Szabolcsi, 1965, p. 216).

^47^Presence of a few pentatonic tunes in a music culture does not necessarily make it “pentatonic”: heptatonic culture can include a pentatonic style (Mazel, 1952, p. 51). In order for a culture to qualify as pentatonic, it has to produce a substantial amount of works where the entire PS is limited to anhemitonic pentatony.

^48^Pentatony was probably used in earlier Sumerian (Dumbrill, 2005, p. 31) and Egyptian music (Sachs, 2008, p. 73)—which by the Hellenic times turned heptatonic (Duchesne-Guillemin, 1981). Hebrew chant (Szabolcsi, 1943), so as Archaic Greek gapped scales headed the same way (Franklin, 2002).

^49^Traditional Chinese music theory describes the “old” yanyue heptatonic scale C-D-E-F#-G-A (Lu-Ting and Kuo-huang, 1982) in second century BC (Chou, 1976) which indicates that despite the decision of earlier theorists and political authorities to commit to the pentatonic order, heptatonic music remained in use.

^50^Music education in modern China is heavily oriented toward Western music—students commonly develop negative attitude toward Chinese traditional music (Ho and Law, 2006). The preference for Western music increases from elementary to graduate school (Hui, 2009), suggesting that exposure to Western heptatony accompanies creativity growth in younger urban population.

^51^A number of cultures that developed pictographic literacy are known to have cultivated pentatonic music—Aztec (Bierhorst, 1985, p. 80), Micmac (Pisani, 2008, p. 29), Ojibwe (Pian et al., 1994, vol. 1, p. 98), early Sumerian (Dumbrill, 2005, p. 31), Helladic (Franklin, 2002), and likely, Hittite Anatolian (Sipos, 2005, p. 3) and Old-Kingdom Egyptian (Sachs, 2008, p. 73).

^52^For Western “popular music” pentatonic/heptatonic hybrid, the corresponding philosophy is supposed shortsightedness of Western knowledge, and eagerness to combine Western rationalism with Eastern “non-conceptual mindfulness” (Weick and Putnam, 2006).

^53^The full version of this paper will be available at academia.edu.

## References

[B1] Adshead S. A. (2004). T'ang China: The Rise of the East in World History. New York, NY: Palgrave Macmillan.

[B2] Aekseyev E. (1976). Problems in Genesis of Mode [Проблемы Формирования Лада]. Moscow: Muzyka [Музыка].

[B3] Alekseyev E. (1986). Early Folkloric Intonation. Pitch Aspect [Раннефольклорное Интонирование: Звуковысотный Аспект]. Moscow: Sovetskii Kompozitor [Сов: композитор].

[B4] Alekseyev E. (1996). On musical embodiment of olonkho [О Музыкальном Воплощении Олонхо], in Yakut Heroic Epos “Mighty Er Sogotokh” [Якутский Героический Эпос “Могучий Эр Соготох''], eds Alekseyev N. A. Yemelyanov N. V. Nazarenko R. B. (Novosibirsk: Nauka), 10 of Monu: 42–72.

[B5] Alekseyev E. (2013). Ethnomusicological experiment: on the way of trial and error [Этномузыковедческий Эксперимент: На Пути Проб И Ошибок], in Music. Performance. Education [Музыка. Исполнительство. Образование], Vol. 4, eds Varlamova A. Pavlova Z. (Yakutsk: University of Republic of Sakha), 162–179.

[B6] Alekseyev E. Levin T. (1990). Tuva: Voices from the Center of Asia. New York, NY: Smithsonian Folkways Recordings. SFW 40017.

[B7] Alekseyev E. Nikolayeva N. (1981). Samples of Yakut Vocal Folklore [Образцы Якутского Песенного Фольклора]. Yakutsk: Academy of Sciences of USSR.

[B8] Altenmuller E. Finger S. Boller F. (eds.). (2015). Music, Neurology, and Neuroscience: Evolution, the Musical Brain, Medical Conditions, and Therapies. Amsterdam: Elsevier Science.

[B9] Ambrazevičius R. Pärtlas Ž. (2011). Searching for the ‘natural’ origins of the symmetrical scales: traditional multipart setu songs. J. Interdiscip. Music Stud. 5, 1–17. 10.4407/jims.2011.07.001

[B10] Ammirante P. Russo F. A. (2015). Low-skip bias. Music Percept. 32, 355–363. 10.1525/mp.2015.32.4.355

[B11] Ammirante P. Thompson W. F. (2012). Continuation tapping to triggered melodies: motor resonance effects of melodic motion. Exp. Brain Res. 216, 51–60. 10.1007/s00221-011-2907-5 22038717

[B12] Arom S. (2004). African Polyphony and Polyrhythm: Musical Structure and Methodology (transl. M. Thom, B. Tuckett, and R. Boyd). Cambridge: Cambridge University Press.

[B13] Arom S. (2010). Corroborating external observation by cognitive data in the description and modelling of traditional music. Musicae Scientiae 14(2 Suppl.), 295–306. 10.1177/10298649100140S216

[B14] Arom S. Fernando-Marandola N. Marandola F. (2007). An innovative method for the study of african musical scales: cognitive and technical aspects, in Proceedings of the 4th Sound and Music Computing Conference, Lefkada, Greece, eds Spyridis C. Georgaki A. Kouroupetroglou G. Anagnostopoulou C. (Athens: University of Athens), 107–116.

[B15] Asafyev B. (1952). Selected Works [Избранные Труды]. Vol. 1 Moscow: Academy of Science of USSR [Изд-во Академии наук СССР].

[B16] Asafyev B. (1971). Musical Form as a Process [Музыкальная Форма Как Процесс]. 2nd Edn Leningrad: Muzyka [Музыка].

[B17] Attneave F. Olson R. K. (1971). Pitch as a medium: a new approach to psychophysical scaling. Am. J. Psychol. 84, 147–166. 10.2307/1421351 5566581

[B18] Babbitt M. (1955). Some aspects of twelve-tone composition, in The Score and IMA Magazine 12 (Princeton, NJ: Princeton University Press), 53–61.

[B19] Bachem A. (1937). Various types of absolute pitch. J. Acoust. Soc. Am. 9, 146–157. 10.1121/1.1915919

[B20] Bakulina E. (2014). The concept of mutability in russian theory. Music Theory Online 20 Available online at: http://www.mtosmt.org/issues/mto.14.20.3/mto.14.20.3.bakulina

[B21] Balzano G. J. (1982). The pitch set as a level of description for studying musical pitch perception, in Music, Mind, and Brain. The Neuropsychology of Music, ed Clynes M. (New York, NY, London: Plenum Press), 321–351.

[B22] Barbieri P. Mangsen S. (1991). Violin intonation: a historical survey. Early Music 19, 69–88. 10.1093/earlyj/XIX.1.69

[B23] Beliaev V. (1959). Early Russian polyphony, in Studia Memoriae Belae Bartók Sacra, ed Rejeczky B. (New York, NY, London: Boosey and Hawkes), 311–332.

[B24] Beliayev V. (1990). Modal systems in the traditional music of the USSR [Ладовые Системы В Музыке Народов СССР], in Viktor Mikhailovich Beliayev [Виктор Михайлович Беляев], ed Travkina I. (Moscow: Sovetskii Kompozitor [Советский композитор]), 223–377.

[B25] Bendixen A. Háden G. P. Németh R. Farkas D. Török M. Winkler I. (2015). Newborn infants detect cues of concurrent sound segregation. Dev. Neurosci. 37, 172–181. 10.1159/000370237 25721916

[B26] Bharucha J. J. (1996). Melodic anchoring. Music Percept. 13, 383–400. 10.2307/40286176

[B27] Biamonte N. (2010). Triadic modal and pentatonic patterns in rock music. Music Theory Spectr. 32, 95–110. 10.1525/mts.2010.32.2.95

[B28] Bidelman G. M. Grall J. (2014). Functional organization for musical consonance and tonal pitch hierarchy in human auditory cortex. Neuroimage 101, 204–214. 10.1016/j.neuroimage.2014.07.005 25019679

[B29] Bierhorst J. (1985). Cantares Mexicanos: Songs of the Aztecs. Redwood City, CA: Stanford University Press.

[B30] Bigand E. (1997). Perceiving musical stability: the effect of tonal structure, rhythm, and musical expertise. J. Exp. Psychol. Hum. Percept. Perform. 23:808. 10.1037/0096-1523.23.3.808 9180045

[B31] Bigand E. Poulin B. Tillmann B. Madurell F. D'Adamo D. A. (2003). Sensory versus cognitive components in harmonic priming. J. Exp. Psychol. Hum. Percept. Perform. 29, 159–171. 10.1037/0096-1523.29.1.159 12669755

[B32] Blacking J. (1974). How Musical Is Man? Seattle, WA: University of WashingtonPress.

[B33] Blum S. (1985). Rousseau's Concept of ‘Sisteme Musical’ and the comparative study of tonalities in nineteenth-century France. J. Am. Musicol. Soc. 38, 349–361. 10.2307/831568

[B34] Boersma P. Kovacic G. (2006). Spectral characteristics of three styles of croatian folk singing. J. Acoust. Soc. Am. 119, 1805–1816. 10.1121/1.2168549 16583921

[B35] Bonnard D. Micheyl C. Semal C. Dauman R. Demany L. (2012). Auditory discrimination of frequency ratios: the octave singularity. J. Exp. Psychol. Hum. Percept. Perform. 39, 788–801. 10.1037/a0030095 23088507 PMC3905309

[B36] Bonnel A.-M. Faita F. Peretz I. Besson M. (2001). Divided attention between lyrics and tunes of operatic songs: evidence for independent processing. Percept. Psychophys. 63, 1201–1213. 10.3758/BF03194534 11766944

[B37] Borchert E. M. O. Micheyl C. Oxenham A. J. (2011). Perceptual grouping affects pitch judgments across time and frequency. J. Exp. Psychol. 37, 257–269. 10.1037/a0020670 21077719 PMC3057773

[B38] Both A. A. (2009). Music archaeology: some methodological and theoretical considerations, in Yearbook for Traditional Music, eds Niles D. Both A. A. (Ljubljana: International Council for Traditional Music), 1–11. Available online at: http://www.jstor.org/stable/25735475

[B39] Boulton L. (1955). The Eskimos of Hudson Bay and Alaska. New York, NY: Smithsonian Folkways Recordings. FW04444/FE 4444.

[B40] Bowling D. L. (2012). The Biological Basis of Emotion in Musical Tonality. Durham, NC: Duke University.

[B41] Bowling D. L. (2013). A vocal basis for the affective character of musical mode in melody. Front. Psychol. 4:464. 10.3389/fpsyg.2013.00464 23914179 PMC3728488

[B42] Bradley E. (2013). Pitch perception in lexical tone and melody. Rev. Res. Hum. Learn. Music. 1. 10.6022/journal.rrhlm.201300222362607

[B43] Brandl R. (2008). New considerations of diaphony in Southeast Europe, in European Voices: Multipart Singing in the Balkans and the Mediterranean, Vol. 1, eds Ahmedaja A. Haid G. (Vienna: Böhlau Verlag), 281.

[B44] Brattico E. Tervaniemi M. Näätänen R. Peretz I. (2006). Musical scale properties are automatically processed in the human auditory cortex. Brain Res. 1117, 162–174. 10.1016/j.brainres.2006.08.023 16963000

[B45] Bregman A. (1994). Auditory Scene Analysis: The Perceptual Organization of Sound. Cambridge MA: MIT Press.

[B46] Bregman A. Woszczyk W. (2004). Controlling the perceptual organization of sound: guidelines derived from principles of auditory scene analysis (ASA), in Audio Anecdotes: Tools, Tips and Techniques for Digital Audio, Vol. 1, eds Greenebaum K. Barzel R. (Natick, MA: A.K. Peters), 33–61.

[B47] Bretèque E. A. (2012). Voices of sorrow: melodized speech, laments, and heroic narratives among the yezidis of armenia. Yearb. Tradit. Music 44, 129–148. 10.5921/yeartradmusi.44.0129

[B48] Brown H. Butler D. (1981). Diatonic trichords as minimal tonal cue-cells. Theory Only 5, 37–55.

[B49] Brown S. (2007). Contagious heterophony: a new theory about the origins of music. Musicae Scientiae 11, 3–26. 10.1177/102986490701100101

[B50] Bruckert L. Bestelmeyer P. Latinus M. Rouger J. Charest I. Rousselet G. A.. (2010). Vocal attractiveness increases by averaging. Curr. Biol. 20, 116–120. 10.1016/j.cub.2009.11.034 20129047

[B51] Burnett H. (1980). An introduction to the history and aesthetics of japanese jiuta-tegotomono. Asian Music 11, 11–40. 10.2307/834064

[B52] Burns E. M. Ward W. D. (1999). Intervals, scales, and tuning, in The Psychology of Music, 2nd Edn., ed Deustch D. (New York, NY: Academic Press), 215–264.

[B53] Butler D. Brown H. (1994). Describing the mental representation of tonality in music, in Musical Perceptions, eds Aiello R. Sloboda J. A. (London: Oxford University Press), 191–212.

[B54] Bytchkov Y. (1997). On Dialectics of Making and Unfolding of Mode [О Диалектике Становления И Развертывания Лада]. Moscow: Gnessin Russian Academy of Music [Российская академия музыки имени Гнесиных].

[B55] Cable T. (1975). Parallels to the melodic formulas of ‘Beowulf.’ Mod. Philol. 73, 1–14. 10.1086/390614

[B56] Cambouropoulos E. (2008). Voice and stream: perceptual and computational modeling of voice separation. Music Percept. 26, 75–94. 10.1525/mp.2008.26.1.75

[B57] Canzio R. (1989). Brazil: Bororo World of Sound. Paris: Audivis UNESCO. D 8201.

[B58] Carlsen J. C. (1981). Some factors which influence melodic expectancy. Psychomusicology 1, 12–29. 10.1037/h0094276

[B59] Castellano M. A. Bharucha J. J. Krumhansl C. L. (1984). Tonal hierarchies in the music of North India. J. Exp. Psychol. Gen. 113:394. 10.1037/0096-3445.113.3.394 6237169

[B60] Cazden N. (1958). Pythagoras and aristoxenos reconciled. J. Am. Musicol. Soc. 11, 97–105. 10.2307/829897

[B61] Cazden N. (1971). A simplified mode classification for traditional Anglo-American song tunes, in Yearbook of the International Folk Music Council, Urbana, IL, ed Haywood C. (Champaign, IL: University of Illinois Press), 45–78.

[B62] Chou W.-C. (1976). Chinese historiography and music; some observations. Musical Q. 62, 218–240.

[B63] Christensen T. (ed.). (2008). The Cambridge History of Western Music Theory. Cambridge: Cambridge University Press.

[B64] Choron A. E. Fayolle F. J. M. (1810). Dictionnaire Historique Des Musiciens, Artistes et Amateurs Morts Ou Vivans. Vol. 1 Paris: Valade & Lenormant.

[B65] Clarke E. F. (2001). Meaning and the specification of motion in music. Musicae Scientiae 5, 213–234. 10.1177/102986490100500205

[B66] Clarke E. F. (2005). Ways of Listening: An Ecological Approach to the Perception of Musical Meaning. Oxford; New York, NY: Oxford University Press.

[B67] Conard N. J. (2011). Neanderthal lifeways, subsistence and technology, in Neanderthal Lifeways, Subsistence and Technology: One Hundred Fifty Years of Neanderthal Study, Vol. 19, Vertebrate Paleobiology and Paleoanthropology, eds Conard N. J. Richter J. (Dordrecht: Springer), 223–240.

[B68] Cook N. D. (2002). Tone of Voice and Mind: The Connections between Intonation, Emotion, Cognition and Consciousness. Amsterdam: John Benjamins Publishing.

[B69] Cooke D. (1959). The Language of Music. London: Oxford University Press.

[B70] Crocker R. L. (1997). Discant, counterpoint, and harmony, in Studies in Medieval Music Theory and the Early Sequence (Aldershot: Variorum), 1–21.

[B71] Cross I. (2007). Music and cognitive evolution, in Handbook of Evolutionary Psychology, eds Dunbar R. Barrett L. (Oxford: Oxford University Press), 649–667.

[B72] Cross I. Morley I. (2009). The evolution of music: theories, definitions and the nature of the evidence, in Communicative Musicality: Exploring the Basis of Human Companionship, eds Trevarthen C. Malloch S. (London: Oxford University Press), 61–82.

[B73] Cuddy L. L. (1997). Tonal relations, in Perception and Cognition of Music, eds Deliège I. Sloboda J. A. (Hove: Psychology Press), 330–352.

[B74] Dams L. (1985). Palaeolithic lithophones: descriptions and comparisons. Oxford J. Archaeol. 4, 31–46. 10.1111/j.1468-0092.1985.tb00229.x

[B75] Daniélou A. (1995). Music and the Power of Sound: The Influence of Tuning and Interval on Consciousness. Rep Sub Edn. Rochester, VT: Inner Traditions.

[B76] Davidson L. (1985). Tonal structures of children's early songs. Music Percept. 2, 361–373. 10.2307/40285304

[B77] Davidson L. (1994). Songsinging by young and old: a developmental approach to music, in Musical Perceptions, eds Aiello L. C. Sloboda J. (Oxford, UK: Oxford University Press), 99–130.

[B78] Day-O'Connell J. (2007). Pentatonicism from the Eighteenth Century to Debussy. Rochester, NY: University of Rochester Press.

[B79] Demay L. Péan S. Patou-Mathis M. (2012). Mammoths used as food and building resources by Neanderthals: zooarchaeological study applied to Layer 4, Molodova I (Ukraine). Quat. Int. 276–277, 212–226. 10.1016/j.quaint.2011.11.019

[B80] Desjacques A. (1991). Mongolia: Traditional Music. Paris: Audivis UNESCO. D 8207.

[B81] Deutsch D. (ed.). (2013). Absolute pitch, in Psychology of Music (New York, NY: Academic Press), 142–182.

[B82] Devereux P. (2006). Ears and years: aspects of acoustics and intentionality in antiquity, in Archaeoacoustics, eds Scarre C. Lawson G. (Cambridge: McDonald Institute for Archaeological Research), 23–30.

[B83] Díaz-Andreu M. García B. (2012). Acoustics and levantine rock art: auditory perceptions in la valltorta gorge (Spain). J. Archaeol. Sci. 39, 3591–3599. 10.1016/j.jas.2012.06.034

[B84] Dissanayake E. (2004). Motherese is but one part of a ritualized, multimodal, temporally organized, affiliative interaction. Behavi. Brain Sci. 27, 512–513. 10.1017/S0140525X0432011X

[B85] Dissanayake E. (2013). Born to artify: the universal origin of picturing, in Origins of Pictures: Anthropological Discourses in Image Science, eds Sachs-Hombach K. Schirra J. (Koln: Herbert von Halem Verlag), 230–249.

[B86] Dogantan-Dack M. (2013). Tonality: the shape of affect. Empir. Musicol. Rev. 8, 208–218. Available online at: http://emusicology.org/article/view/3943

[B87] Dolscheid S. Hunnius S. Casasanto D. Majid A. (2014). Prelinguistic infants are sensitive to space-pitch associations found across cultures. Psychol. Sci. 25, 1256–1261. 10.1177/0956797614528521 24899170

[B88] Dowling W. J. (1967). Rhythmic Fission and the Perceptual Organization of Tone Sequences. Cambridge, MA: Harvard University.

[B89] Duchesne-Guillemin M. (1981). Music in ancient mesopotamia and Egypt author. World Archaeol. 12, 287–297. 10.1080/00438243.1981.9979803

[B90] Dumbrill R. (2005). The Archaeomusicology of the Ancient Near East. Victoria: Trafford Publishing.

[B91] Dunbar R. (2012). On the evolutionary function of song and dance, in Music, Language, and Human Evolution, ed N. Bannan (Oxford: Oxford University Press), 201–214.

[B92] Eitan Z. (1993). Melodic contour and musical style: a quantitative study. Musikometrika 5, 1–68.

[B93] Eitan Z. (2013). Which is more? pitch height, parametric scales, and the intricacies of cross-domain magnitude relationships, in Musical Implications: Essays in Honor of Eugene Narmour, L. F. Bernstein and A. Rozin (Hillsdale, NY: Pendragon Press), 131–148.

[B94] Eitan Z. Granot R. Y. (2006). How music moves. Music Percept. 23, 221–248. 10.1525/mp.2006.23.3.221

[B95] Fais L. Leibowich J. Hamadani L. Ohira L. (2010). Infant movement as a window into language processing. Gesture 10, 222–250. 10.1075/gest.10.2-3.06fai

[B96] Fancourt A. Dick F. Stewart L. (2013). Pitch-change detection and pitch-direction discrimination in children. Psychomusicology 23, 73–81. 10.1037/a0033301

[B97] Fenk-Oczlon G. Fenk A. (2009). Some parallels between language and music from a cognitive and evolutionary perspective. Musicae Scientiae 13(2 Suppl.), 201–226. 10.1177/1029864909013002101

[B98] Fernando-Marandola N. (2007). Study of African scales: a new experimental approach for cognitive aspects. Rev. Transcult. Música 11.

[B99] Fink R. (2003). On the Origin of Music-An Integrated Overview of the Origin and Evolution of Music. Saskatoon, SK: Greenwich.

[B100] Fitch W. T. (2006). The biology and evolution of music: a comparative perspective. Cognition 100, 173–215. 10.1016/j.cognition.2005.11.009 16412411

[B101] Foley R. Lahr M. M. (2003). On stony ground: lithic technology, human evolution, and the emergence of culture. Evol. Anthropol. 12, 109–122. 10.1002/evan.10108

[B102] Forte A. (1964). A theory of set-complexes for music. J. Music Theory 8, 136 10.2307/843079

[B103] Fox D. B. (1990). An analysis of the pitch characteristics of infant vocalizations. Psychomusicology 9, 21–30.

[B104] Franklin J. C. (2002). Diatonic music in greece: a reassessment of its antiquity. Mnemosyne 55, 669–702. 10.1163/156852502320880186

[B105] Frolov B. (1992). Primitive Graphics of Europe [Первобытная Графика Европы]. Moscow: Nauka.

[B106] Frolov B. (2003). Genesis of graphic symbolization [Генезис Графической Символики], in Chinese Classic “Book of Changes” and Modern Science [Китайская Классическая “Книга Перемен” И Современная Наука], ed P. M. Kozhin (Moscow: Luch [Луч]), 20–28.

[B107] Furniss I. (2009). Unearthing China's informal musicians: an archaeological and textual study of the shang to tang periods, in Yearbook for Traditional Music, eds Niles D. Both A. A. (Ljubljana: International Council for Traditional Music), 23–41. Available online at: http://www.jstor.org/stable/25735477

[B108] Gauldin R. (1983). The cycle-7 complex: relations of diatonic set theory to the evolution of ancient tonal systems. Music Theory Spectr. 5, 39–55. 10.1525/mts.1983.5.1.02a00030

[B109] Gelbart M. (2013). Once more to mendelssohn's scotland: the laws of music, the double tonic, and the sublimation of modality. 19th-Century Music 37, 3–36. 10.1525/ncm.2013.37.1.3.

[B110] GiliarovaN. (2010). The Registry of Expeditional and Stationary Audio Recordings of the Main Fund of the Scientific Center of Folk Music [Перечень Экспедиционных И Стационарных Аудиозаписей Фонда Кабинета Народной Музыки], 3rd Edn. Moscow: Moscow Conservatory [Московская государственная консерватория имени П. И. Ча 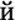 ковского].

[B111] Gill K. Z. Purves D. (2009). A biological rationale for musical scales. PLoS ONE 4:e8144. 10.1371/journal.pone.0008144 19997506 PMC2779864

[B112] Godøy R. I. (2013). Quantal elements in musical experience, in Sound-Perception-Performance, ed R. Bader (Heidelberg: Springer), 113–128

[B113] Gombosi O. (1951). Key, mode, species. J. Am. Musicol. Soc. 4, 20–26. 10.2307/830117

[B114] Goodman N. (1976). Languages of Art: An Approach to a Theory of Symbols, 2nd Edn. Indianapolis, IL: Hackett Publishing.

[B115] Graf W. T. (1967). Zur Gesanglichen Stimmgebung Der Ainu, in Festschrift F¨ur Walter Wiora Zum 30. Dezember 1966 eds Finscher L. Mahling C.-H. (Kassel: B¨arenreiter), 529–535.

[B116] Grauer V. A. (2006). Echoes of our forgotten ancestors. World Music 48, 5–58. 10.2307/41699716

[B117] Grauer V. A. (2007). New perspectives on the kalahari debate: a tale of two ‘Genomes. Before Farming 2:4 10.3828/bfarm.2007.2.4

[B118] Graves J. E. Micheyl C. Oxenham A. J. (2014). Expectations for melodic contours transcend pitch. J. Exp. Psychol. 40, 2338–2347. 10.1037/a0038291 25365571 PMC4605576

[B119] Gruhn W. (1998). Der Musikverstand: Neorobiologische Grundlagen Des Musikalischen Denkens, Hörens Und Lernens. Hildesheim; New York, NY: Georg Olms.

[B120] Gussenhoven C. (2002). Intonation and interpretation: phonetics and phonology, in Proceedings of Speech Prosody, eds Bel E. Marilier I. (Aix-enProvence: University de Provence), 45–57.

[B121] Gutschalk A. Uppenkamp S. (2011). Sustained responses for pitch and vowels map to similar sites in human auditory cortex. Neuroimage 56, 1578–1587. 10.1016/j.neuroimage.2011.02.026 21335091

[B122] Haddon E. (1952). Possible origin of the chopi timbila xylophone. Afr. Music Soc. Newsl. 1, 61–67.

[B123] Háden G. Stefanics G. Vestergaard M. D. Denham S. L. Sziller I. Winkler I. (2009). Timbre-independent extraction of pitch in newborn infants. Psychophysiology 46, 69–74. 10.1111/j.1469-8986.2008.00749.x 19055501 PMC2833415

[B124] Háden G. P. Honing H. T¨or¨ok M. Winkler I. (2015). Detecting the temporal structure of sound sequences in newborn infants. Int. J. Psychophysiol. 96, 23–28. 10.1016/j.ijpsycho.2015.02.024 25722025

[B125] Hagel S. (2009). Ancient Greek Music: A New Technical History. New York, NY: Cambridge University Press.

[B126] Hair H. (1995). Mood categories of lines, colors, words, and music. Bull. Counc. Res. Music Educ. 127, 99–105.

[B127] Halberstadt J. (2006). The generality and ultimate origins of the attractiveness of prototypes. Pers. Soc. Psychol. Rev. 10, 166–183. 10.1207/s15327957pspr1002_5 16768653

[B128] Hall D. E. Hess J. T. (1984). Perception of musical interval tuning. Music Percept. 2, 166–195. 10.2307/40285290

[B129] Halpern I. (1974). Nootka Indian Music of the Pacific North West Coast. New York, NY: Smithsonian Folkways Recordings. FW04524/FE 4524.

[B130] Han S. Sundararajan J. Bowling D. L. Lake J. Purves D. (2011). Co-variation of tonality in the music and speech of different cultures. PLoS ONE 6:e20160. 10.1371/journal.pone.0020160 21637716 PMC3103533

[B131] Handschin J. (1995). Der Toncharakter: Eine Einf¨uhrung in Die Tonpsychologie, 2nd Edn. Darmstadt: Wissenschaftliche Buchgesellschaft.

[B132] Hannas W. C. (2013). The Writing on the Wall: How Asian Orthography Curbs Creativity. Philadelphia, PA: University of Pennsylvania Press.

[B133] Hargreaves D. J. (1986). The Developmental Psychology of Music. Cambridge, UK: Cambridge University Press.

[B134] Haynes A. (2004). Meditation and health: an annotated bibliography. Ref. User Serv. Q. 44, 18–25. Available online at: http://hdl.handle.net/2022/453

[B135] Heylen L. Wuyts F. L. Mertens F. De Bodt M. Van de Heyning P. H. (2002). Normative voice range profiles of male and female professional voice users. J. Voice 16, 1–7. 10.1016/S0892-1997(02)00065-6 12002876

[B136] Ho W.-C. Law W.-W. (2006). Challenges to globalisation, localisation and sinophilia in music education: a comparative study of Hong Kong, Shanghai and Taipei. Br. J. Music Educ. 23, 217–237. 10.1017/S0265051706006942

[B137] Holleran S. Jones M. R. Butler D. (1995). Perceiving implied harmony: the influence of melodic and harmonic context. J. Exp. Psychol. Learn. Mem. Cogn. 21:737. 10.1037/0278-7393.21.3.737 7602268

[B138] Honing H. (2003). The final ritard: on music, motion, and kinematic models. Comput. Music J. 27, 66–72. 10.1162/014892603322482538

[B139] Honing H. Ploeger A. (2012). Cognition and the evolution of music: pitfalls and prospects. Top. Cogn. Sci. 4, 513–524. 10.1111/j.1756-8765.2012.01210.x 22760967

[B140] Hubbard T. L. Courtney J. R. (2010). Cross-modal influences on representational momentum and representational gravity. Perception 39, 851–862. 10.1068/p6538 20698479

[B141] Hubbard T. L. Ruppel S. E. (2013). A Fr¨ohlich effect and representational gravity in memory for auditory pitch. J. Exp. Psychol. 39, 1153–1164. 10.1037/a0031103 23317117

[B142] Huddleston W. E. Lewis J. W. Phinney R. E. Jr. DeYoe E. A. (2008). Auditory and visual attention-based apparent motion share functional parallels. Percept. Psychophys. 70, 1207–1216. 10.3758/PP.70.7.1207 18927004

[B143] Hui W. V. (2009). Music listening preferences of macau students. Music Educ. Res. 11, 485–500. 10.1080/14613800903391749

[B144] Huron D. (1994). Interval-class content in equally tempered sets: common scales exhibit optimum tonal consonance. Music Percept. 11, 289–305. 10.2307/40285624

[B145] Huron D. (2001). Tone and voice: a derivation of the rules of voice-leading from perceptual principles. Music Percept. 19, 1–64. 10.1525/mp.2001.19.1.1

[B146] Huron D. (2006). Sweet Anticipation: Music and the Psychology of Expectation. Cambridge, MA: MITPress.

[B147] Hutchinson W. Knopoff L. (1978). The acoustic component of western consonance. Interface 7, 1–29.

[B148] Ilie G. Thompson W. F. (2006). A comparison of acoustic cues in music and speech for three dimensions of affect. Music Percept. 23, 319–330. 10.1525/mp.2006.23.4.319

[B149] Ivanova I. Zeitlin S. (1987). The Multilayered Paleolithic Site Moldova V. The Stone Age Men and Environment [Многослойная Палеолитическая Стоянка Молодова V. Люди Каменного Века И Окружающая Среда]. Moscow: Nauka.

[B150] Jerison H. (1973). Evolution of The Brain and Intelligence. New York, NY: AcademicPress.

[B151] Johnson-Laird P. N. Oatley K. (2010). Emotions, music, and literature in Handbook of Emotions, 3rd Edn., eds Lewis M. Haviland-Jones J. M. Barrett L. F. (New York, NY: The Guilford Press), 102–113.

[B152] Jordania J. (2005). ‘Interrogo Ergo Cogito’: ‘I Am Asking Questions, Therefore I think'-responsorial singing and the origins of human intelligence, in Proceedings: The Second International Symposium on Traditional Polyphony: 23–27 September, 2004 (Tbilisi).

[B153] Jordania J. (2006). Who Asked the First Question? The Origins of Human Choral Singing, Intelligence, Language and Speech. The Origins of Human Choral Singing, Intelligence,.… Tbilisi: Logos.

[B154] Jordania J. (2008). Music and emotion: humming in the beginning of human history, in The Fourth International Symposium on Traditional Polyphony, Tbilisi, Georgia, ed R. Tsurtsumia (New York, NY: Nova Science), 41–49.

[B155] Jordania J. (2011). Why Do People Sing? Music in Human Evolution. Tbilisi: Logos.

[B156] Juhász Z. (2012). A mathematical study of note association paradigms in different folk music cultures. J. Math. Music 6, 169–185. 10.1080/17459737.2012.740574

[B157] Kaeppler A. Niles D. Chenoweth V. Love J. W. Zemp H. (2013). Solomon Islands, in The Concise Garland Encyclopedia of World Music, Vol. 1, ed K. Ellen (London: Routledge), 682–688.

[B158] Karpati J. (1980). Myth and reality in the theory of chinese tonal system. Stud. Musicol. Acad. Sci. Hung. 22, 5–14. 10.2307/901989

[B159] Karpati J. (1983). Tonality in Japanese court music. Stud. Musicol. Acad. Sci. Hung. 4, 171–182. 10.2307/901970

[B160] Kharlap M. (1972). Traditional russian musical system and the problem of origin of music [Народно-Русская Музыкальная Система И Проблема Происхождения Музыки], in Early Forms of Art [Ранние Формы Искусства], ed Y. Meletinskii (Moscow: Iskusstvo), 246–247.

[B161] Kholopov Y. (1975). Modal harmony: modality as a type of structure [Модальная Гармония: Модальность Как Тип Структуры], in Art of Music: General Matters of Theory and Esthetics of Music [Музыкальное Искусство. Общие Вопросы Теории И Эстетики Музыки], ed T. Solomonova (Tashkent: Gafur Guliam [Издательство литературы и искусства имени Гафура Гуляма]), 16–31.

[B162] Kholopov Y. (1982). Mode [Лад]. Encyclopedia of Music [Музыкальная Энциклопедия]. Soviet Encyclopedia [Советская энциклопедия] (Moscow).

[B163] Kholopov Y. (1988). Harmony: A Theoretic Course [Гармония: теоретический курс]. Moscow: Muzyka [Музыка].

[B164] Kholopov Y. (2005). Towards the problem of mode in russian theoretic musicology [К Проблеме Лада В Русском Теоретическом Музыкознании], in Harmony: Problems of Science and Methodology [Гармония: Проблемы Науки И Методики], Vol. 2, ed E. Struchalina (Rostov-na-Donu: RGK [Ростовская государственная консерватория]), 135–157.

[B165] Kholopov Y. (2006). Musical-Theoretic Systems [Музыкально-Теоретические Системы]. Moscow: Kompozitor.

[B166] Koda H. Basile M. Olivier M. Remeuf K. Nagumo S. Blois-Heulin C.. (2013). Validation of an auditory sensory reinforcement paradigm: Campbell's Monkeys (Cercopithecus Campbelli) do not prefer consonant over dissonant sounds. J. Comp. Psychol. 127, 265–271. 10.1037/a0031237 23566027

[B167] Kolinski M. (1967). Recent trends in ethnomusicology. Proc. R. Musical Assoc. 11, 1–24. 10.2307/850496

[B168] Kolinski M. (1978). The structure of music: diversification versus constraint. Ethnomusicology 22, 229–244. 10.2307/851488

[B169] Kolinski M. (1990). Classification of tonal structures, in Cross-Cultural Musical Analysis, ed K. Shelemay (New York, NY: General Music Publishing Co), 157–195.

[B170] Komar A. J. (1971). Theory of Suspensions: A Study of Metrical and Pitch Relations in Tonal Music. Princeton, NY: Princeton University Press.

[B171] Konson G. (2010). International Conference Musicological Forum [Музыковедческий Форум]. Moscow: State Institute of Art Studies, Russian Musical Academy [Государственный институт искусствознания, Российская академия музыки имени Гнесиных].

[B172] Kraus R. C. (1989). Pianos and Politics in China: Middle-Class Ambitions and the Struggle over Western Music. Oxford; New York, NY: Oxford University Press.

[B173] Krumhansl C. L. (1979). The psychological representation of musical pitch in a tonal context. Cogn. Psychol. 11, 346–374. 10.1016/0010-0285(79)90016-1

[B174] Krumhansl C. L. (1987). General properties of musical pitch systems: some psychological considerations, in Harmony and Tonality, Vol. 54, ed J. Sundberg (Stockholm: Royal Swedish Academy), 33–52.

[B175] Krumhansl C. L. (1990). Cognitive Foundations of Musical Pitch. New York, NY: Oxford University Press.

[B176] Krumhansl C. L. (1996). A perceptual analysis of Mozart's Piano Sonata K.282: segmentation, tension, and musical ideas. Music Percept. 13, 401–432. 10.2307/40286177

[B177] Krumhansl C. L. (2002). Music: A link between cognition and emotion. Curr. Dir. Psychol. Sci. 11, 45–50. 10.1111/1467-8721.00165

[B178] Krumhansl C. L. Cuddy L. L. (2010). A theory of tonal hierarchies in music, in Music Perception (Springer Handbook of Auditory Research Vol. 36), eds Jones M. R. Fay R. R. Popper A. N. (New York, NY: Springer), 51–87.

[B179] Kubik G. (1980). Likembe Tunings of Kufuna Kandonga (Angola). African Music 6, 70–88.

[B180] Kubik G. (1985). African tone-systems: a reassessment. Yearb. Tradit. Music 17, 31–63. 10.2307/768436

[B181] Kubik G. (2010). Theory of African Music, Vol. 1. Chicago, IL: University of Chicago Press.

[B182] Kvitka K. V. (1971). Selected Works [Избранные Труды], Vol. 1 ed V. L. Goshovskii. Moscow: Sovetskii Kompozitor [Сов. композитор].

[B183] Kvitka K. V. (1973). Selected Works [Избранные Труды], Vol. 2 ed V. L. Goshovskii. Moscow: Sovetskii Kompozitor [Советский композитор].

[B184] Langlois J. H. Roggman L. (1990). Attractive faces are only average. Psychol. Sci. 1, 115–121. 10.1111/j.1467-9280.1990.tb00079.x

[B185] Lantz M. E. Kim J.-K. Cuddy L. L. (2014). Perception of a tonal hierarchy derived from korean *music*. Psychol. Music 42, 580–598. 10.1177/0305735613483847

[B186] Larson S. (1997). The problem of prolongation in ‘Tonal’ music: terminology, perception, and expressive meaning. J. Music Theory 41, 101 10.2307/843763

[B187] Larson S. (2012). Musical Forces: Motion, Metaphor, and Meaning in Music. Bloomington, IN: Indiana University Press.

[B188] Larson S. McAdams S. (2004). Musical forces and melodic expectations: comparing computer models and experimental results. Music Percept. 21, 457–498. 10.1525/mp.2004.21.4.457

[B189] Larson S. Vanhandel L. (2005). Measuring musical forces. Music Percept. 23, 119-136. 10.1525/mp.2005.23.2.119

[B190] Lawergren B. (1988). The origins of musical instruments and sounds. Anthropos 83, 31–45.

[B191] Lehne M. Rohrmeier M. Gollmann D. Koelsch S. (2013). The influence of different structural features on felt musical tension in two piano pieces by mozart and mendelssohn. Music Percept. 31, 171–185. 10.1525/mp.2013.31.2.171

[B192] Lehne M. Rohrmeier M. Koelsch S. (2014). Tension-related activity in the orbitofrontal cortex and amygdala: an fMRI study with music. Soc. Cogn. Affect. Neurosci. 9, 1515–1523. 10.1093/scan/nst141 23974947 PMC4187266

[B193] Léothaud G. Voisin F. Lamont A. Arom S. (1997). Experimental ethnomusicology: an interactive approach to the study of musical scales, in Perception and Cognition of Music, eds Deliège I. Sloboda J. A. (Hove: Psychology Press), 3–30.

[B194] Lerdahl F. (2009). Genesis and architecture of the GTTM project. Music Percept. 26, 187–194. 10.1525/mp.2009.26.3.187

[B195] Lerdahl F. Krumhansl C. L. (2007). Modeling tonal tension. Music Percept. 24, 329–366. 10.1525/mp.2007.24.4.329

[B196] Levin T. (1999). Tuva, Among the Spirits: Sound, Music, and Nature in Sakha and Tuva. New York, NY: Smithsonian Folkways Recordings. SFW 40452.

[B197] Lewin D. (1960). Re: the intervallic content of a collection of notes, intervallic relations between a collection of notes and its complement: an application to Schoenberg's hexachordal pieces. J. Music Theory 4, 98–101.

[B198] Li X. Harbottle G. Zhang J. Wang C. (2003). The earliest writing? Sign use in the seventh millennium BC at Jiahu, Henan Province, China. Antiquity 77, 31–44. 10.1017/S0003598X00061329

[B199] Lidji P. Jolicoeur P. Kolinsky R. Moreau P. Connolly J. F. Peretz I. (2010). Early integration of vowel and pitch processing: a mismatch negativity study. Clin. Neurophysiol. 121, 533–541. 10.1016/j.clinph.2009.12.018 20071227

[B200] Liégeois-Chauval C. Peretz I. Babaï M. Laguitton V. Chauvel P. (1998). Contribution of different cortical areas in the temporal lobes to music processing. Brain 121, 1853–1867. 9798742 10.1093/brain/121.10.1853

[B201] List G. (1985). Hopi melodic concepts. J. Am. Musicol. Soc. 38, 143–152.

[B202] List G. (1987). Stability and variation. Ethnomusicology 31, 18–34.

[B203] List G. (2008). The boundaries of speech and song, in Music, Words and Voice: A Reader, ed M. Clayton (Manchester: Manchester University Press), 24–32.

[B204] Litovsky R. (2015). Development of the auditory system, in The Human Auditory System: Fundamental Organization and Clinical Disorders, eds Celesia G. G. Hickok G. (Amsterdam: Elsevier Science), 55–72.

[B205] Liu F. Jiang C. Pfordresher P. Q. Mantell J. T. Xu Y. Yang Y.. (2013). Individuals with congenital amusia imitate pitches more accurately in singing than in speaking: implications for music and language processing. Attent. Percept. Psychophys. 75, 1783–1798. 10.3758/s13414-013-0506-1 23877539

[B206] Liu L. Lee G.-A. Jiang L. Zhang J. (2007). The earliest rice domestication in China. Antiquity 81, 4–7. Available online at: http://www.antiquity.ac.uk/projgall/liu1/

[B207] Lomax A. (1978). Folk-song Style and Culture. American A. New Brunswick, NJ: Transaction.

[B208] Lukyanov V. (1977). Asafyev's intonation theory and its elaboration in soviet theoretic works [Учение Об Интонации Б. В. Асафьева И Его Разработка В Советской Теоретической Литературе], in Music in the Socialist Society [Музыка В Социалистическом Обществе], Vol. 3, ed A. Farbshtein (Leningrad: Muzyka [Музыка]), 192–219.

[B209] Lundholm H. (1921). The affective tone of lines: experimental researches. Psychol. Rev. 28, 43–60.

[B210] Lu-Ting H. Kuo-huang H. (1982). On chinese scales and national modes. Asian Music 14, 132–154.

[B211] Maceda J. (1990). In search of a source of pentatonic hemitonic and anhemitonic scales in Southeast Asia. Acta Musicol. 62, 192–223.

[B212] Maclarnon A. Hewitt G. (2004). Increased breathing control: another factor in the evolution of human language. Evol. Anthropol. 13, 181–197. 10.1002/evan.20032

[B213] Maher T. F. Berlyne D. E. (1982). Verbal and exploratory responses to melodic musical intervals. Psychol. Music 10, 11–27.

[B214] Mampe B. Friederici A. D. Christophe A. Wermke K. (2009). Newborns' cry melody is shaped by their native language. Curr. Biol. 19, 1994–1997. 10.1016/j.cub.2009.09.064 19896378

[B215] Mang E. (2000). Intermediate vocalizations: an investigation of the boundary between speech and songs in young children's vocalizations. Bull. Counc. Res. Music Educ. 147, 116–121. Available online at: http://www.jstor.org/stable/40319398

[B216] Margulis E. H. (2005). A model of melodic expectation. Music Percept. 22, 663–714. 10.4236/jsea.2010.37078

[B217] Marks L. E. (1978). The Unity of the Senses: Interrelations Among the Modalities. New York, NY: AcademicPress.

[B218] Marušić D. (2007). Reception of istrian musical traditions. Collected Work [Музикологиjа: Часопис Музиколошког Института Српске Академиjе Наука И Уметности]. 7, 185–198. Available online at: http://www.doiserbia.nb.rs/img/doi/1450-9814/2007/1450-98140707185M.pdf

[B219] Masataka N. Perlovsky L. (2012). The efficacy of musical emotions provoked by Mozart's music for the reconciliation of cognitive dissonance. Sci. Rep. 2:694. 10.1038/srep00694 23012648 PMC3457076

[B220] Mathy F. Feldman J. (2012). What's magic about magic numbers? Chunking and data compression in short-term memory. Cognition 122, 346–362. 10.1016/j.cognition.2011.11.003 22176752

[B221] Mazel A. (2011). Time, color, and sound: revisiting the rock art of didima gorge, South Africa. Time Mind 4, 283–296. 10.2752/175169711X13046099195474

[B222] Mazel L. (1952). On Melody [О Мелодии]. Moscow: Gos Muz Izdat [Гос. музыкальное изд-во].

[B223] Mazel L. (1982). On certain aspects of Asafyev's concept [О Некоторых Сторонах Концепции Б. В. Асафьева], in Essays on Theory and Analysis of Music [Статьи По Теории И Анализу Музыки], ed I. Prudnikova (Moscow: Sovetskii Kompozitor [Советский композитор]), 277–307.

[B224] McCune L. Vihman M. M. Roug-Hellichius L. Delery D. B. Gogate L. (1996). Grunt Communication in human infants (Homo Sapiens). J. Comp. Psychol. 110, 211. 10.1037/0735-7036.110.1.27 8851550

[B225] McDermott J. H. Keebler M. V. Micheyl C. Oxenham A. J. (2010a). Musical intervals and relative pitch: frequency resolution, not interval resolution, is special. J. Acoust. Soc. Am. 128, 1943–1951. 10.1121/1.3478785 20968366 PMC2981111

[B226] McDermott J. H. Lehr A. J. Oxenham A. J. (2008). Is relative pitch specific to pitch? Psychol. Sci. 19, 1263–1271. 10.1111/j.1467-9280.2008.02235.x 19121136 PMC2841133

[B227] McDermott J. H. Lehr A. J. Oxenham A. J. (2010b). Individual differences reveal the basis of consonance. Curr. Biol. 20, 1035–1041. 10.1016/j.cub.2010.04.019 20493704 PMC2885564

[B228] McKernon P. E. (1979). The development of first songs in young children. New Dir. Child Adolesc. Dev. 3, 43–58. 10.1002/cd.23219790306

[B229] McLachlan N. Marco D. Light M. Wilson S. J. (2013). Consonance and pitch. J. Exp. Psychol. Gen. 142, 1142–1158. 10.1037/a0030830 23294344

[B230] McLean M. (1991). The Structure of Tikopia Music. Vol. Occasional. Auckland: University of Auckland.

[B231] McNeill W. H. (2008). Keeping Together in Time: Dance and Drill in Human History. New York, NY: ACLS Humanities E-Book.

[B232] McQuere G. D. (ed.). (1983). Boris Asafiev and musical form as a process, in Russian Theoretical Thought in Music (Rochester: University of Rochester Press), 217–252.

[B233] Mercado E. I. I. I. Mantell J. T. Pfordresher P. Q. (2014). Imitating sounds: a cognitive approach to understanding vocal imitation. Comp. Cogn.Behav. Rev. 9, 1–57. 10.3819/ccbr.2014.90002

[B234] Merker B. (2000). Synchronous chorusing and human origins. Musicae Scientiae 3, 59–73. 10.1177/10298649000030S105

[B235] Merriam A. P. (1964). The Anthropology of Music. Evanston, IL: Northwestern University Press.

[B236] Merrill J. Sammler D. Bangert M. Goldhahn D. Lohmann G. Turner R.. (2012). Perception of words and pitch patterns in song and speech. Front. Psychol. 3:76. 10.3389/fpsyg.2012.00076 22457659 PMC3307374

[B237] Messner G. F. (1981). The two-part vocal style on Baluan Island Manus province, Papua New Guinea. Ethnomusicology 25, 433–446. 10.2307/851553

[B238] Messner G. F. (2006). Multipart Vocal Tradition in Eastern Flores (Indonesia), Bulgaria and Manus Province, in Proceedings: The Third International Symposium on Traditional Polyphony, ed R. Tsurtsumia (Tbilisi), 25–29

[B239] Miller G. A. (1994). The magical number seven, plus or minus two: some limits on our capacity for processing information. 1956. Psychol. Rev. 101, 343–352. 10.1037/0033-295X.101.2.343 8022966

[B240] Mithen S. J. (2005). The Singing Neanderthals: The Origins of Music, Language, Mind, and Body. Cambridge, MA: Harvard University Press.

[B241] Morley I. (2013). The Prehistory of Music: Human Evolution, Archaeology, and the Origins of Musicality. Oxford: Oxford University Press.

[B242] Morley I. (2014). A multi-disciplinary approach to the origins of music: perspectives from anthropology, archaeology, cognition and behaviour. J. Anthropol. Sci. 92, 147–177. 10.4436/JASS.92008 25020016

[B243] Morris M. W. Leung K. (2010). Creativity east and west: perspectives and parallels. Manage. Organ. Rev. 6, 313–327. 10.1111/j.1740-8784.2010.00193.x

[B244] Nam U. (1998). Pitch distributions in korean court music: evidence consistent with tonal hierarchies. Music Percept. 16, 243–247. 10.2307/40285789

[B245] Nattiez J.-J. (1976). Canada: Inuit Games and Songs. Paris: Audivis-UNESCO. D 8032.

[B246] Nazajkinskij E. V. (1972). On Psychology of Human Perception [О Психологии Музыкального Восприятия]. Moscow: Muzyka.

[B247] Nettl B. (1953). Stylistic variety in North American Indian music. J. Am. Musicol. Soc. 6, 160–168. 10.2307/829796

[B248] Nettl B. (1956). Infant musical development and primitive music. Southwest. J. Anthropol. 12, 87–91.

[B249] Nettl B. (2000). An ethnomusicologist contemplates universals in musical sound and musical culture, in The Origins of Music, eds Wallin N. L. Merker B. Brown S. (Cambridge, MA: MIT Press), 463–472.

[B250] Nettl B. (2005). The Study of Ethnomusicology: Thirty-one Issues and Concepts. Champaign, IL: University of IllinoisPress.

[B251] Nettl B. (2010). Nettl's Elephant: On the History of Ethnomusicology. Champaign, IL: University of IllinoisPress.

[B252] Neuhaus C. (2013). The perception of melodies: some thoughts on listening style, relational thinking, and musical structure, in Sound - Perception - Performance, Current Research in Systematic Musicology ed Rolf Bader (Heidelberg: Springer International Publishing), 195–215.

[B253] Nguyen P. T. (1986). Restructuring the fixed pitches of the vietnamese dan nguyet lute: a modification necessitated by the modal system. Asian Music 18, 56–70. 10.2307/834158

[B254] Novik Y. (2004). Rite and Folklore in Siberian Shamanism: An Experiment in Correlation of Structures [Обряд И Фольклор В Сибирском Шаманизме: Опыт Сопоставления Структур]. Moscow: Eastern Literature, Russian Academy of Science [Восточная литература РАН].

[B255] Ojamaa T. (2002). The Story of Life in Music: Autobiographical Songs of the Nganasans. Folklore: Electronic Journal of Folklore.

[B256] Ojamaa T. (2003). Composition principles in forest nenets music. Stud. Musicol. Acad. Sci. Hung. 44, 249–256. 10.1556/smus.44.2003.1-2.21

[B257] Ojamaa T. (2005). Throat rasping: problems of visualization. World Music 47, 55–69. 10.2307/41699645

[B258] Ojamaa T. Ross J. (2004). Relationship between texts and tunes in the Siberian Folksongs, in CIM04: Conference on Interdisciplinary Musicology, eds Parncutt R. Kessler A. Zimmer F. (Graz: Graz University), 134–135.

[B259] Ojamaa T. Ross J. (2011). The perceived structure of forest nenets songs: a cross-cultural case study. Psychomusicology 21, 159–175. 10.1037/h0094010

[B260] Oller D. K. (2000). The Emergence of the Speech Capacity. 1st Edn. Mahwah, NJ: Psychology Press.

[B261] Orlova Y. (1984). Intonation Theory of Asafyev as a Theory of Specific Musical Thinking: History, Genesis, Essence [Интонационная Теория Асафьева Как Учение О Специфике Музыкального Мышления: История, Становление, Сущность]. Moscow: Muzyka [Музыка].

[B262] Papoušek H. (1996). Musicality in infancy research: biological and cultural origins of early musicality, in Musical Beginnings: Origins and Development of Musical Competence, ed I. Deliège (New York, NY: Oxford University Press), 37–55.

[B263] Parker S. T. Jaffe K. E. (2008). Darwin's Legacy: Scenarios in Human Evolution. Lanham, MD: Rowman Altamira.

[B264] Perlovsky L. (2012). Cognitive function of music. Part I. Interdiscip. Sci. Rev. 37, 131–144. 10.1179/0308018812Z.00000000010

[B265] Perlovsky L. (2014). The cognitive function of music. Part II. Interdiscip. Sci. Rev. 39, 162–186. 10.1179/0308018813Z.00000000041

[B266] Pettitt P. (2008). Art and the middle-to-upper paleolithic transition in Europe: comments on the archaeological arguments for an early upper paleolithic antiquity of the grotte chauvet art. J. Hum. Evol. 55, 908–917. 10.1016/j.jhevol.2008.04.003 18678392

[B267] Pian R. C. Yung B. Lam J. S. C. (1994). Themes and Variations: Writings on Music in Honor of Rulan Chao Pian. Vol. 1 Cambridge, MA: Department of Music, Harvard University.

[B268] Pierce J. R. (1992). The Science of Musical Sound. New York, NY: Freeman.

[B269] Pisani M. (2008). Imagining Native America in Music. New Haven, CT: Yale University Press.

[B270] Plack C. J. Barker D. Hall D. A. (2014). Pitch coding and pitch processing in the human brain. Hear. Res. 307, 53–64. 10.1016/j.heares.2013.07.020 23938209

[B271] Plack C. J. Watkinson R. K. (2010). Perceived continuity and pitch shifts for complex tones with unresolved harmonics. J. Acoust. Soc. Am. 128, 1922–1929. 10.1121/1.3479757 20968364

[B272] Poffenberger A. T. Barrows B. E. (1924). The feeling value of lines. J. Appl. Psychol. 8, 187–205. 10.1037/h0073513

[B273] Pont G. (2008). Plato's philosophy of dance, in Dance, Spectacle, and the Body Politick, 1250–1750, ed J. Nevile (Bloomington, IN: Indiana University Press), 267–281.

[B274] Povel D. J. Jansen E. (2002). Harmonic factors in the perception of tonal melodies. Music Percept. 20, 51–85. 10.1525/mp.2002.20.1.51

[B275] Powers H. S. Wiering F. Powers H. S. Wiering F. (2001). Mode, in The New Grove Dictionary of Music and Musicians, eds Sadie S. Tyrrell J. (London: Macmillan Publishers).

[B276] Protopopov S. (1930). Intonations [Intonatsii] in Elements of Construction of Musical Speech [Элементы Строения Музыкальной Речи], Vol. 1., ed B. Yavorskii (Moscow: State Edition, Musical Sector [Госуд. Изд-во Музык. Сектор]), 323–380.

[B277] Qian H. (2008). Talent, creativity and regional economic performance: the case of China. Ann. Reg. Sci. 45, 133–156. 10.1007/s00168-008-0282-3

[B278] Quinn I. Mavromatis P. (2011). Voice-leading prototypes and harmonic function in two chorale corpora, in Mathematics and Computation in Music, Vol. 6726, eds Agon C. Andreatta M. Assayag G. Amiot E. Bresson J. Mandereau J. Lecture Notes in Computer Science (Berlin; Heidelberg: Springer), 230–240.

[B279] Raciuniene-Vyciniene D. (2006). The lithuanian archaic polyphonic chant ‘Sutartine’. Lituanus 52, 26–39.

[B280] Radynova O. Albina K. Marine P. (ed.). (1994). Musical Upbringing of Preschoolers [Mузыкальное Воспитание Дошкольников]. (Moscow: Prosvesheniye).

[B281] Rags Y. (1980). The concept of zonal nature of musical hearing by N. A. Garbuzov [Концепция Зонной Природы Музыкального Слуха Н. А. Гарбузова], in Garbuzov N. A. - Musician, Researcher and Pedagoge [Гарбузов Н. А. - Музыкант, Исследователь, Педагог], ed Y. Rags (Moscow: Muzyka [Музыка]), 11–48.

[B282] Rahn J. Bigand E. Poulin-Charronnat B. (2004). Implicit learning of indian music by westerners, in Proceedings of the Conference on Interdisciplinary Musicology (CIM04) (Graz: Graz University), 142–143.

[B283] Rakowski A. (1993). Categorical perception in absolute pitch. Arch. Acoust. 18, 515–523.

[B284] Rakowski A. (2009). The domain of pitch in music. Arch. Acoust. 443, 429–443. Available online at: http://acoustics.ippt.gov.pl/index.php/aa/article/view/586/517

[B285] Rappoport D. (2011). The enigma of alternating duets in flores and solor, in Tradition, Identity, and History-Making in Eastern Indonesia, ed Hans H¨agerdal (Kalmar: Linnaeus University Press), 130–148.

[B286] Reigado J. Rocha A. Rodrigues H. (2011). Vocalizations of infants (9–11 months old) in response to musical and linguistic stimuli. Int. J. Music Educ. 29, 241–255. 10.1177/0255761411408507

[B287] Reinhard K. (1958). On the problem of pre-pentatonic scales: particularly the third-second nucleus. J. Int. Folk Music Counc. 10, 15–17. 10.2307/835966

[B288] Reznikoff I. (2004). On primitive elements of musical meaning. JMM 3 (Section 2). Available online at: http://www.musicandmeaning.net/issues/showArticle.php?artID=3.2],sec.2.1.

[B289] Reznikoff I. (2008). Sound resonance in prehistoric times: a study of paleolithic painted caves and rocks. J. Acoust. Soc. Am. 123, 3603 10.1121/1.2934773

[B290] Rhodes W. (1949). Music of the Sioux and the Navajo. New York, NY: Folkways Records. FW04401/FE 4401.

[B291] Ross D. A. Gore J. C. Marks L. E. (2005). Absolute pitch: music and beyond. Epilepsy Behav. 7, 578–601. 10.1016/j.yebeh.2005.05.019 16103017

[B292] Rothfarb L. A. (1988). Ernst Kurth as Theorist and Analyst. Philadelphia, PA: University of Pennsylvania Press.

[B293] Rowell L. (1981). Early Indian musical speculation and the theory of melody. J. Music Theory 25, 217–244. 10.2307/843650

[B294] Rowell L. (2000). Scale and mode in the music of the early tamils of South India. Music Theory Spectr. 22, 135–156. 10.2307/745957

[B295] Rubtsov F. (1962). Intonational Connections in Art of Singing of Slavic Ethnicities [Интонационные Связи В Песенном Творчестве Славянских Народов: Опыт Исследования]. Leningrad: Soviet Composer [Советский композитор].

[B296] Rubtsov F. (1973). The foundations of modal morphology of russian traditional songs [Основы Ладового Строения Русских Народных Песен], in Essays on Musical Folklore [Статьи По Музыкальному Фольклору], ed M. A. Dunayevskii (Moscow: Sovetskii Kompozitor [Сов. композитор]), 8–81.

[B297] Rudneva A. (1994). Russian Traditional Musical Works: Essays on the Theory of Folklore [Русское Народное Музыкальное Творчество: Очерки По Теории Фольклора]. Moscow: Kompozitor [Композитор].

[B298] Rudowicz E. (2004). Creativity among Chinese people: beyond western perspective, in Creativity: When East Meets West, eds Lau S. Hui A. H. H. Ng G. Y. C. (New Jersey, NJ, London: World Scientific), 55–86.

[B299] Rutkowski J. (1997). The nature of children's singing voices: characteristics and assessment, in The Phenomenon of Singing, ed B. A. Roberts (St. John's, NF: Memorial University Press), 201–209.

[B300] Sachs C. (1960). Primitive and medieval music: a parallel. J. Am. Musicol. Soc. 13, 43–49. 10.2307/830245

[B301] Sachs C. (1962). The Wellsprings of Music, ed J. Kunst. The Hague: Martinus Nijhoff.

[B302] Sachs C. (2008). The Rise of Music in the Ancient World, East and West. New York, NY: Dover Publications.

[B303] Sammler D. Baird A. Valabrègue R. Clément S. Dupont S. Belin P.. (2010). The relationship of lyrics and tunes in the processing of unfamiliar songs: a functional magnetic resonance adaptation study. J. Neurosci. 30, 3572–3578. 10.1523/JNEUROSCI.2751-09.2010 20219991 PMC6632242

[B304] Schellenberg E. G. Trehub S. E. (1996). Children's discrimination of melodic intervals. Dev. Psychol. 32, 1039–1050. 10.1037/0012-1649.32.6.1039

[B305] Schneider A. (2001). Sound, pitch, and scale: from ‘Tone Measurements’ to sonological analysis in ethnomusicology. Ethnomusicology 45, 489–519. 10.2307/852868

[B306] Schneider A. (2006). Comparative and systematic musicology in relation to ethnomusicology: a historical and methodological survey. Ethnomusicology 50, 236–258. Available online at: http://www.jstor.org/stable/20174451

[B307] Schneider A. (2010). Music theory: speculation, reasoning, experience—a perspective from systematic musicology, in Zeitschrift Der Gesellschaft Für Musiktheorie, Vol. 7, eds Janz T. Sprick J. Ph. (Hildesheim; New York, NY: Olms), 53–97.

[B308] Schneider A. (2013). Change and continuity in sound analysis: a review of concepts in regard to musical acoustics, music perception, and transcription, in Sound - Perception - Performance, ed R. Bader (Berlin: Springer), 71–111. 10.1007/978-3-319-00107-4_3

[B309] Seguin-Orlando A. Korneliussen T. S. Sikora M. Malaspinas A.-S. Manica A. Moltke I.. (2014). Genomic structure in Europeans dating back at least 36,200 years. Science 346, 1113–1118. 10.1126/science.aaa0114 25378462

[B310] Sevåg R. (1974). Neutral tones and the problem of mode in Norwegian folk music, in Studia Instrumentorum Musicae Popularis III: Festschrift to Ernst Emsheimer on the Occasion of His 70th Birthday, January 15th 1974, ed G. Hillestr¨om (Stockholm: Musikhistoriska Museet), 207–213.

[B311] Shakhnazarova N. (1966). Intonational Glossary and the Problem of Folkishness [Интонационный Словарь И Проблема Народности]. Moscow: Muzyka [Музыка] Available online at: http://books.google.com/books?id=rUEUZH6iZaoC

[B312] Sheikin Y. (2002). History of Music Culture of Siberia Peoples: Comparativ-Historic Investigation [История музыкальной культуры народов Сибири: сравнительно-историческое исследование]. Moscow: Eastern Literature, Russian Academy of Science [Восточная литература РАН].

[B313] Shepard R. N. (2010). One cognitive psychologist's quest for the structural grounds of music cognition. Empir. Musicol. Rev. 20, 130–157. 10.1037/h0094217

[B314] Shrivastav R. Eddins D. A. Anand S. (2012). Pitch strength of normal and dysphonic voices. J. Acoust. Soc. Am. 131, 2261–2269. . 10.1121/1.3681937 22423721 PMC3316683

[B315] Shumays S. A. (2013). Maqam analysis: a primer. Music Theory Spectr. 35, 235–255. 10.1525/mts.2013.35.2.23517553831

[B316] Sipos J. (2005). Comparative Research on the Folk Music of Turkic and Hungarian People. Ankara: Macaristan Cumhuriyeti Ankara B¨uy¨ukelçiligi'nin Yayını.

[B317] Skrebkov S. (1967). Intonation and mode [Интонация И Лад]. Sovetskaya Muzyka 1, 89–94.

[B318] Skrebkov S. (1973). Artistic Principles behind Musical Styles [Художественные Принципы Музыкальных Стилей]. Moscow: Muzyka [Музыка].

[B319] Smith J. D. (1997). The place of musical novices in music science. Music Percept. 14, 227–262. 10.2307/40285720

[B320] Smith J. D, Kemler Nelson, D. G. Grohskopf L. A. Appleton T. (1994). What child is this? What interval was that? Familiar tunes and music perception in novice listeners. Cognition 52, 23–54. 10.1016/0010-0277(94)90003-5 7924198

[B321] Staun J. (2013). Fission in component-based phonology. Lang. Sci. 40, 123–147. 10.1016/j.langsci.2013.03.006

[B322] Stefanics G. Háden G. P. Sziller I. Balázs L. Beke A. Winkler I. (2009). Newborn infants process pitch intervals. Clin. Neurophysiol. 120, 304–308. 10.1016/j.clinph.2008.11.020 19131275

[B323] Sundberg J. (1992). Musician's tone glue. Q. Prog. Status Rep. 33, 79–98.

[B324] Sychenko G. (2009). Shamanic intonation': history and phenomenology of the concept, in Perspectives on the Song of the Indigenous Peoples of Northern Eurasia: Performance, Genres, Musical Syntax, Sound, ed J. Niemi (Tampere: Tampere University Press), 58–72.

[B325] Szabolcsi B. (1943). Five-tone scales and civilization. Acta Musicol. 15, 24–34. 10.2307/932058

[B326] Szabolcsi B. (1965). History of Melody. Budapest: Barrie and Rockliff.

[B327] Tarr B. Launay J. Dunbar R. (2014). Music and social bonding: ‘Self-Other’ merging and neurohormonal mechanisms. Front. Psychol. 5:1096. 10.3389/fpsyg.2014.01096 25324805 PMC4179700

[B328] Temperley D. Marvin E. W. (2008). Pitch-class distribution and the identification of key. Music Percept. 25, 193–212. 10.1525/mp.2008.25.3.193

[B329] Teplov B. (1947). The Psychology of Musical Abilities [Психология Музыкальных Способностей]. Moscow: Academy of Pedagogical Sciences of Russia.

[B330] Terhardt E. (1974). Pitch, consonance, and harmony. J. Acoust. Soc. Am. 55, 1061–1069. 10.1121/1.1914648 4833699

[B331] Terhardt E. (1987). Some psycho-physical analogies between speech and music, in Musik in Der Medizin/Music in Medicine, eds Spintge R. Droh R. (Berlin: Springer Berlin Heidelberg), 107–118. 10.1007/978-3-642-71697-3_8

[B332] Thompson W. F. (2004). From sounds to music: the contextualizations of pitch. Music Percept. 21, 431–456. 10.1525/mp.2004.21.3.431

[B333] Tiulin Y. (1966). Teaching of Harmony [Учение О Гармонии]. 3rd Edn. Moscow: Muzyka [Музыка].

[B334] Toiviainen P. Luck G. Thompson M. R. (2010). Embodied meter: hierarchical eigenmodes in music-induced movement. Music Percept. 28, 59–70. 10.1525/mp.2010.28.1.59

[B335] Trainor L. J. (2007). Do preferred beat rate and entrainment to the beat have a common origin in movement? Empir. Musicol. Rev. 2, 17–20. Available online at: http://hdl.handle.net/1811/24480

[B336] Trainor L. J. Gao X. Lei J.-J. Lehtovaara K. Harris L. R. (2009). The primal role of the vestibular system in determining musical rhythm. Cortex 45, 35–43. 10.1016/j.cortex.2007.10.014 19054504

[B337] Trujillo L. T. Jankowitsch J. M. Langlois J. H. (2014). Beauty is in the ease of the beholding: a neurophysiological test of the averageness theory of facial attractiveness. Cogn. Affect. Behav. Neurosci. 14, 1061–1076. 10.3758/s13415-013-0230-2 24326966 PMC4053512

[B338] Tull J. R. Asafyev B. (2000). B. V. Asaf'ev's Musical Form as a Process: Translation and Commentary (transl. J. R. Tull, Photocopy: Vol. 3). Ann Arbor: University Microfilms International.

[B339] van Noorden L. P. (1975). Temporal Coherence in the Perception of Tone Sequences. Vol. 3 Eindhoven; Holland: Institute for Perceptual Research.

[B340] van Oost P. (1912). Chansons populaires chinoises de La région sud des ortos. Anthropos 7, 161–193.

[B341] Van Puyvelde M. Vanfleteren P. Loots G. Deschuyffeleer S. Vinck B. Jacquet W.. (2010). Tonal synchrony in mother-infant interaction based on harmonic and pentatonic series. Infant Behav. Develop. 33, 387–400. 10.1016/j.infbeh.2010.04.003 20478620

[B342] van Zanten W. (2004). Perception of Sundanese music: an experimental approach, in 37th World Conference of the International Council for Traditional Music, ed D. Niles (Fuzhou: International Council for Traditional Music), 278-279.

[B343] Vega D. (2003). A perceptual experiment on harmonic tension and melodic attraction in Lerdahl's Tonal pitch space. Musicae Scientiae 7, 35–55. 10.1177/102986490300700103

[B344] Vestergaard M. D. Háden G. P. Shtyrov Y. Patterson R. D. Pulverm¨uller F. Denham S. L.. (2009). Auditory size-deviant detection in adults and newborn infants. Biol. Psychol. 82, 169–175. 10.1016/j.biopsycho.2009.07.004 19596043 PMC2829091

[B345] von Hornbostel E. (1975). Tunesian melodies recorded on the phonograph in Opera Omnia, Vol. 1 eds Wachsmann K. P. Christensen D. Reinecke H.-P. (The Hague: Martinus Nijhoff), 117–155.

[B346] von Hornbostel E. M. (1928). African Negro music. Africa 1, 30–62. 10.2307/1155862

[B347] Von Hornbostel E. M. (1948). The music of the fuegians. Ethnos 13, 61–102. 10.1080/00141844.1948.9980678

[B348] Vos P. G. Troost J. M. (1989). Ascending and descending melodic intervals: statistical findings and their perceptual relevance. Music Percept. 6, 383–396. 10.2307/40285439

[B349] Walker R. (1997). Visual metaphors as music notation for sung vowel spectra in different cultures. J. New Music Res. 26, 315–345. 10.1080/09298219708570733

[B350] Waller S. J. (2006). Intentionality of rock-art placement deduced from acoustical measurements and echo myths, in Archaeoacoustics: The Archaeology of Sound: Publication of Proceedings from the 2014 Conference in Malta, eds Scarre C. Kemp D. A. (Cambridge, UK: McDonald Institute for Archaeological Research University of Cambridge), 31–40.

[B351] Wallin N. L. (1991). Biomusicology: Neurophysiological, Neuropsychological, and Evolutionary Perspectives on the Origins and Purposes of Music. New York, NY: Pendragon Press.

[B352] Wallin N. L. Merker B. (2001). The Origins of Music. Cambridge, MA: MITPress.

[B353] Weick K. E. Putnam T. (2006). Organizing for mindfulness: eastern wisdom and western knowledge. J. Manag. Inq. 15, 275–287. 10.1177/1056492606291202

[B354] Welch G. F. (2006). Singing and vocal development, in The Child as Musician: A Handbook of Musical Development, ed G. McPherson (New York, NY; London: Oxford University Press), 311–330. 10.1093/acprof:oso/9780198530329.003.0016

[B355] Wermke K. Mende W. (2009). Musical elements in human infants' cries: in the beginning is the melody. Music. Sci. 13(2 Suppl.), 151–175. 10.1177/1029864909013002081

[B356] West M. L. (1981). The singing of Homer and the modes of early Greek music. J. Hell. Stud. 101, 113–129. 10.2307/629848

[B357] West M. L. (1992). Ancient Greek Music. New York, NY, London: Oxford University Press.

[B358] Wiggins G. A. Mullensiefen D. Pearce M. T. (2010). On the non-existence of music: why music theory is a figment of the imagination. Musicae Scientiae 14(1 Suppl.), 231–255. 10.1177/10298649100140S110

[B359] Wilbanks W. A. Pate M. W. (1979). Discrimination of melodies from the first and fifth serials of the pentatonic scale. Bull. Psychon. Soc. 13, 81–84. 10.3758/BF03335019

[B360] Will U. (1997). Two types of octave relationships in central australian vocal music? Musicol. Aust. 20, 6–14. 10.1080/08145857.1997.10415970

[B361] Wilson E. O. (2000). Sociobiology: The New Synthesis. Cambridge, MA: Harvard University Press.

[B362] Winkielman P. Halberstadt J. Fazendeiro T. Catty S. (2006). Prototypes are attractive because they are easy on the mind. Psychol. Sci. 17, 799–806. 10.1111/j.1467-9280.2006.01785.x 16984298

[B363] Winkler I. Kushnerenko E. Horváth J. Ceponiene R. Fellman V. Huotilainen M.. (2003). Newborn infants can organize the auditory world. Proc. Natl. Acad. Sci. U.S.A. 100, 11812–11815. 10.1073/pnas.2031891100 14500903 PMC208846

[B364] Wiora W. (1959). Older than pentatony, in Studia Memoriae Bela Bartok Sacra, eds Rejeczky B. Ter E. (London: Boosey and Hawkes), 183–206.

[B365] Wiora W. (1962). La musique À L'époque de La peinture paléolithique. J. Int. Folk Music Counc. 14, 1–6. 10.2307/835551

[B366] Wulstan D. (1971). The origin of the modes. Stud. Eastern Chant 2, 4–20. 9422255

[B367] Wurz S. (2010). Interpreting the fossil evidence for the evolutionary origins of music. South. Afr. Humanit. 21, 395–417. Available online at: http://www.sahumanities.org/ojs/index.php/SAH/article/viewFile/297/266

[B368] Wynn T. (1985). Piaget, stone tools and the evolution of human intelligence. World Archaeol. 17, 32–43. 10.1080/00438243.1985.9979948 16470990

[B369] Xiang H. Y. Walker P. A. (2014). China Cultural and Creative Industries Reports 2013. Berlin: Springer 10.1007/978-3-642-38157-7

[B370] Yampolsky P. (1995a). Music of Indonesia, Vol. 8: Vocal and Instrumental Music from East and Central Flores. New York, NY: Smithsonian Folkways Recordings. SFW40424.

[B371] Yampolsky P. (1995b). Music of Indonesia, Vol. 9: Music from Central and West Flores. New York, NY: Smithsonian Folkways Recordings. SFW40425.

[B372] Yasser J. (1932). A Theory of Evolving Tonality. New York, NY: American Library of Musicology.

[B373] Yavorskii B. (1908). The Construction of Musical Speech. Materials and Notes [Строение Музыкальной Речи. Материалы И Заметки]. Vol. 1 Moscow: Jurgenson [Юргенсон].

[B374] Yost W. (2009). Pitch perception. Atten. Percept. Psychophys. 71, 1701–1715. 10.3758/APP.71.8.1701 19933556

[B375] Yue X. D. Rudowicz E. (2002). Perception of the most creative Chinese by undergraduates in Beijing, Guangzhou, Hong Kong, and Taipei. J. Creat. Behav. 36, 88–104. 10.1002/j.2162-6057.2002.tb01058.x

[B376] Zarate J. M. Ritson C. R. Poeppel D. (2012). Pitch-interval discrimination and musical expertise: is the semitone a perceptual boundary? J. Acoust. Soc. Am. 132, 984–993. 10.1121/1.4733535 22894219 PMC3427364

[B377] Zarate J. M. Wood S. Zatorre R. J. (2010). Neural networks involved in voluntary and involuntary vocal pitch regulation in experienced singers. Neuropsychologia 48, 607–618. 10.1016/j.neuropsychologia.2009.10.025 19896958

[B378] Zemcovskij I. (2005). Neither east nor west; in between but not a bridge: a riddle for a new discipline, the ethnogeomusicology. Muzikologija: Časopis Muzikološkog Instituta Srpske Akademije Nauka I Umetnosti 5, 195–203. 10.2298/MUZ0505195Z24904524

[B379] Zemp H. (1967). Baule Vocal Music. Paris: Audivis-UNESCO. D 8048.

[B380] Zemtsovsky I. (1980). Asafyev and methodological foundations of intonational analysis of the folk music [Б. В.Асафьев И Методологические Основы Интонационного Анализа Народной Музыки], in Criticism and Musicology [Критика И Музыкознание], Vol. 2, ed O. P. Kolovskii (Leningrad: Muzyka [Музыка]), 184–198.

[B381] Zemtsovsky I. (1983). Song as a historic phenomenon [Песня Как Исторический Феномен], in Popular Song: Problems of Study [Народная Песня. Проблемы Изучения], Vol. 6, ed V. Gusev (Leningrad: Saint Petersburg State Theatre Arts Academy [ЛГИТМИК]), 22–35.

[B382] Zemtsovsky I. (1987). On melodic formula in russian folklore [О Мелодической 'Формульности' В Русском Фольклоре], in Ethnographic Origins of Folkloric Phenomena: Russian Folklore [Этнографические Истоки Фольклорных Явлений. Русский Фольклор], Vol. 14, ed V. Yeremina (Leningrad: Nauka), 117–128.

[B383] Zemtsovsky I. (1998). The melodic system of pentatonism (a sketch about the Mongolian Version), in Ethnologische, Historische Und Systematische Musikwissenschaft: Oskár Elschek Zum 65. Geburtstag, eds F¨odermayr F. Burlas L. (Bratislava: ASCO art and science), 193–195.

[B384] Zemtsovsky I. (2001). V. Ia. Propp and ethnomusicology: memoirs and approaches. Folklorica 6, 26–30. 10.17161/folklorica.v6i1.3701

[B385] Zhang J. Xiao X. Lee Y. K. (2004). The early development of music. analysis of the jiahu bone flutes. Antiquity 302, 769–778. 10.1017/S0003598X00113432

[B386] Zivic P. H. R. Shifres F. Cecchi G. A. (2013). Perceptual basis of evolving western musical styles. Proc. Natl. Acad. Sci. U.S.A. 110, 10034–10038. 10.1073/pnas.1222336110 23716669 PMC3683751

[B387] Zubrow E. B. W. Blake E. C. (2006). The origin of music and rhythm, in Archaeoacoustics, eds Scarre C. Lawson G. (Cambridge: McDonald Institute for Archaeological Research), 142.

