# Supplementary material for: Evolution of tonal organization in music mirrors symbolic representation of perceptual reality. Part-1: Prehistoric
Source: Front Psychol. 2015 Oct 16;6:1405. doi: 10.3389/fpsyg.2015.01405 (PMC4607869; doi:10.3389/fpsyg.2015.01405)
Supplement: Supplementary file 1 [file Presentation1.PDF]

Demonstration 1: Declamation versus Singing.

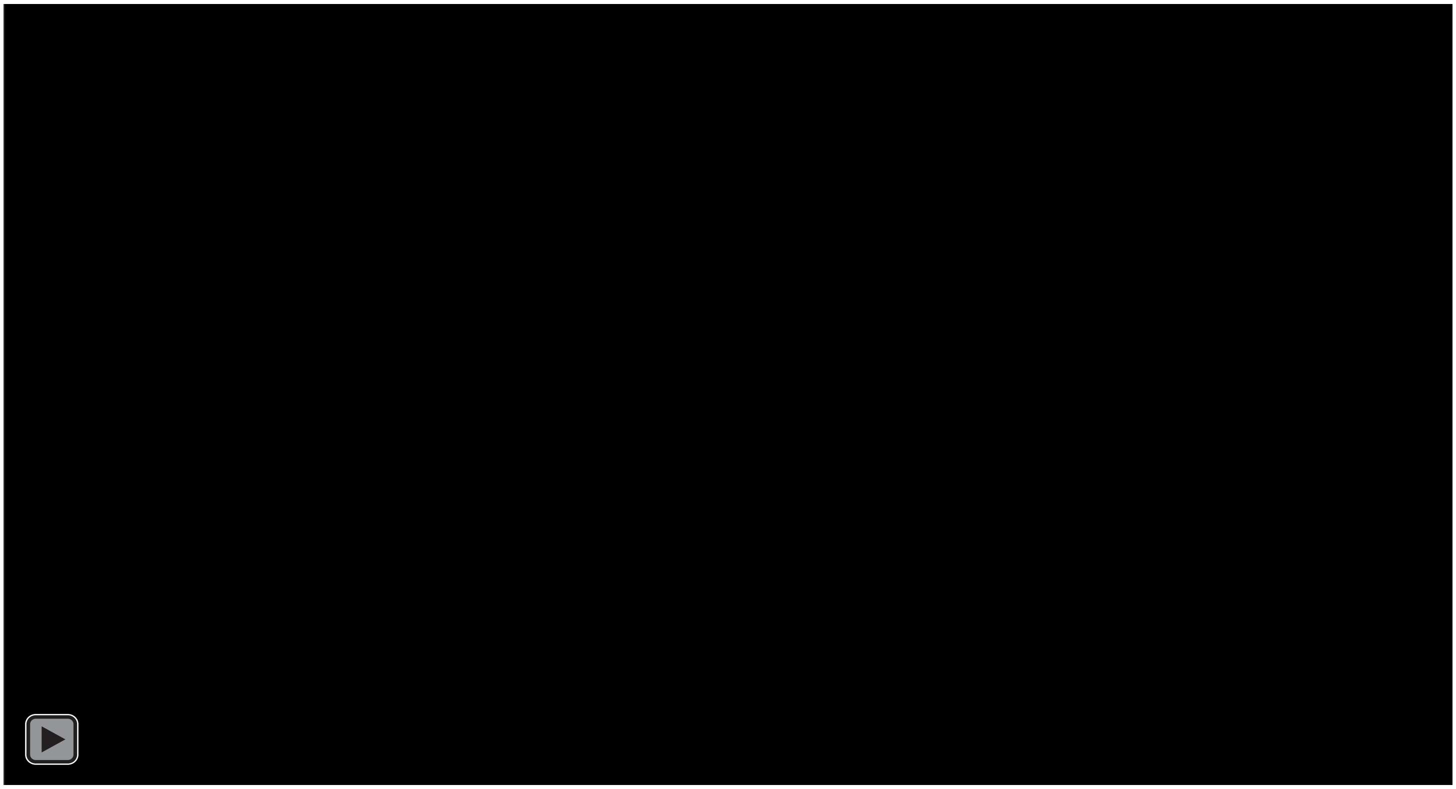

(A) Introductory recitative from the olokho Er Sogotokh. Vasilii Karatayev (1986), from the collection by Eduard Alekseyev, courtesy of Eduard Alekseyev. Used by permission.

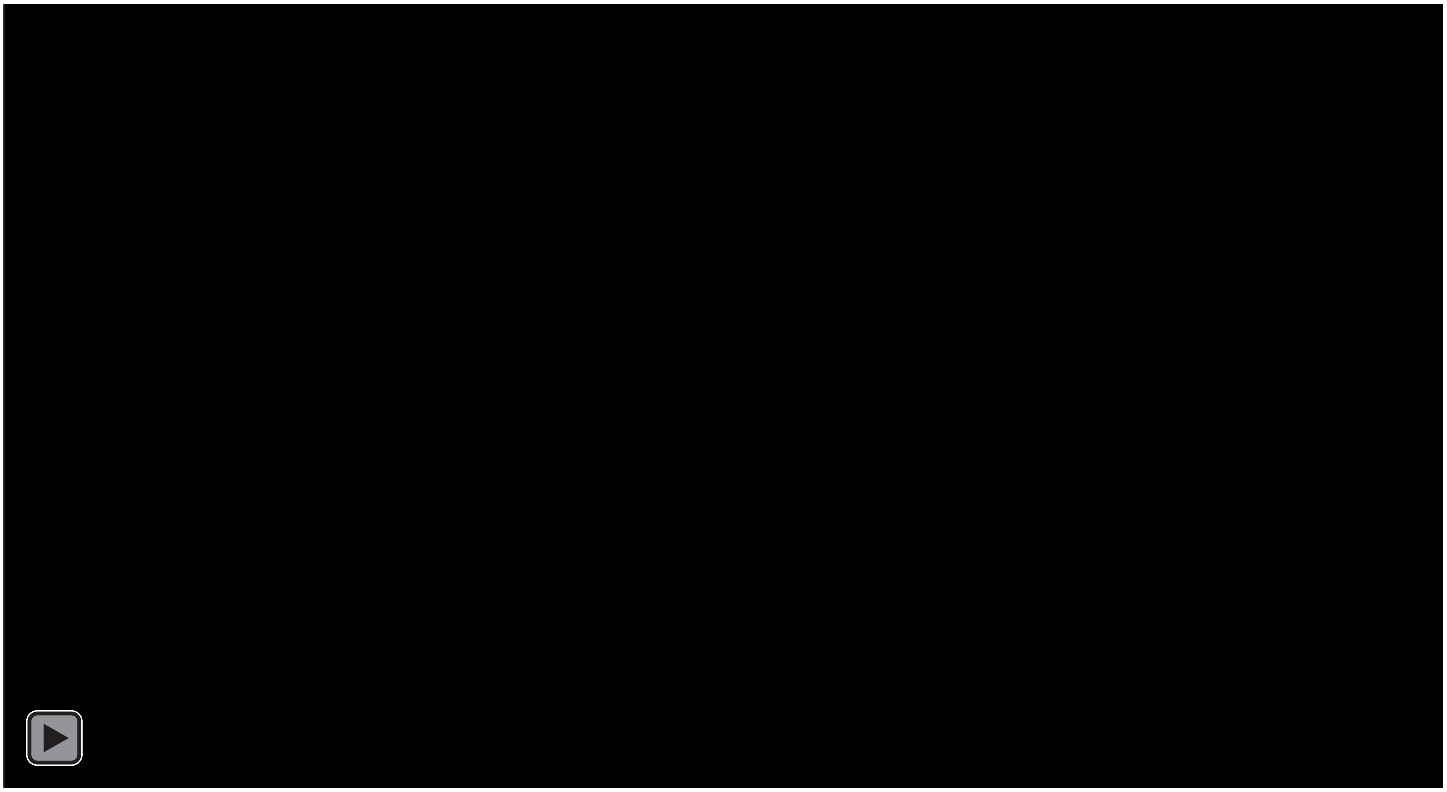

(B) Song of the Underworld Bokhatoor from the olokho Er Sogotokh. Vasilii Karatayev (1986), from the collection by Eduard Alekseyev, courtesy of Eduard Alekseyev. Used by permission.

In both audio samples you can hear the same person recorded at the same recording session. (A) presents declamatory introduction into the epic story of Er Sogotokh, narrated by the singer. It features narrative intonation in a rather free rhythm, but relatively monotonous in pitch, restricted to extremely narrow ambitus between 150.7 Hz (D3+47cents) and 177 Hz (F3+23), which altogether makes 276 cents (little smaller than a minor 3rd).

(B) presents a song of a “negative” character from the underworld. It portrays a scary angry image. The ambitus greatly increases: from 120.3 Hz (B2-42) to 339.7 Hz (F4-45), making 1,797 cents from the lowest to the highest fundamental frequency m more than 6.5 times wider than in the first sample. The pitch contour features khasmatonal leaps, vibratos of different rate, ascending portamento at the starting point of a phrase, and descending glissando at the end of it. The melodic formula, which starts after the initial militaristic call, is characterized by frequent changes in direction.

Equally contrasting is the spectral content: both spectrograms enclose the same span of two octaves (vertical axis). However, the speech sample fits in three formants in this range, whereas the musical sample - only two. Both formants in music remain dynamically active, alternating in their prevalence (louder dynamics is reflected by brighter color). The musical sample (B) contains much greater density of information within the same span of 16 seconds: there are a lot more changes in pitch, amplitude, and spectral content in B) than in A). The comparison between both of them should be taken as a general idea of how proto-music must have related to first samples of music in pre-modal tonal organization.
