# Supplementary material for: Evolution of tonal organization in music mirrors symbolic representation of perceptual reality. Part-1: Prehistoric
Source: Front Psychol. 2015 Oct 16;6:1405. doi: 10.3389/fpsyg.2015.01405 (PMC4607869; doi:10.3389/fpsyg.2015.01405)
Supplement: Supplementary file 2 [file Presentation2.PDF]

Demonstration 2: Centrifugal versus Centripetal Gravity.

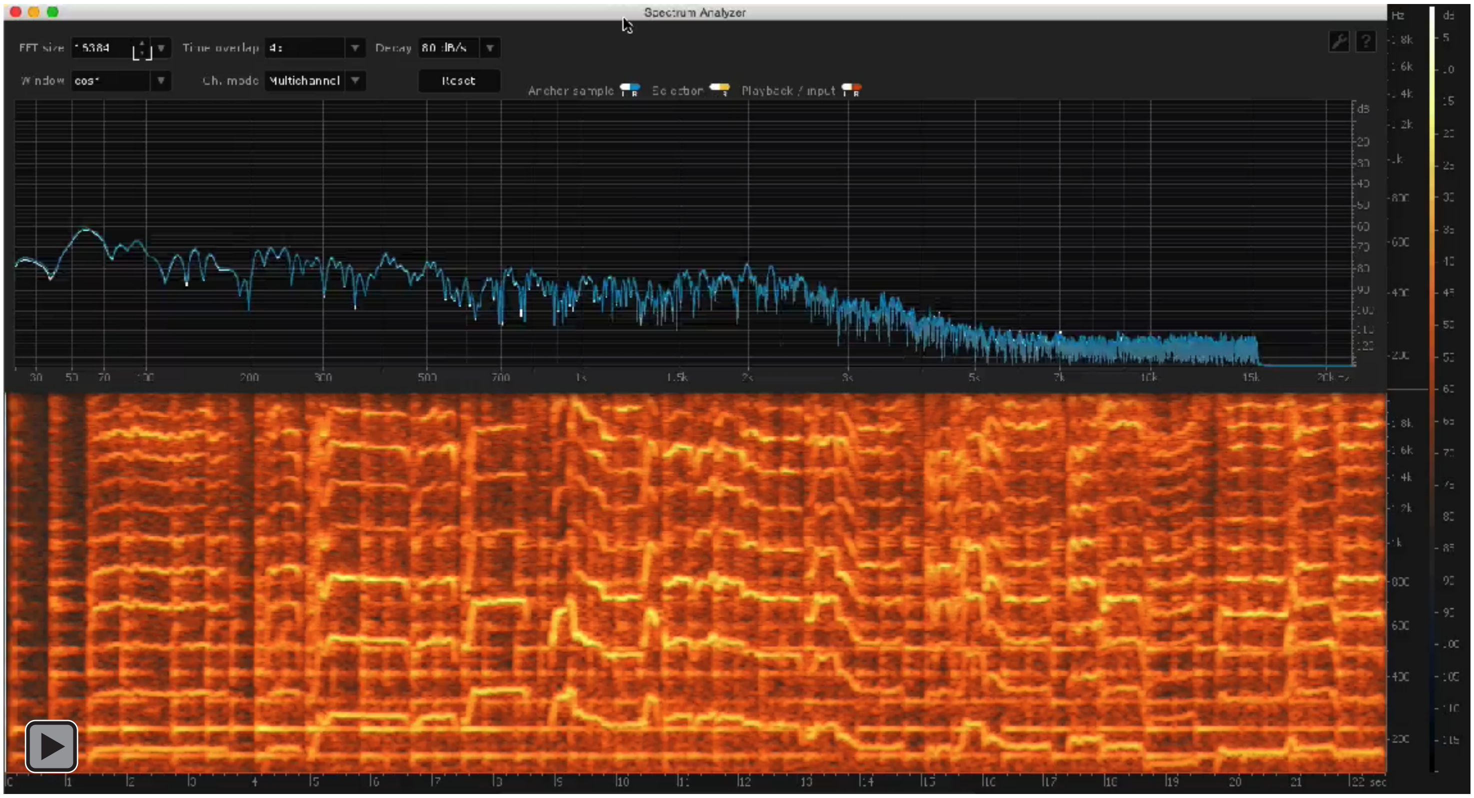

(A) "It was a very lovely day when the water was calm" by Joe Sikvayunak from the recording entitled "Cry from the Earth: Music of the North American Indians," SFW37777, courtesy of Smithsonian Folkways Recordings. (p) (c) 1979. Used by permission.

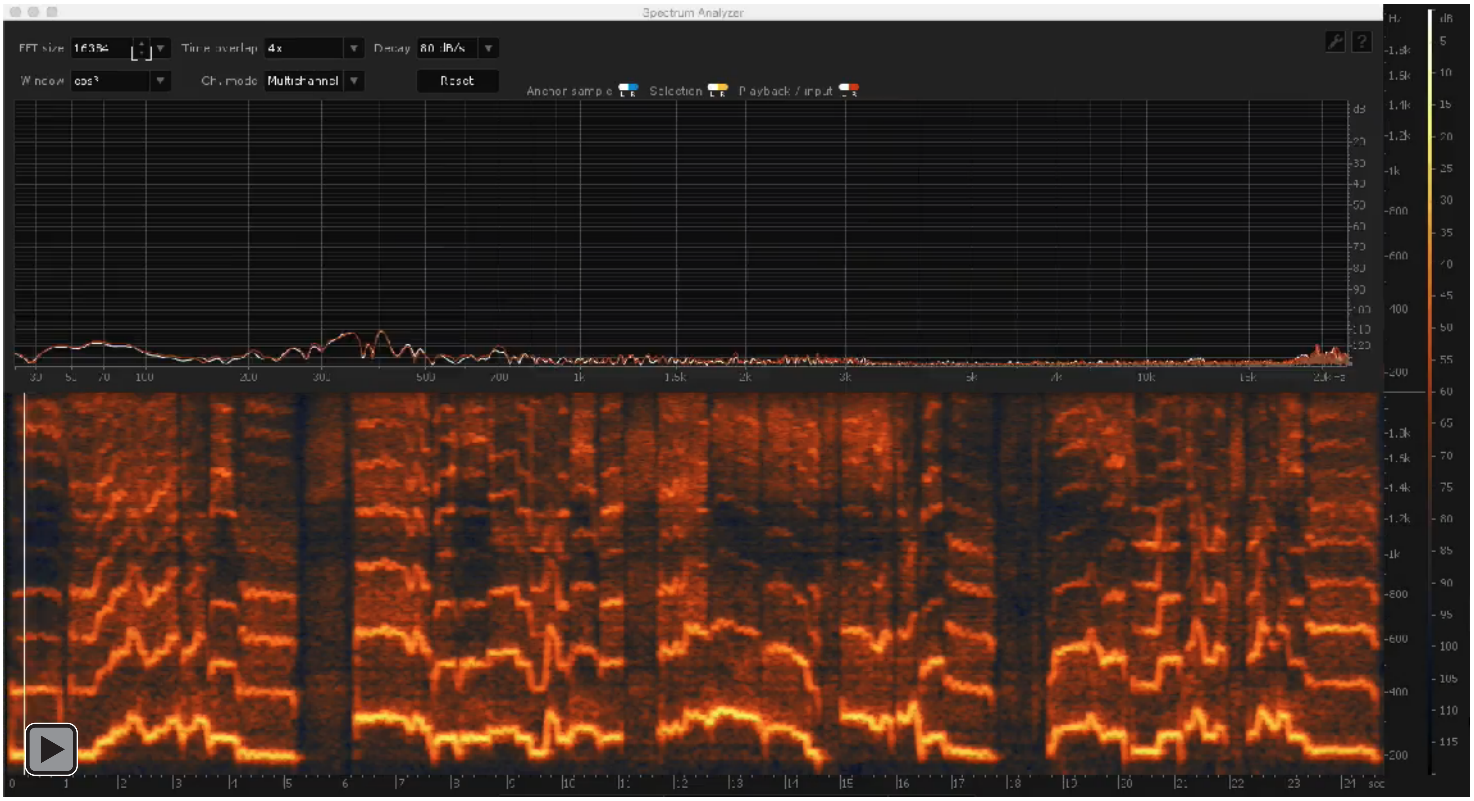

(B) "Ne iz sadu" by Ivan Banderovsky from the recording entitled Old Believers: Songs of the Nekrasov Cossacks, SFW40462, courtesy of Smithsonian Folkways Recordings. (p) (c) 1995. Used by permission.

(A) presents a song where the energy of the melodic intonation exceeds the gravity force of any tone in the PS. The 22 second long melody sample contains the following tones: stressed F3-22, C#4-34, F4+29, then, the passing E4+14, C4+28, B3-28, E4-21, stressed C4+41, B3-28, passing G#-4, stressed F3+26, passing B3-13, G#-43, stressed F3+44, passing G#-46, F#-39, stressed C3+24, and semi-stressed E3-18, F3+18, and then again E3-16. The tones that stay the most stabilized in their frequency values are E3, F3, F#3, B3, C4. Of them extra stress receive E and F because of their reasonably close octave versions. None of these tones demonstrates clear gravitational dominance. Octave equivalent E and F stand out, as well as G# that is stressed by being in the center of multiple leaps and having an auxiliary F#.

Construction of such melody is driven not by the idea of mapping specific tones for better navigation, but by inertia of ascending and descending motion, initiated by leaps. The singer does not seem to care of exact position of his voice in pitch, and is emotionally overwhelmed by happiness. This is an example of a spontaneous personal Inuit dance song. The melody is defined in terms of direction and rough distance of leaps versus passing steps - rather than by sequential order in melodic formula or attraction to a specific tone. Such melody can be characterized as centrifugal in gravity, since the singer is attracted by the idea of reaching the summit of his ambitus, when he is in ascending inclination; and by the idea of dropping to the bottom of his ambitus, when he is in descending inclination.

(B) presents a formula driven epic genre of bylina. Such song can be very long and be built on 1 or 2 formulas varied throughout the performance. The necessity to tell a story, often quite complex in its plot, compels the performer to articulate the text. Music assists in distributing the stress accents in the strophes by breaking new sentences in typological manner, “rhyming” specific words. Complexity of text makes this melody a lot more hierarchic, as compared to the Inuit song. The opening G#3-31 is balanced by G3+23 (46 cents lower) at the end of the antecedent sentence. The longer consequent sentence starts at the “dominant” tone, D#4+7, and resolves back into G#3. Most of the melody “wiggles” on the passing tones situated around the “dominant,” releasing the tension by gradual fall towards the lower “tonic” as the singer runs out of breath.

The formula usually steadily rises in pitch between different verses, accompanying the singer’s growing excitement. However, the gravitational scheme of starting the phrase at the “dominant” after taking a breath, wiggling around it, and collapsing to “tonic” towards the end of the breath pertains throughout the song. Such model presents a definite centripetal scheme of gravity. In this particular example, the lowest tone of the ambitus is the strongest, so the word “centripetal” is not strict in the sense of marking the “center” of the ambitus. The lowest tone is “central” here in a sense of its importance in marking the beginning and the end of big chunks of information. However, in many songs which feature the suprafix a 4th lower than the “tonic,” such a “tonic” indeed becomes the central point in ambitus.
