# Supplementary material for: Evolution of tonal organization in music mirrors symbolic representation of perceptual reality. Part-1: Prehistoric
Source: Front Psychol. 2015 Oct 16;6:1405. doi: 10.3389/fpsyg.2015.01405 (PMC4607869; doi:10.3389/fpsyg.2015.01405)
Supplement: Supplementary file 3 [file Presentation3.PDF]

Demonstration 3: Khasmatonal versus Oligotonal styles.

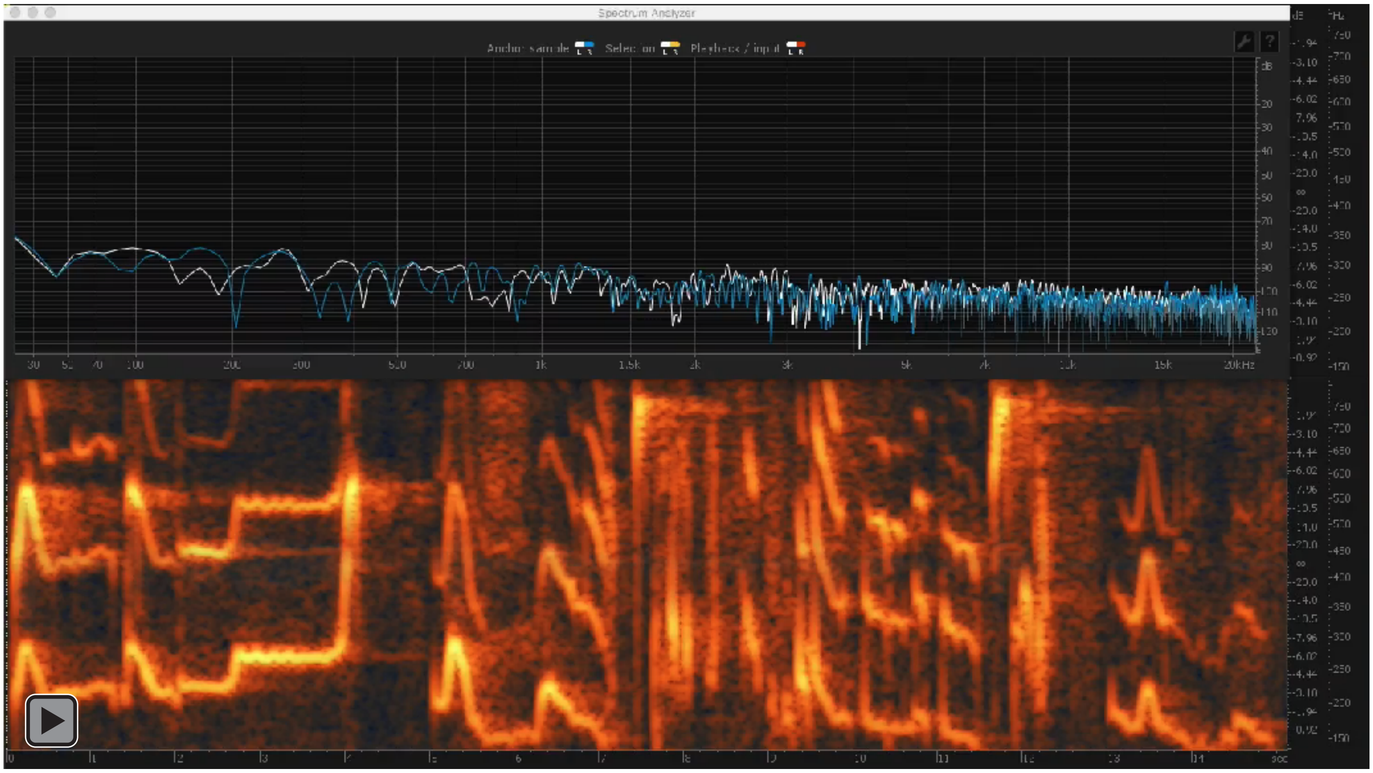

(A) "Lithuanian Lullaby" by Veronika Povilioniene with a grandchild from the recording entitled Smithsonian Folkways World Music Collection, SFW40471, courtesy of Smithsonian Folkways Recordings. (p) (c) 1997. Used by permission.

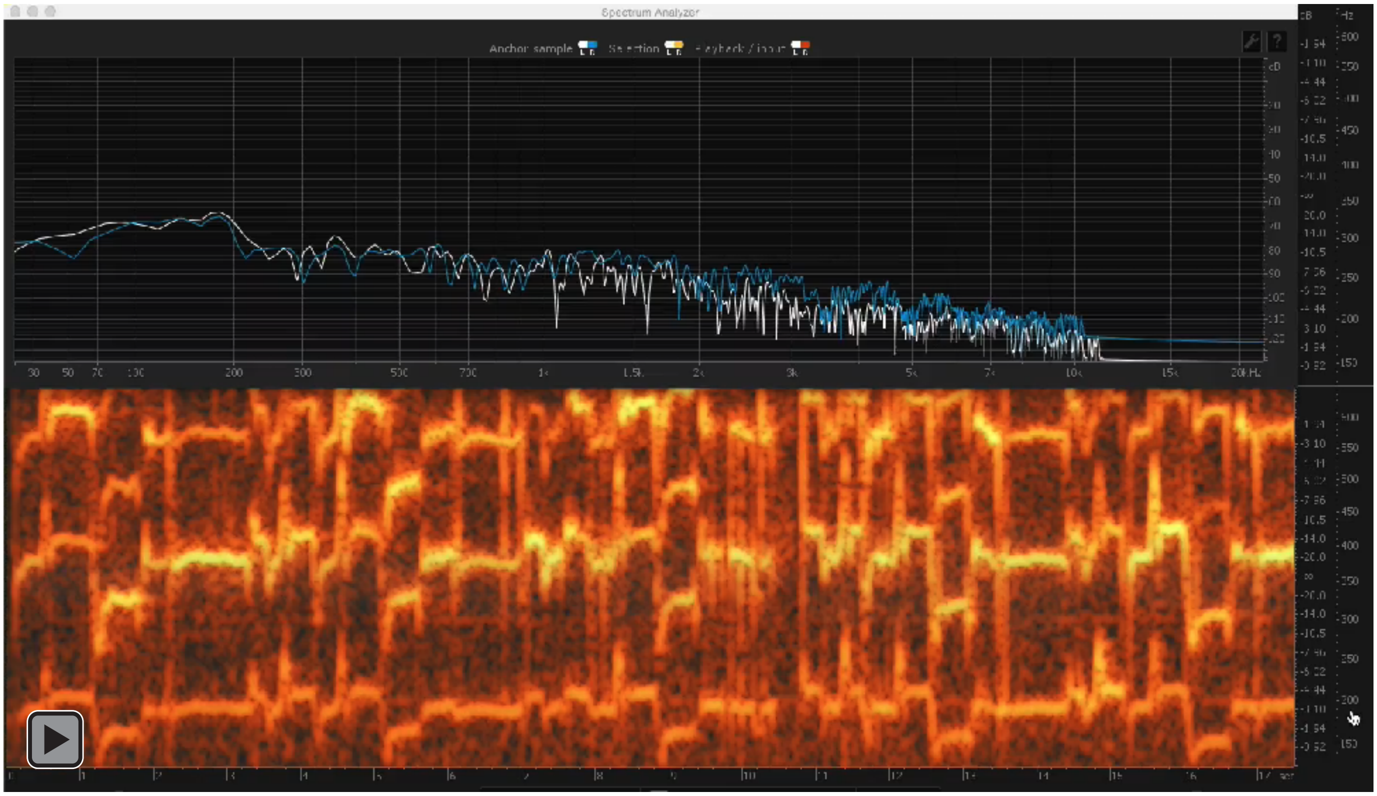

(B) "Bisik Yryata" by Ustin Nokhsorov (1946), from the Collection by Eduard Alekseyev, courtesy of Eduard Alekseyev. Used by permission.

Since there is no music in use today that would use strictly khasmatonal organization, I had to choose a fragment of a Lithuanian lullaby in order to illustrate the stylistic traits of khasmatonal modality. The most prominent are contrast and diversity of registers, dynamics and timbre. The ambitus of this song is extremely wide: from 116 Hz (A#2) to 764 Hz (G5-40 cents), measuring the fundamentals - almost 3 octaves (3,240 cents). Dynamics ranges from -23 dB of the break-off endings at the end of a phrase to -50 dB of the descending 3rd (measuring the amplitude of the fundamental tone alone). Timbral contrast plays a smaller role, but is noticeable between the "sung", "half-spoken", whispered, and breaking-off sounds. Rhythmic patterns are rather free, with considerable flexibility in relation to metric grid.

The Yakut lullaby presents a different style of organization - evident from comparing both samples: they both are 15 seconds long and encompass a 2 octave range. The ambitus is much more narrow: from the lowest infrafix degree at 158, 2 Hz (D#3-31) to the kylysakh embellishment at 277 Hz (C#4) - making 1,031 cents (about an interval of a 7th). The kylysakh is a relic of the khasmatonal style. Without it, the highest tone would be 422 Hz (G#4+28), comprising the ambitus of 559 cents (a mere tritone). The loudest fundamental is -24.8 dB, and the softest is -40 dB. Timbral contrast is not pronounced: most activity if concentrated in the first formant, which remains prevalent throughout the song. Rhythm of the formula is quite diverse, but it is repeated without much variation, and retains in strict meter.

All in all, both lullabies appear to each take a different approach toward lulling. Lithuanian lullaby entertains the child: it throws in many changes that often involve marginal opposites (high/low, loud/soft, bright/dull) to make the child feel happy and satisfied, thereby eventually relaxing him. Yakut lullaby entrains the child: it restricts regular repetition of the pattern that is moderated in frequency, amplitude, and variability in time. The music becomes completely predictable, and therefore promotes reduction in attention, causing relaxation. The same goal is achieved by different strategies and music styles - one based on contrast, and the other, on monotony. The contrasting method does not produce music with marked gravity of any of the tones. The monotonous method, on the other hand, stresses the central tone as compared to the upper and lower degrees.
