# Supplementary material for: Evolution of tonal organization in music mirrors symbolic representation of perceptual reality. Part-1: Prehistoric
Source: Front Psychol. 2015 Oct 16;6:1405. doi: 10.3389/fpsyg.2015.01405 (PMC4607869; doi:10.3389/fpsyg.2015.01405)
Supplement: Supplementary file 4 [file Presentation4.PDF]

Demonstration 4: Melodic intervals compliment harmonic intervals.

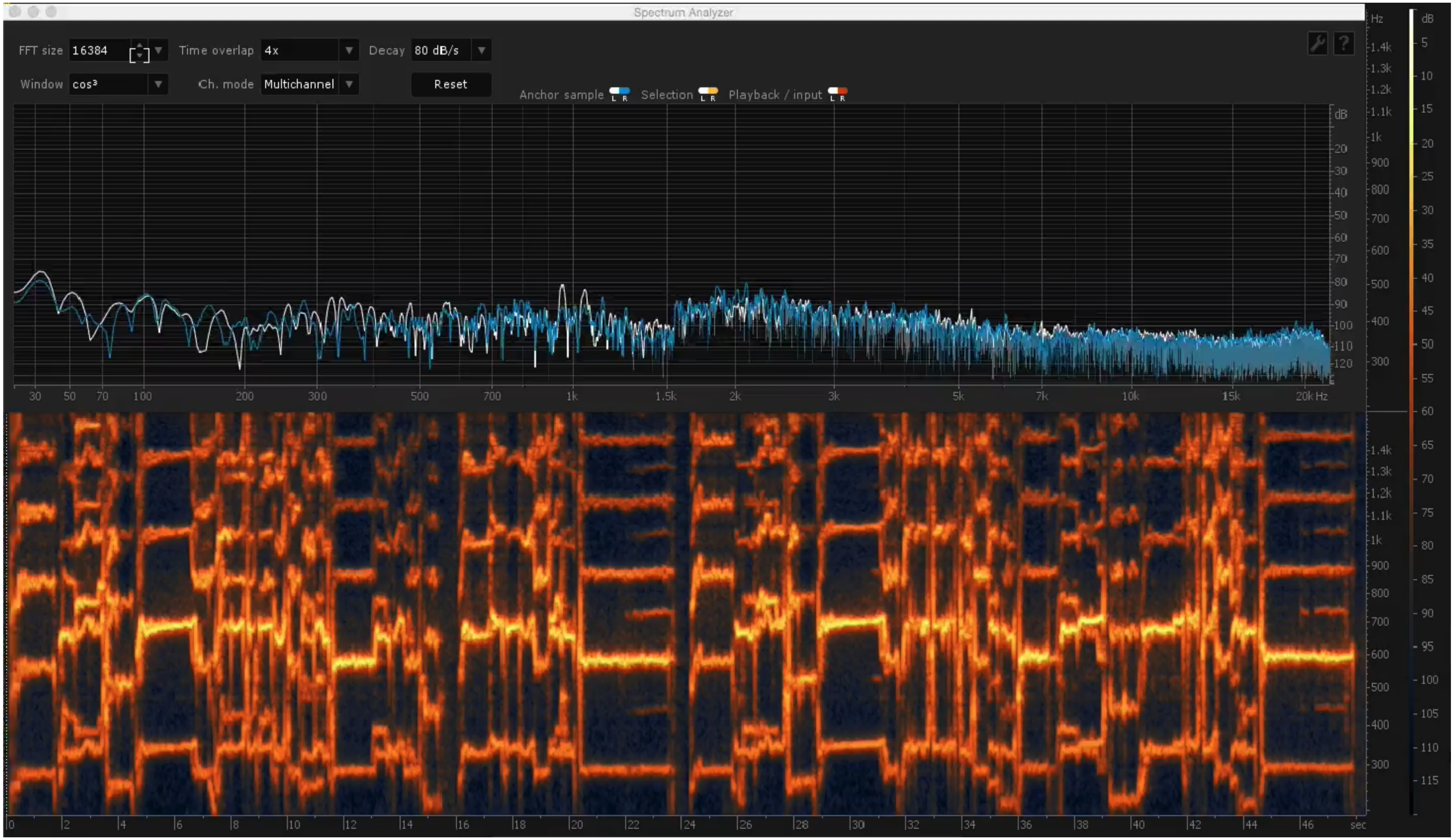

"Shelkovaia v pole travushka" by Elena Gulina and Anastasia Nikulushkina from the recording entitled Old Believers: Songs of the Nekrasov Cossacks, SFW40462, courtesy of Smithsonian Folkways Recordings. (p) (c) 1995. Used by permission.

This example presents the 1990 recording of a 2-part *besednaya* song (from "beseda" – "conversation" - a lyrical entertaining song for gatherings of youth) from the Old Believers' Cossack community that moved from Turkey to Russia in 1961. Such communities were formed by the defectors from Russia due to religious prosecutions initiated in 1652, and have remained culturally isolated for religious reasons. Their music testifies to the style of Russian village culture from a few centuries ago, featuring modest polyphony, and following Old Orthodox notation and mode system.

This particular song illustrates the tonal organization representative of the transition from mesotonal to multitonal mode. Despite featuring two parts, the ambitus is rather narrow: a mere octave. The ambitus includes, from the lowest to the highest tones, G#3 - B3 (B#) - C#4 - D#4 - E4 - F#4 (F) - G#4 (G). The E-F-E and G#-G-G# inflections are part of the mode. They relate to a specific intonation that marks the beginning of every verse throughout the song. Therefore, they should be regarded as variants of the IV degree that exists in 3 different kinds: lowered, normal, and raised - depending on its function in the intonations, where it can serve as an auxiliary tone for the singer of the lower part (flattened in III-IV-III), upper part (sharpened in V-IV-V), or a leap (neutral in I-IV) for the upper part singer.

The horizontal harmony contains the following intervals: unison, minor 2nd, major 2nd, minor 3rd, major 3rd, 4th, 5th. Interestingly, the range of vertical intervals used in a song exactly matches the melodic intervals. Noteworthy is the absence of octave in both, horizontal and vertical intervals. The vertical minor 2nd is created by the clash of F# of the lower part against G of the higher part in some of the verses, otherwise the vertical major 2nd F-G is more pronounced. Congruence of harmonic and melodic IS leads one to believe that the singers of this song project the vertical relation of their parts in terms of the melodic relations between the tones of the melody.

By no means, this song is exceptional in its intervallic design. During my participation in the ethnomusicological field-study of the music of Old-Believers' Cossack settlements nearby Izmail, 1985, headed by Nina Savelyeva, I had an opportunity to hear many similar songs from a number of villages.
