# Supplementary material for: Evolution of tonal organization in music mirrors symbolic representation of perceptual reality. Part-1: Prehistoric
Source: Front Psychol. 2015 Oct 16;6:1405. doi: 10.3389/fpsyg.2015.01405 (PMC4607869; doi:10.3389/fpsyg.2015.01405)
Supplement: Supplementary file 5 [file DataSheet1.ZIP › Appendix II.DOCX]

## Appendix: Tonal organization in tuning of Paleolithic and Neolithic pipes

Organological evidence provides the important argument for early tonal theories. Beliayev stressed that musical instruments with finger-holes and frets preserve historical stages in the development of melody by fixing those pitch values that prevail in important intonations. In this respect, such instruments are equivalent of musical notation (Beliayev 1990, 223) – they perpetuate modes that are most popular in the music-user community. It is possible to “read” this notation, following a set of principles proposed in this appendix.

The oldest musical pitched instrument so far discovered (albeit not recognized by all archeologists) seems to be a bone pipe^[[1]](#footnote-1)^ fragment from Haua Fteah, with a single hole, dated 90-110,000 years ago (Blench 2013). The next in line is the 47,000 years old bone pipe^[[2]](#footnote-2)^ from Divje Babe in Slovenia. After years of debate since its find in 1995, it was pronounced indisputably man-made by the most recent tomography (Tuniz et al. 2012). Since 1995 over 120 bone and ivory pipes have been recovered throughout Europe, dated 36,000 to 30,000 years ago, some, quite concentrated (3 pipes per site), suggesting commonality of pipe music back then (Conard, Malina & Münzel 2009).

This fact should be interpreted as testimony that European music had gone into emmelic stage by that time. Kvitka (1971, 1:221) considers flutes with holes decisive in tonal organization, because they fix tuning in a more permanent way than other portable multi-pitch instruments. Practicing flute music necessarily restricts tuning and leads to acceptance of a limited number of modes with definite pitch for at least some degrees within a local music culture.

Paleolithic pipes contain 2 to 5 holes – indicating oligotonal organization - and allow to perform anhemitonic as well as hemitonic music – judging from performance on their replicas (Atema 2004). However, their exact tuning is hard to define, since it is affected by the embouchure and closing/opening of the end of the pipe – which remain unknown for the oldest uncovered instruments. Kunej & Turk (2000) took the simplest case of a 2-hole pipe, based on the Divje Babe pipe, and listed all the pitches that could have been produced on its fresh bone replica. This gave the ambitus of hexatonic C5-A5 as a nucleus, a G4-A4 infrafix (closed end), and Bb5-C6 suprafix (closed with overblowing). Each tone could float in tuning within the range of a minor 3^rd^ or major 2^nd^ - making this pipe very suitable for playing in the ekmelic style.^[[3]](#footnote-3)^

Of course, it is impossible to tell which combinations of tones out of the available 2-octave compass the cavemen musicians preferred to use in their music making. Nor is it possible to tell to which exact scale the earliest bone pipes were tuned, because of missing mouthpieces (Atema 2014). However, some information on tonal organization of these prehistoric instruments can be extracted following Beliayev’s methodology (Belaiev 1963). Based on his encyclopedic knowledge of folk organology, he outlined the principal stages in evolution of modal organization in tuning practices of the instrumental music. He called it "the system of metric temperament" (Beliayev 1990), where "metric" referred to intuitive decision of early instrument makers to measure and memorize the distances between holes and frets by eye. Sachs also made a note of this tendency, speaking about contribution of visual and tactile senses to decision making in construction of the early musical instruments (Sachs 1962, 99). Kvitka (Kvitka 1971, 1:221) even considers visual factor to be decisive in development of tonal preferences of the Stone Man, who must have started his experiments by drilling two holes in the positions most comfortable for placement of his fingers, and then either tried to reproduce the distance between these holes symmetrically, or followed a pattern of alternation of two distances. Liking certain intervals probably came second, after experimenting with the holes.

However, this experimenting must have been constrained by already existing models of vocal music. Either through pipe emulation of vocal melodies or through pipe accompaniment to singing, the tonal organization of vocal music would have influenced the choice for the intervals between the holes. At the end, a pipe-maker would select the set of anchor tones most suitable to the vocal music in place at his community, and drill the holes in places that would produce the desired intervallic relation between such anchors. The unstable indefinite in pitch tones were likely to be left for production by tweaking the embouchure. Practicing such pipe music would make a feedback influence on vocal music by securing more uniform tuning for the anchor tones.

The principal formative interval on majority of Eurasian "primitive" instruments, according to Beliayev, is a perfect 4^th^ (498 cents). Its importance probably has to do with the cognitive issues (see part-2). Beliayev considers formative 4^th^ a landmark of professionalization for a folk music culture (Beliayev 1990, 248). Sachs also stresses importance of 4^th^ for native Northern American, Central Asian, Far Eastern, European, Caucasian and Maghreb regions – being more rare in Melanesia and Central Africa (Sachs considers 4^th^ to be a later development there, following the establishment of 3^rd^), and absent only in Polynesia and Micronesia (Sachs 1962, 163).

"Metric division" of the 4^th^ in two produces *psychoacoustically* *contrasting* intervals: the displacing 2^nd^ and the tracing 3^rd^. Probably, instrument makers followed the theoretic idea of dividing the 4^th^ into two near-equal parts, after which they empirically discovered that melodies based on exact division were hard to process, and then went into experimentation, trying to define the closest to equal proportion that would produce satisfactory sounding intervals. Perceptually, the smallest value that triggered “tracing” in their hearing was likely to be taken as the defining point for the trichord division, since the contrast between non-tracing 2^nd^ and tracing 3^rd^ would present a convenient way to the ear to identify each of the produced tones.

Beliayev concluded that the primordial modal nucleus was the trichord of the C-D-F type^[[4]](#footnote-4)^, cultivated in 2-hole pipes and 2-fret stringed instruments. Such nucleus comfortably extends into the pentatonic mode on wind instruments by forming two disjunct trichords C-D-F/G-A-C, and on string instruments - conjunct trichords C-D-F/F-G-Bb. Uneven division of the 4^th^ directs music towards decentralized gravitational model (see the chapter on pentatony).

Definition of the metric tetrachord had to follow a different principle: the chain of 2^nds^ must have been seen as “linear”: directed towards the lower or upper tone in the tetrachord, where minor 2^nd^ would compliment “resolution” in the chain of major 2^nd^s. It should be reminded that instrumental construction and fine-tuning of tones follows the intuitively defined vocal tradition that must be already in place. Melodic directionality characterizes vocal hemitonic music just as much as melodic cyclicity characterizes anhemitonic pentatonic music (Zemtsovsky 1998). Fine-tuning of tetrachords was the issue of finding the most satisfactory succession of pitches that would produce the best cadence to stress the marginal tones in a tetrachord.

The ternary and binary divisions of a 4^th^ lead to opposite methods of tonal organization, each with its own cognitive concomitant.

- Ternary division generates near-equal steps, using of which in composition promotes diatonicity,^[[5]](#footnote-5)^ incrementality, and proportionality – that lays the ground for hierarchic organization and centralization of the mode. There is evidence that nonmusicians have reduced frequency discrimination at interval differences below 125 cents (Zarate, Ritson & Poeppel 2012), which suggests that diatonic steps are processed as similar in melodic quality, regardless of whether they are minor or major 2^nd^ in size. If that is the case, then smooth melodic motion in such music would engage incremental thinking – relating all intervals in terms of proportion to the interval of the major 2^nd^, and defining the minor 2^nd^ variety as a derivative of the major one. Major 2^nd^ is preferred to minor 2^nd^, and is more frequently distributed in folk music (Huron 2001, 25).
- Binary division generates unequal steps – uneven enough for the listener to memorize its pitches’ respective position in the PS (especially since there are fewer pitches in a binary as compared to a ternary division). Processing of smooth melodic motion in such conditions would resemble more of thinking in binary computer language – as opposed to incremental thinking in processing of ternary division.

It seems that in general it was the instrumental music that rationalized tonal organization. Instrumental music cemented the emmelic nucleus, and directed the oligotonal mode to commit either to pentatonic or to heptatonic direction. Beliayev (1990, 280) believed that the earliest tonal organization was governed by vocal genres. Construction of pitched instruments cultivated a rational way to fine-tune and redefine vocal intonation in terms of concisely defined intervals, at first perfect 4^th^, then perfect 5^th^, then their difference (5^th^ – 4^th^ = 2^nd^)^[[6]](#footnote-6)^ and their sum (5^th^ + 4^th^ = octave), which is more obvious visually on instruments than in the singing voice.

The entire perspective of general evolution of tuning then appears as:

1. Progression from indefinite pitch to pitch, defined by its membership in one of 2-3 timbrally marked registers (khasmatony);
2. More specific pitch definition within a range of 2-4 stretchable pitch zones, coordinated numerically by their registral position in relation to an anchored degree (ekmelics);
3. 1-2 definite pitches (2^nd^ to a 4^th^ apart) and 1-2 indefinite pitches (in-between or/and aside in relation to the definite one), processed as complimentary to the definite anchors (ekmelic oligotony).

At this point instrumental music becomes important and takes the lead in modal genesis away from vocal music.

1. Emergence of the “step-equivalence” in increments of “neutral” or “major” 2^nd^s (emmelic oligotony);

Next is ambitus expansion:

1. Rise of vertical harmony and emergence of equivalence of 3^rd^ (mesotony);
2. Emergence of equivalence of 4^th^ and its ternary or binary division (non-octave heptatony/pentatony).

Tuning schemes elaborated in instrumental music in turn start influencing vocal music. Cultures that do not cultivate instrumental music, such as Nenets, do not progress toward diatonic order, freezing at the oligotonal anhemitonic stage with remnants of khasmatonal and ekmelic organization (Niemi 1999). Otherwise, subset structures (tetrachords and trichords) develop their own typology and integrate into complex hierarchies with overall centripetal order:

1. Tetrachordal organization in heptatonic music leads to the discovery of even/odd functionality of degrees, which favors equivalence of 3^rd^ over equivalence of 4^th^ and marks the pentachord as a modal foundation (multitony);
2. 4^th^ is realized as an inversion of 5^th^, enabling establishment of octave equivalence at first for “tonic,” then for “dominant” and “mediant” (octave-equivalent heptatony/pentatony);
3. Big number of octave-equivalent degrees suggests discovery of equivalence of 5^th^ and application of it to the entire ambitus, equalizing the degrees through the circle of 5^ths^ and full octave equivalence of all degrees (diatony).

Beliayev's approach to 4^th^, 5^th^ and octave (Beliayev 1990, 258) can be successfully combined with the intonation theory of Yavorsky/Asafyev/Mazel (Protopopov 1930) (Tull & Asafyev 2000) (Mazel 1982), and implemented in analysis of modal organization of tuning of the Stone Age pipes on the following premises:

1. No matter whether a pipe is played in ney, quena, or fipple style, the intervals between the tones produced by each of the pipe holes remain the same. Changing the way of blowing affects all the holes equally, shifting their respective pitches up or down the same amount (Münzel, Seeberger & Hein 2002, 109).
2. Tones that are easier to produce are used more frequently and are known to form the basis for popular and folk music (Green 2002); on another hand, more stable tones are easier to process than unstable tones – even for non-musicians (Marmel & Tillmann 2009).
3. On majority of wind instruments tones that are easier to produce are lower in pitch, and therefore appear to have a richer harmonic spectrum, since more of their harmonics fall within the critical bandwidth of human hearing (Rasch & Plomp 1999). Hence, richer timbres are usually perceived as "nicer" (Pratt & Bowsher 1978).
4. Good tone quality is generally preferred to poor tone quality (Fung 1995). Pleasantness of consonant spectral content appears to be perceived pre-attentively (Bidelman & Grall 2014), testifying to the genetic roots of consonance discrimination, and suggesting that cavemen musicians shared our ability to detect pleasant tones.
5. More pleasant tones are likely to be used more frequently and sustained for longer, as compared to less pleasant tones (Costa, Fine & Bitti 2004).
6. Tones that are more frequently repeated (Aarden 2003) and assigned longer time values (Smith & Schmuckler 2004), in music not conforming to Western tonality (Cuddy 1997), are perceived as more stable (Bharucha 2002) proportional to their frequency of repetition (Krumhansl 1990, 67).
7. Once the anchor tones are internalized, it is no longer necessary for music to follow the usual statistical regularities to be perceived as gravitating toward an anchor point (Bigand et al. 2003).
8. Tones that are lower in pitch appear "bigger" in size due to cross-modal connection that is normative for humans. This connection appears to be an adaptive perceptual process (Bien et al. 2012) that might have a genetic origin (Dolscheid et al. 2014).
9. Therefore, lower tones are more prone to produce the impression of greater gravity, and attract other tones. The descending melodic motion causes greater displacement in memory for the last tone towards the direction of the motion than that in the ascending melodic motion – corresponding to the implied gravitational attraction in descending visual motion (Hubbard & Courtney 2010).

So, the fundamental tone of a pipe, and tones produced by opening each of the holes – especially the lowest in pitch – determine the modal application of the pipe in question: these tones are likely to form the anchor tones in the melodic formula produced on such pipe. Other tones could have taken prominence only after a player would have become comfortable at the production of main pitches to have then started experimenting with half-covering the holes, blowing at different angles, etc., deriving new pitches and memorizing ways of their production. However, such experimentation must have been severely limited by the collective nature of early music.^[[7]](#footnote-7)^ The low unstable tones (i.e. plagal inclinations) of vocal modes were likely to be omitted in instrumental adaptation – at least in the earliest pipes. Performance of tonally principal pitches must have been maximally simplified so that any member in a social group could potentially produce tones important for collective musicking.

Comparative distance between intervals of the same class provides yet another glimpse into tonal organization within the early mode. If there are two kinds of 2^nd^ found in an IS, then the larger interval is likely to separate the tones, whereas the smaller one – to join them into a “melodic intonation.” A subordinate tone tends to be close to its “master” tone (as VII degree to I) because of commonality of intonations, where that subordinate tone would resolve into its master – what David Huron calls “tendency tones” (Huron 2006, 161). The expressive tuning templates characteristic for a given mode are going to sharpen or flatten that subordinate tone even more to facilitate “resolution.” This happens as a result of the same perceptual mechanism that is engaged in visual *Fröhlich effect –* the impression of a displacement in the direction of target motion (Hubbard & Ruppel 2013).

However, two neighboring tones that have coordinate relation (as VI and VII degrees in natural major and minor) do not comprise “tendency tones” (160), and are not brought closer to each other by expressive tuning. That is why differently sized 2^nds^ in an IS is a likely sign of modal intonations peculiar to one degree in the PS – but not to others.^[[8]](#footnote-8)^

Perpetuated in the placement of holes on the prehistoric pipes, contrast in size of a 2^nd^ should be interpreted as something similar to difference between modern “clarinet in Bb” or “A”: design of an instrument with the purpose to facilitate performance of certain combinations of tones^[[9]](#footnote-9)^. In a music culture that lacks professional music performance, the chances that one of the lowest producible tones constitutes an anchor in majority of melodies of that culture is rather high. A tone that comprises the smallest 2^nd^ with the neighboring hole, and the 4^th^ with the fundamental or some other hole is likely to be the “tonic” of that pipe. Presence of well-tuned octave and 5^th^ in the tuning set provides an extra confirmation of “tonicity” – in addition to the contrast in 2^nds^.

The oldest multi-hole Divje Babe pipe comes from the Neanderthal site and appears to be designed as a sum of steps without concise attention to their exact size. The demo of the reconstruction by Jelle Atema (2014) contains C#6 (+42 cents), D6 (+23), E6 (-1) and F#6 (-45) – comprising a squashed tetrachord 81:176:154 (very flat 4^th^ of 411 cents). According to Atema’s reconstruction experiments, these basic tones retain their intervallic proportions, whether the pipe is played with a fipple or quena interface. This version misses the mark of perfect 4^th^, indicating 2^nd^ as the primary formative interval in the PS design. Dissimilarity of the size of the 2^nds^ should be interpreted as unawareness of the idea of metric temperament. D (or C#) is the most likely candidate for the tonal center.

The more recent 2015 reconstruction by Jelle Atema added a thumb hole on the back of the pipe (to account for the controversial opening on the original pipe that might constitute damage rather than intended hole). The extra hole affected the tuning in the following way: D#6 (-44), E6 (-6), F# (-29), G# (+13) – producing the 138:165:242 tetrachord with a 545 cent augmented 4^th^. The rest of the tones produced with simple fingering are: A6 (+41) and B6 (+28). The 4^th^ E-A is augmented, too, at 535 cents. So is the F#-B 4^th^ at 557 cents. The 2^nds^ on the thumb version are of different size, but three tritones are surprisingly similar, suggesting that the tritone was of formative importance for the music played on that pipe. Alekseyev reports that tritone mode is quite common in the Siberian folk music, where it often occurs as the result of integration of major 2^nds^, which often occurs in genres associated with display of vigor (Alekseyev 1976, 199).

Whichever version is more authentic, the tonal organization of this pipe tuning indicates the simplest modal genesis of adding 2^nds^ together in an attempt to find the optimal consonance values for the PS.

The 15,000 years younger (Conard, Malina & Münzel 2009) 5-hole Hohle Fels-1 pipe presents an amazing advance in tonal organization by featuring octave equivalence between its fundamental tone and its 1^st^ hole, as well as a transposition of a trichord a 5^th^ apart – indicative of octave-equivalent pentatony. The replica of Hohle Fels pipe, commissioned by Nicholas Conard (McGroarty 2009) from Wulf Hein^[[10]](#footnote-10)^ from the University of Tübingen, according to the sample of it published by the New York Times (Wakin 2009) contains the following tones: Eb5 (+20), F5 (-15), G5 (+20), Bb5 (+22), C6 (+62) and ) Eb6 (+41). This PS features a pronounced F-G-Bb "metric trichord," comprising an augmented 4^th^ - 235:302 (537), and a quite similar trichord Bb-C-Eb: 240:279 (519) placed a perfect fifth above the fundamental tone (such clear patterning of trichords looks very advanced as compared to other Paleolithic instruments). The third 4^th^ contained in the nucleus, between G5 and C6, is also augmented at 542 cents. The uniformity of tritone seems to follow the Divje Babe pipe version with a thumb-hole. However, the Hohle Fels pipe features a well-defined octave between the pipe and the hole highest in pitch, strongly suggesting a pentatonic organization with octave and 5^th^ equivalence. If that is the case, the pipe testifies to a major advance in tonal organization, as compared to the older, as well as the contemporary pipes.

About 2,000 years younger, the 3-hole Geißenklösterle pipe-1 demonstrates a regression in relation to the Hohle Fels-1, and a progress compared to the Divje Babe pipe. In contrast to the latter, this pipe produces clear pitched and loud tones (Münzel, Seeberger & Hein 2002, 108). The sample of the replica of this pipe by Seeberger^[[11]](#footnote-11)^ contains the following pitches: Cb5 (-11), Db6 (+46), F6 (-30), Bb6 (-2), Cb6 (+7), Db7 (+11), F7 (-41). The 3 holes are tuned to Db-F-Bb on the fundamental Cb (256:324:528). Evident scaling of the intervals between holes – 2^nd^, 3^rd^, and 4^th^ – suggests that the maker was exploring methods of distancing the holes and was concerned about variety of intervals to be available for production of melody. The pipe could have been designed for ascending inclination of the ekmelic music, with characteristic expansion of the ambitus towards the top tone (Alekseyev 1976, 50).

Overall, Geißenklösterle pipes seem to indicate the maker’s awareness of the contrast between melodic stepping and leaping. D'Errico (D'Errico et al. 2003) also noted the idiosyncrasy in separating the second and third holes by longer distances in the earliest bone pipes, considering it a trait of the Aurignacean culture, common across Europe, and maintaining its influence throughout the Gravettian period. The holes separated by longer distance in this design are marked by linear divisions engraved on the pipes between the second and the third holes, suggesting the instructional purpose of these engraved lines. It is quite possible that they served to provide visual aid for the player to separate intonations produced on the first two holes as opposed to the neighboring holes. Such separation would be very logical for a musician who had just come to grasp with the idea of contrast between the intervals of 2^nd^ and 3^rd^. Boris Frolov discusses marks on the musical instruments in relation to the paintings on walls of caves where the instruments were found, as well as other retrieved artifacts. He concludes that the marks on the pipes must have signified a particular sound – thus, crosses on the Avdeyevo pipes were found also on the figures of the prey animals and male belongings, suggesting connotation with hunting (Frolov 1992, 165).

The next generation 4-hole 83888(a) Isturitz pipe contains a Beliayev’s trichord within a “lean” 4^th^ along with the chain of 2^nds^ and 3^rds^, testifying to the importance of their contrast in the music made on that pipe. The reconstruction of this pipe, accomplished at Zaragoza University (Mazo, Gracia & Benito 2014), produced the following pitches when the ney embouchure was employed: Ab5 (0), Bb5 (-15), C6 (-9), Eb6 (-45), and G6 (-49).^[[12]](#footnote-12)^ The 3 holes lowest in pitch comprise a clear "metric trichord" Bb5-C6-Eb6 (194:264, with a flat 4^th^ of 458 cents). The tone above the trichord is a fair 3^rd^ (302 cents), which does not form perfect relation of octave or 5^th^. Neither the fundamental tone forms a good 5^th^ in relation to Eb. Bb is marked by the combination of the smallest 2^nd^ (Ab-Bb 185 cents) and 4^th^. The idea behind this tuning seems to be the ekmelic ascending expansion: 194, 264, 302 cents – very much like in the Geißenklösterle pipe-1.

Solutrean Grubgraben pipe replica introduces the first clean 5th in combination with the basic trichord, producing a well-sounding triad – indicative of the mesotonal or possibly pentatonic modes.^[[13]](#footnote-13)^ The fundamental tone of the pipe and its 3 holes are tuned to: F6 (+30), G6 (+46), Bb6 (+30), and D7 (+36). Not only that this tuning contains a perfect 4th (500 cents) serving as the base for the trichord at the bottom of the ambitus, but the perfect 5th between G and D (690) suggests octave equivalence (500 + 690 = 1190).

Of course, this comparative overview of bone pipes should be taken as a very general outline, since I am comparing the tonal properties of replica instruments,^[[14]](#footnote-14)^ and some of the original instruments do not provide a decided answer as to which end an instrument was meant to be blown into.^[[15]](#footnote-15)^ Ideally, a replica should be built from the same material as the original – a bone, and to replicate all the details of the design, such as distances between the holes, their position in relation to the tube axis, the angle of the cut, etc.. An ideal setting for an experimental comparison of tonal qualities of all the survived early bone pipes would be to build all the replicas from the same material, with the same embouchure, and test them applying the same blowing angle, pressure level, and manner of fingering. Only then the melodic intervals between the holes would provide reliable testimony to the progress in tonal organization between different prehistoric periods. Until such uniformity is reached, each faithful pipe replica can only testify for itself.

The oldest original pipe in playable condition dates to 7,000 BC, excavated in Jiahu site, Wuyang, China (Zhang, Xiao & Lee 2004).^[[16]](#footnote-16)^ The oldest of Jiahu pipes, pipe M341:1, is comparable in its tonal design to the Grubgraben pipe. It has G5 as its fundamental tone which is separated by 215 cents from A5, the lowest pitched hole. Its second lowest hole C6 is separated from the first by 325 cents – producing somewhat of an augmented 4^th^ (G5-C6) of 540 cents. Another 4^th^ (A5-D6) is almost perfect at 507 cents. These tones comprise a trichord-based nucleus (G-A-C-D) with additional two leaping upper tones (G6-C7). The D6-G6 4^th^ is perfect (497), but the G6-D7 one is flatter (487). The 5^th^ G5-D6 is 722 cents, yet another 5^th^ C6-G6 is considerably flatter at 675 cents. The PS here is neither 5^th^ nor 4^th^ equivalent, but with quite well-tuned octave, and makes an impression of being representative of the transition between oligotonal and pentatonic organization, with stability axis along “tonic” G and “dominant” D.^[[17]](#footnote-17)^

The second oldest pipe M341:2 is similar in design, but has an extra hole. It features a nearly perfect octave equivalence, and its pipe’s fundamental tone is “gapped” a 3^rd^ lower than the nearly perfect Beliayev's trichord: C6-D6-F6. Extra holes add G6, A6, and a leaping D7 at the very top of the ambitus. The tuning of the C-D-F trichord is 197:304 - the 4^th^ is slightly bloated at 501 cents, but by no coincidence: the 4^th^ between the fundamental A5 and D6 is 501 cents as well. And the 4^th^ between C6 and F6 is 505 cents – so as the highest 4^th^ A6-D7. This pipe represents a major improvement in tonal organization as compared to the M341:1 pipe: equivalent 4^ths^ and octaves testify to the development of octave-equivalent pentatonic order with very likely tonic on D. Coexistence of both pipes at about the same time points toward the speed in advance towards pentatonic organization – suggesting 7,000 BC as the date of rise of pentatonic mode in Far East region.

Two pipes constructed 400 years later present yet another development from trichordal to tetrachordal design, testifying to the discovery and exploration of hemitonic music. Each of the two pipes feature different tetrachords, “Phrygian” and “Aeolian,” which correspond respectively to vocal (Alekseyev 1976, 110) and instrumental practices of music-making (Kushnaryov 1958). Both pipes are tuned to fundamental F5, suggesting that they might have been intended to play together. M282:20, the older of the two, has 6 holes tuned to consecutive steps (except the highest tone): A5-B5-C6-D6-E6-F6-A6, with A6 equivalent to A5. This new design appears to experiment with the diatonic principle of distribution of steps, resulting in obvious hexatonic organization. This pipe’s nucleus is based on the "vocal tetrachord" A-B-C-D (178:152:200, augmenting the 4^th^ to 530 cents – probably as a consequence of novelty of the idea of breaking of 4^th^ into 4 parts). Another 4^th^ between B and E is even more bloated at 552 cents. The C-F 4^th^ is normalized at 500 cents – so as the highest E-A 4^th^. It looks like this design favored octave equivalence over equivalence of the 4^th^, which somewhat became disturbed by the division in the hemitonic tetrachord.

M282:21 also features 7 holes, but differently tuned. The nucleus features a competing to M282:20 tetrachord - "instrumental" metric A5-Bb5-C6-D6 (104:277:156, with the bloated 537 cents wide 4^th^). The coexistence of two different tetrachords in two contemporary pipes should be interpreted as the competing influence of vocal and instrumental music making in Jiahu culture. The other 4^th^ between C6 and F6 is 474 cents, and the lowest 4^th^ between the fundamental F5 and Bb6 is 468 cents – all on diminished side. The upper 3 holes F-Gb-A puzzle with extremely narrow space of 35 cents between F6 and Gb6 (or F#). Such distance between neighboring degrees can be found occasionally in exaggeratedly expressive intonation between the leading tone and tonic in tonal music. It barely falls within the range of detectable tuning deviations reported in existing folk music systems (Léothaud et al. 1997) that correspond to mesotonal and pentatonic stages of tonal development. However, possibility of a 35 cent intonation in Neolithic culture remains an open question.^[[18]](#footnote-18)^

The fifth pipe, M253:4, made 400 years after M282:21 (6,200 BC), is even more experimental. It adds an eighth hole, squeezes the ambitus, and injects into the PS "chromatic" degrees. Like the previous 2 pipes, this one is also tuned to F5. The nucleus here is comprised of the chromatic hexachord, A5-Bb5-B5-C6-C#6-D6 (115; 115; 18; 176; 46), that is apparently derived from an attempt to divide a 4^th^ into 6 parts - where Bb/B and C#/D are microtonally squeezed together (like in a Greek enharmonic genus). The resulting 4^th^ (A-D) is still narrow at 470 cents. The rest of the holes are F6 and G6 placed 377 cents above D6, producing an augmented 4^th^ (D-G) equal to 577 cents. This pipe seems to present an attempt to explore chromatic subdivision. It is hard to tell whether chromatic melodies were performed on this instrument, or whether a single instrument was designed to serve the purpose of playing in two different PSs (i.e. F-A-Bb-C-D-F or A-B-C#-E-G). Yet another possibility is that this instrument was intended to match in pitch some other instruments (important for the ensemble performance) that differed in tuning (like M282:20 and M282:21 – it’s hardly a coincidence that M282:21 and M253:4 have their second lowest hole in pitch tuned to exactly the same value in cents). In that case, we have an indication of polyphonic music cultivated at Jiahu. At any rate, this pipe is proof that prehistoric Chinese were already well aware of the taste of hemitonism, and their later historic choice for pentatonism was completely "educated."

The triadic principle of tonal regulation, inherent for hemitonic music, must have been known to Jiahu musicians, since those younger pipes that shared tetrachordal design were co-tuned: pipes M282:20 and M282:21 shared a D that was tuned just 2 cents apart (suggesting leaning on the axis of A-D), whereas M282:21 and M253:4 shared Bb that perfectly matched (suggesting importance of the F-Bb tonal axis). Co-tuning testifies towards ensemble performance practice, and hence, might be taken as a sign of the derivation of vertical harmony from horizontal harmony. Further support for polyphonic practices comes from recovery of the Zhongshanzhai tuner, at the nearby Jiahu, in Ruzhou (Zhang, Xiao & Lee 2004). Dated 6,000 BC, it contains 10 holes, aligned in two rows tuned to approximately 100 cents apart – presenting the world earliest attempt of temperament (apparently by following the principle of joining two "metric pentachords," each dividing the 4^th^ into 5 equal parts). This instrument was not intended for music-making: the distances between holes are so tight that it is difficult to place a finger to cover a single hole. The purpose of this artifact must have been solely for tuning, which then goes to prove that Jiahu music-users were aware of vertical intervals. As Mazel's triadic theory postulates (Mazel 1952, 61), the hemitonic music promotes usage of vertical intervals and chords. Chinese musicians must have experimented with it during the Stone Age and abandoned it during the Iron Age in favor of pentatony.

The 5-hole Veyveau pipe from France (Fages & Mourer-Chauvire 1983), 4,000 years younger than the Jiahu pipes, presents an accomplished multitonal mode. The degrees in this pipe's PS are functionally united by the subordinate as well as the coordinate relations. The set, however, is subverted under the influence of triad induction (Mazel 1952, 61) and brings out strong pairing tendencies. It is highly probable that music produced on the Veyveay pipe featured triadic organization between stable E6-G#6-B6 and unstable D#6-F#6-A#6, with the lowest B5 and C#5 serving as the infrafix – engaged into modal alternation of 2 tonal centers (modal mutability) between E6 and B5, or between E6 and C#6.

The pipe is tuned to A# (Atema 2014) and, together with 5 holes, it produces the following tuning set: A#5 (+43), B5 (+16), C#6 (+5), D#6 (+30), E6 (+2), and F#6 (-10). Compared to its European predecessors, the Veyreau pipe noticeably shrinks the intervals between the holes – a proof of the centripetal gravitational force imposed by centralization of the heptatonic mode. The nucleus is constructed of the expanded "vocal" tetrachord B-C#-D#-E (189:225:72, with nearly a perfect 4^th^ of 486 cents) which testifies to the importance of vocals in music produced with the help of this pipe. Mazel's triadic principle manifests itself in pairing B5 with C#6 (by tightening the 2^nd^ between them, as compared to the 2^nd^ between C#6 and D#6), and even stronger tightening between D#6 and E6. F#6 forms a pair relation with G#6 (-25). The upper A#6 (-31) is pulled lower probably in order to expand the F#-G# pair into F#-G#-A# trichord progression, suitable for music where the melody needed some variety. However, A#6 clearly favors the ascending motion (leading function) by featuring a smaller interval in the PS: toward the upper B6 (-55) – the interval between this A# and B makes 76 cents versus 193 cents between F# and G#. The higher tones, C#7 (-55) and D#7 (-35), are not octave equivalent, and are clearly pushed down in their tuning by the strong centripetal force.

All in all, judging by the IS, this pipe is designed with E6 as a tonal center. D#6 serves as a leading tone with its smallest interval value (72) to E. The upper tetrachord F#-G#-A#-B (185:194:76 with a more narrow 4^th^, equal to 455 cents) reproduces the lower tetrachord B-C#-D#-E in its proportions and pairing. Here we see the same principle of superimposition of tetrachords at work, that later became the mainstream for tonal organization of Western classical music. B5 must have served as the “dominant” tone to “tonic” E5 (C#6 is separated from the leading tone D#7 by the largest 2^nd^ of 225 interval) – a modal infrafix, very likely to induce modal alternation between B and E. F#6 must have served as the suprafix to E6 (188 cents, one of the larger 2^nds^), with its own autonomous tetrachord. There are signs of, both, subordination and coordination, evident in this PS.

Yet another interesting point is that the Veyreau pipe has a little hole for hanging it on one's neck (Atema 2014). This could testify to professionalization in music-making: a person in need of wearing a musical instrument is likely to use it often. Then, the Veyreau music must have corresponded to the multitonal stage that is characterized by growing complexity in performance and increased role of competence in musical syntax, as well as greater importance of the performance technique.

REFERENCES:

Aarden, B.J. 2003. “Dynamic Melodic Expectancy.” Ohio State University.

Alekseyev, Eduard. 1976. *Problems in Genesis of Mode [Проблемы Формирования Лада]*. Muzyka [Музыка].

Atema, Jelle. 2004. “Old Bone Flutes.” *Pan, Journal of the British Flute Society* 23 (4): 18–23.

———. 2014. “Musical Origins and the Stone Age Evolution of Flutes.” *Acoustics Today* 10 (3): 25–34.

Belaiev, Victor. 1963. “The Formation of Folk Modal Systems.” *Journal of the International Folk Music Council* 15: 4–9. doi:10.2307/836227.

Beliayev, Viktor. 1990. “Modal Systems in the Traditional Music of the USSR [Ладовые Системы В Музыке Народов СССР].” In *Viktor Mikhailovich Beliayev [Виктор Михайлович Беляев]*, edited by Irina Travkina, 223–377. Moscow: Sovetskii Kompozitor [Советский композитор].

Bharucha, Jamshed J. 2002. “Neural Nets, Temporal Composites, and Tonality.” In *Foundations of Cognitive Psychology: Core Readings*, edited by Daniel Levitin, 455–80. Cambridge MA: Bradford Books MIT Press.

Bidelman, Gavin M., and Jeremy Grall. 2014. “Functional Organization for Musical Consonance and Tonal Pitch Hierarchy in Human Auditory Cortex.” *NeuroImage* 101 (November). Elsevier Inc.: 204–14. doi:10.1016/j.neuroimage.2014.07.005.

Bien, Nina, Sanne ten Oever, Rainer Goebel, and Alexander T. Sack. 2012. “The Sound of Size: Crossmodal Binding in Pitch-Size Synesthesia: A Combined TMS, EEG and Psychophysics Study.” *NeuroImage* 59 (1). Elsevier Inc.: 663–72. doi:10.1016/j.neuroimage.2011.06.095.

Bigand, Emmanuel, Bénédicte Poulin-Charronnat, Barbara Tillmann, Francois Madurell, and Daniel A. D’Adamo. 2003. “Sensory versus Cognitive Components in Harmonic Priming.” *Journal of Experimental Psychology. Human Perception and Performance* 29 (1): 159–71.

Blench, Roger. 2013. “Methods and Results in the Reconstruction of Music History in Africa and a Case Study of Instrumental Polyphony.” *Azania: Archaeological Research in Africa* 48 (1): 31–64. doi:10.1080/0067270X.2013.771016.

Conard, Nicholas J, Maria Malina, and Susanne Münzel. 2009. “New Flutes Document the Earliest Musical Tradition in Southwestern Germany.” *Nature* 460 (7256): 737–40. doi:10.1038/nature08169.

Costa, M., P. Fine, and Ricci Bitti. 2004. “Interval Distributions, Mode, and Tonal Strength of Melodies as Predictors of Perceived Emotion.” *Music Perception* 22 (1): 1–14.

Cuddy, Lola L. 1997. “Tonal Relations.” In *Perception and Cognition of Music*, edited by Irène Deliège and John A. Sloboda, 330–52. Hove, UK: Psychology Press.

D’Errico, Francesco, Christopher Henshilwood, Graeme Lawson, Marian Vanhaeren, Anne-Marie Tillier, Marie Soressi, Frédérique Bresson, et al. 2003. “Archaeological Evidence for the Emergence of Language, Symbolism, and Music — An Alternative Multidisciplinary Perspective.” *Journal of World Prehistory* 17 (1): 1–70.

D’Errico, Francesco, Paola Villa, Ana C. Pinto Llona, and Rosa Ruiz Idarraga. 1998. “A Middle Palaeolithic Origin of Music? Using Cave-Bear Bone Accumlations to Assess the Divje...” *Antiquity* 72 (275): 65.

Dolscheid, S., S. Hunnius, D. Casasanto, and A. Majid. 2014. “Prelinguistic Infants Are Sensitive to Space-Pitch Associations Found Across Cultures.” *Psychological Science* 25 (6): 1256–61. doi:10.1177/0956797614528521.

Fages, G., and C. Mourer-Chauvire. 1983. “La flûte en os d’oiseau de la grotte sépulcrale de Veyreau (Aveyron) et inventaire des flûtes préhistoriques d'Europe in La faune et l'homme préhistorique.” *Mémoires de la Société Préhistorique Française Paris* 16: 95–103.

Frolov, Boris. 1992. *Primitive Graphics of Europe [Первобытная Графика Европы]*. Moscow: Nauka.

Fung, C. Victor. 1995. “Music Preference as a Function of Musical Characteristics.” *The Quarterly Journal of Music Teaching and Learning* 6 (3): 30–45.

Green, Lucy. 2002. *How Popular Musicians Learn: A Way Ahead for Music Education*. New editio. Aldershot, Hants ; Burlington, VT: Ashgate Pub Ltd.

Hubbard, Timothy L., and Jon R. Courtney. 2010. “Cross-Modal Influences on Representational Momentum and Representational Gravity.” *Perception* 39 (6): 851–62. doi:10.1068/p6538.

Hubbard, Timothy L., and Susan E Ruppel. 2013. “A Fröhlich Effect and Representational Gravity in Memory for Auditory Pitch.” *Journal of Experimental Psychology.* 39 (4): 1153–64. doi:10.1037/a0031103.

Huron, David. 2001. “Tone and Voice: A Derivation of the Rules of Voice-Leading from Perceptual Principles.” *Music Perception* 19 (1): 1–64.

———. 2006. *Sweet Anticipation: Music and the Psychology of Expectation*. Cambridge, MA: MIT Press.

Knochenklang, Ensemble Paläolithisches. 2006. *Bone tone sounds from the Stone Age [Knochenklang Klänge aus der Steinzeit]*. Vol. 1. Vienna, Austria: Verlag der Österreichischen Akademie der Wissenschaften.

Krumhansl, Carol L. 1990. *Cognitive Foundations of Musical Pitch*. New York: Oxford University Press. doi:10.1121/1.404005.

Kunej, Drago, and Ivan Turk. 2000. “New Perspectives on the Beginnings of Music: Archaeological and Musicological Analysis of a Middle Paleolithic Bone ‘Flute.’” In *The Origins of Music*, edited by Nils Lennart Wallin, Björn Merker, and Steven Brown, 235–68. Cambridge MA: MIT Press.

Kushnaryov, Christofor. 1958. *Matters of History and Theory of Armenian Monodic Music [Вопросы Истории И Теории Армянской Монодической Музыки]*. Edited by Robert Atayan. Art Instit. Moscow: Gos Muz Izdat [Гос. муз. изд-во].

Kvitka, Kliment V. 1971. *Selected Works [Избранные Труды]*. Edited by Goshovskii V. L. Vol. 1. Moscow: Sovetskii Kompozitor [Сов. композитор].

Léothaud, Gilles, Frédéric Voisin, Alexandra Lamont, and Simha Arom. 1997. “Experimental Ethnomusicology: An Interactive Approach to the Study of Musical Scales.” In *Perception and Cognition of Music*, edited by Irène Deliège and John A. Sloboda, 3–30. Hove, UK: Psychology Press.

Marmel, Frédéric, and Barbara Tillmann. 2009. “Tonal Priming beyond Tonics.” *Music Perception* 26 (3): 211–21. doi:10.1525/mp.2009.26.3.211.

Mazel, Lev. 1952. *On Melody [О Мелодии]*. Moscow: Gos Muz Izdat [Гос. музыкальное изд-во].

———. 1982. “On Certain Aspects of Asafyev’s Concept [О Некоторых Сторонах Концепции Б.В. Асафьева].” In *Essays on Theory and Analysis of Music [Статьи По Теории И Анализу Музыки]*, 277–307. Moscow: Sovetskii Kompozitor [Советский композитор].

Mazo, Carlos, Marta Alcolea Gracia, and Carlos García Benito. 2014. “Flute of Isturitz? Experimental Reproduction and Archaeomusical Analysis.” In *XVII International Congress of Prehistoric and Protohistoric Sciences*. Burgos, Spain. doi:10.13140/2.1.2586.0484.

McGroarty, Patrick. 2009. “Prehistoric Flute in Germany Is Oldest Known.” *Boston Globe*, June 24. http://www.boston.com/news/world/europe/articles/2009/06/24/prehistoric_flute_in_germany_is_oldest_known/.

Münzel, Susanne, Friedrich Seeberger, and Wulf Hein. 2002. “The Geißenklösterle Flute: Discovery, Experiments, Reconstruction.” In *Archäologie Früher Klangerzeugung Und Tonordnung; Musikarchäologie in Der Ägäis Und Anatolien*, 10:107–18. Rahden, Germany: M. Leidorf.

Niemi, Jarkko. 1999. “The Genres of the Nenets Songs.” *Asian Music* 30 (1): 77–132. doi:10.2307/834981.

Pratt, R.L., and J.M. Bowsher. 1978. “The Subjective Assessment of Trombone Quality.” *Journal of Sound and Vibration* 57 (3): 425–35. doi:10.1016/0022-460X(78)90321-8.

Protopopov, Sergei. 1930. *Elements of Construction of Musical Speech [Элементы Строения Музыкальной Речи]*. Edited by Boleslav Yavorskii. Vol. 2. Moscow: State Edition, Musical Sector [Госуд. Изд-во Музык. Сектор].

Rasch, Rudolf, and Reinier Plomp. 1999. “The Perception of Musical Tones.” In *The Psychology of Music*, edited by Diana Deutsch, 2nd ed., 2:89–112. San Diego, CA: Academic Press.

Sachs, Curt. 1962. *The Wellsprings of Music*. Edited by Jaap Kunst. The Hague, Netherlands: Martinus Nijhoff.

Smith, Nicholas, and Mark Schmuckler. 2004. “The Perception of Tonal Structure through the Differentiation and Organization of Pitches.” *Journal of Experimental Psychology. Human Perception and Performance* 30 (2): 268–86. doi:10.1037/0096-1523.30.2.268.

Tull, James Robert, and Boris Asafyev. 2000. *B.V. Asafʹev’s Musical Form as a Process: Translation and Commentary*. Translated by James Robert Tull. Photocopy: Vol. 3 volumes. Ann Arbor, Mich.: University Microfilms International [Publisher]. doi:610363518.

Tuniz, C., F. Bernardini, Ivan Turk, L. Dimkaroski, L. Mancini, and D. Dreossi. 2012. “Did Neanderthals Play Music? X-Ray Computed Micro-Tomography of the Divje Babe ‘Flute.’” *Archaeometry* 54 (3): 581–90. doi:10.1111/j.1475-4754.2011.00630.x.

Turk, Ivan, J. Dirjec, G. Bastiani, M. Pflaum, T. Lauko, F. Cimerman, F. Kosel, J. Grum, and P. Cevc. 2001. “New Analyses of the ‘Flute’ from Divje Babe I (Slovenia) [Nove Analize ‘Piscali’ Iz Divjih Bab I (Slovenija)].” *Arheoloski Vestnik* 52: 25–79.

Wakin, Daniel J. 2009. “Pondering Prehistoric Melodies.” *New York Times*, June 28.

Zarate, Jean Mary, Caroline R. Ritson, and David Poeppel. 2012. “Pitch-Interval Discrimination and Musical Expertise: Is the Semitone a Perceptual Boundary?” *Journal of the Acoustical Society of America* 132 (2): 984–93.

Zemtsovsky, Izaly. 1998. “The Melodic System of Pentatonism (a Sketch about the Mongolian Version).” In *Ethnologische, Historische Und Systematische Musikwissenschaft: Oskár Elschek Zum 65. Geburtstag*, edited by Franz Födermayr and Ladislav Burlas, 193–95. Bratislava: ASCO art & science.

Zhang, Juzhong, Garman Harbottle, Changsui Wang, and Z Kong. 1999. “Oldest Playable Musical Instruments Found at Jiahu Early Neolithic Site in China.” *Nature* 401 (September): 366–68. doi:10.1038/43865.

Zhang, Juzhong, Xinghua Xiao, and Yun Kuen Lee. 2004. “The Early Development of Music. Analysis of the Jiahu Bone Flutes.” *Antiquity* 302 (78, April): 769–78.

1. I use the word “pipe,” since there is not enough archeological evidence to conclude if the earliest instruments constitute flutes or clarinets. [↑](#footnote-ref-1)
2. Not all archeologists accept that this fragment was intentionally man-made. Some explain the origin of the hole by a carnivore's bite (D'Errico et al. 1998) – which is unlikely, because such bites produce cracks rather than clean cuts (Atema 2014). Ivan Turk (2001) experimentally tested the animal bite hypothesis by conducting an extensive series of biting tests using imprints of the bear, wolf, and hyena dentition - and rarely succeeded in making one hole without cracking and splitting the bone. [↑](#footnote-ref-2)
3. The principal cause for this greater fluidity of intonation in comparison with other Paleolithic instruments must be the significantly greater diameter of Divje Babe’s pipe. Wider sounding body seems to be the design feature that distinguishes this Neanderthal artifact from later pipes that were discovered in Germany. [↑](#footnote-ref-3)
4. C-D-F should be taken only as the approximation of tuning, expressed in Western letters only for convenience of readers not fluent in conversion of cents into intervals. [↑](#footnote-ref-4)
5. This term needs some explanation. Modern music theory reduces diatonicity to compliance to the circle of 5^ths^ – hence, holding that both, hemitonic and anhemitonic scales might be “diatonic” once their PS tones can be distributed by the circle of 5^ths^. However, this definition is a recent historic development. Originally, the term “diatonic” opposed the notion of anhemitonic music, favoring scales without “gaps”, and scales with more evenly distributed contrasting intervals of major and minor 2^nds^. [↑](#footnote-ref-5)
6. Addition and subtraction unfortunately work differently in Western music theory than in math. [↑](#footnote-ref-6)
7. Even if a pipe player managed to invent a tonally advanced pipe call, the communication of it would have been problematic until all the members of the tribe would jointly establish a new convention. All samples of primitive art are syncretic. Every a single member of the tribe participates in the production and consumption of his tribe's music. That would necessarily require every tribesman to be able to play the melodic interval under question on the pipe - or at least, to sing it along with the pipe player, in close pitch. Only then this interval could become a musical idiom for that group of music users. The conventional musical intonations are always collective - they are the average of the skills of all the participants. Even if there were a caveman Paganini, his innovations would have necessarily been lost. [↑](#footnote-ref-7)
8. Of course, in order to establish the exact size of an interval in a tuning set of a bone pipe, a sufficient sample size is needed, where the frequency measurements of tones produced by the same fingering should be averaged between all the occurrences of those tones. Musical performance is highly volatile, and slight fluctuations in breathing or position of the pipe in relation to the mouth are capable of causing changes about 20-40 cents in frequency of a tone. My measurements of pipe samples did not follow the unified routine of averaging tuning values between different samples, and therefore should be taken as a very rough demonstration of Beliayev-based method of estimation of modal design of an instrument and interpretation of the produced results. [↑](#footnote-ref-8)
9. Clarinet in Bb is used for keys with sharp key signatures, and Clarinet in A - for flat key signatures. [↑](#footnote-ref-9)
10. There is a more recent finer replica built by Wulf Hein, which has a slightly different tuning of the higher pitches, but unfortunately he did not have a recording of that model, and Suzanne Münzel, who is in charge of that replica at University of Tübingen, could not provide information on its tuning. [↑](#footnote-ref-10)
11. Published by the Nature Magazine (<http://www.nature.com/news/2004/041213/multimedia/041213-14-m1.html>) [↑](#footnote-ref-11)
12. All pitches were extracted from a clip kindly provided by Carlos García Benito. [↑](#footnote-ref-12)
13. Its replica, prepared by Wulf Hein, was used for the production of a CD (Knochenklang 2006). [↑](#footnote-ref-13)
14. Ever since many museums went into the business of selling replicas of old instruments from their collections, not all replicas are found to faithfully reproduce the original instruments – as museums often manufacture and distribute "fantasy" instruments under the guise of “authentic”, modifying them for the purpose of a "better" sound. [↑](#footnote-ref-14)
15. However, except Isturitz 83888(a) pipe, the oldest instruments contain quite similar distances between the marginal holes and the pipe end – therefore, suggesting little impact of “inversion” of the flute on the IS. [↑](#footnote-ref-15)
16. This article contains serious inaccuracies. The tuning figures for the M341:1 pipe in the graph “Interval (cents)” are all wrong: the correct figures, inferred from the upper graph (Pitch) should be 215; 325; 182; 493; 483. The same mistake applies to the M341:2 pipe. The correct figures should be: 303; 197; 304; 201; 195; 505. The M:282:20 pipe’s tuning should read as: 350; 178; 152; 200; 200; 100; 400. The correct values for M:282:21 pipe are: 364; 104; 277; 156; 318; 35; 317 – and for the M253:4 pipe: 385; 115; 115; 18; 176; 46; 377; 200. The authors are not aware that the distance between F and A constitutes 4 semitones: in all their tables they mark it as 300 cents (3 semitones). Mistakes in estimation of the intervallic values between the tones of the holes lead to mislabeling of the notes. Thus, they pronounce the M282:21 pipe’s lowest holes tuned to A6-A6, whereas they are separated by 104 cents (over a semitone apart). Clearly, both tones should be qualified as A-Bb. [↑](#footnote-ref-16)
17. I do not have complete confidence in the accuracy of the acoustic measurements of tones reported in the 2004 article. The audio recording of the M282:20 pipe published by the Nature Magazine (Zhang et al. 1999) features a PS of A5-B5-D6-E6-F#6-A6 – which should be difficult for a performer to maintain, if to consider the tuning for the M282:20 reported in the 2004 article true (since the player would have to consistently use his lips to alter the tuning of 3 holes). Besides, the 1999 article by the same principal author, Juzhong Zhang, as the 2004 article reported different interval values between the holes of the M282:20 pipe, as compared to the 2004 article. It is hard to conclude, whether the 1999 publication was based on the measurements of an imprecise replica, while the 2004 one – on the original, or whether a mistake was made in naming the pipe. I would assume that the 2004 article is more reliable, since it was dedicated to evaluation of the sonic properties of the original 5 pipes (rather than the news release about a single pipe in a popular magazine – what the 1999 publication appears to be), and came out 5 years later. [↑](#footnote-ref-17)
18. If such a refined intonation is anachronic, then we are left with two options: either the reported 35 cents is a mistake by the authors of the study, or the pipe makers made two versions of upper F6 – each for its own PS, where F would have to match some other tones in harmony, which required a difference in 35 cents. However, this last probability is small, since playing alternative PSs is much more comfortable on two dedicated instruments, and necessity to play modulations from one PS to another is highly unlikely to arise in a culture that has formulated the pentatonic mode just about 400 years ago. The authors of the study (Zhang, Xiao & Lee 2004) propose that the maker of the pipe drilled a hole, became unhappy with its sound, and drilled another hole to correct his initial mistake. This explanation raises the question, why did the maker then not simply discard the bad pipe and make another one? Both F5 and F6, and A5 and A6 are octave equivalent, suggesting that F and Bb were used as anchor points, since A and Bb appear to define a normal size leading tone. Had this been the case, then D6, F#6, and A6 could have been used together for another PS to compliment playing in the ensemble with another pipe. [↑](#footnote-ref-18)
