# Supplementary material for: Evolution of tonal organization in music mirrors symbolic representation of perceptual reality. Part-1: Prehistoric
Source: Front Psychol. 2015 Oct 16;6:1405. doi: 10.3389/fpsyg.2015.01405 (PMC4607869; doi:10.3389/fpsyg.2015.01405)
Supplement: Supplementary file 5 [file DataSheet1.ZIP › Appendix I.PDF]

Appendix I. Taxonomy of tonal organization of modal music

The following outline, illustrated with the analysis of a Yakut lullaby, might be helpful to those researchers who are willing to comparatively analyze different samples of modal music. I follow the scheme formulated by Tatyana Starostina (Starostina 1973), based on the theoretic distinctions proposed by Yuri Kholopov (Kholopov 1988, 115–145), with my own addition of 1h).

Overall, a mode is categorized in 5 aspects.

1) By intervallic type – that is, a kind of principle used to form a melodic contour. The intervallic structures can be one of the following types so far identified in known music:

a) **ekmelic** – where the mode features unfixed, variable, and/or non-periodic tones whose frequencies cannot be expressed in harmonious ratios, including speech-like tones, as well as those tones that vary significantly in pitch when the same melodic pattern is repeated [*this intervallic type is used in pre-modal, khasmatonal and ekmelic stages of tonal evolution*];

b) **anhemitonic** (diatonic) – where IS is confined to a set of intervals no smaller than a *whole* step, where all tones of the mode can be positioned in a circle of perfect 5^{ths}, and whose gamut does not contain more than 2 steps in a row [*corresponds to the pentatonic model, described in my paper, and the pentatonic polymodal system*];

c) **hemitonic** (diatonic) – where IS is confined to a set of intervals no smaller than a *half*-step, where all tones of the mode can be positioned in a circle of perfect 5^{ths} (it might be incomplete), and whose gamut does not contain 2 semitones in a row [*corresponds to the heptatonic model and the diatonic polymodal system*];

d) **hemiolic** (non-diatonic, related to "chromatic" genus of Ancient Greek music) – "gapped" tetrachord-based modes that feature tones separated by a *step-and-a-half*, where all tones in the mode *cannot* be positioned in a circle of perfect 5^{ths}, and the gamut can contain 2 semitones in a row. Gapped tones represent discrete degrees of the mode rather than alteration of degrees in the heptatonic mode; permanence of a hemiolic interval throughout a music work determines whether or not the mode is hemiolic [*corresponds to the music systems that grew out of Ancient Greek chromatic polymodal system, called "Mediterranean tonality" in my paper*];

e) **mixodiatonic** (quasi-diatonic) – based on mixture of diatonic pentachords, tetrachords, trichords and dichords, where all tones in the mode *cannot* be positioned in a circle of perfect 5^{ths} (i.e. melodic minor or Podhalean mode); or include more than 7 tones (Obykhodnyi mode), or feature modal mutability [*incorporates oligotonal, mesotonal, multitonal models and non-octave supermodes*];

f) **chromatic** – where IS features numerous tones separated by a half-step, on degrees other than those around the I and V, provided these semitonal tones are used consistently throughout the music [*corresponds to the 19th and 20th century Western music*];

g) **microchromatic** (related to "enharmonic" genus of Ancient Greek music) – where IS contains fixed tones that are separated by the intervals smaller than a semitone, provided they are used consistently throughout the music [*corresponds to some non-Western systems like maqam, raga, as well as new systems introduced in the 20th century Western music, i.e. Alois Hába and Ivan Wyschnegradsky*].

h) **mixochromatic** – based on mixture of diatonic and chromatic dichords, trichords, or tetrachords, where the chromatic components can comprise periodic/symmetric structures (octatonic scale) or feature gaps [*corresponds to the 19-20th century Western music, i.e. music by Alexander Scriabin and Charles Ives, as well as the advanced composite modes of the pentatonic and diatonic polymodal systems adopted in some folk cultures*].

2) By scaling – that is, the number of tones engaged in a PS, taken with their respective distances. This "scale" (rus. "masshtab" – scale that is used in maps) should not be confused with "scale" as a gamut. The difference is that gamut-scale rotates intervals in order to align all tones, whereas modal "scaling" considers tones in their absolute registral positions. Therefore, scaling reflects gaps (i.e. representing a tetrachord with the sub-4th as G-C-D-E-F rather than C-D-E-F-G), or/and marking non-octave equivalent tones (i.e. flattened high VII and sharpened low VII). Scaling is categorized as:

a) **oligotonal** – dichords, trichords, and tetrachords;
b) **mesotonal** – simple pentachord and more complex pentatonic and hexatonic modes;
c) **multitonal** – heptatonic, octatonic, non-octave, chromatic, and microchromatic modes.

3) **By ambitus** – that is, the position of stable tone/tones in the register, in relation to the rest of the tones of the gamut-scale – and the resulting major, minor, or diminished inclination of the mode (if its structure stresses the pentachord or "tonic" and mediant 3rds):

a) **authentic** – where the stable tone is located at the bottom of the scale, with no more than one tone beneath it;
b) **plagal** – that is, where the stable tone is closer to the middle of the scale, with two or more tones underneath it.

The difference between ambitus and scale is that ambitus takes into consideration stability, whereas scale does not. This difference is even more pronounced in polyphonic music, where each part might feature its own ambitus while sharing the same scale.

4) **By resolution system** – that is, the prevailing style of a melodic organization that is manifested in a way that melodic sentences (strophes) start, reach the climactic point and are finalized. Defining the resolution system involves:

a) identifying the principal stable tone (finalis or tonic);
b) identifying the alternative stable tone (repercussio or dominant);
c) finding the cadences by tracing the tonal vectors that usually descend from "dominant" to "tonic" - the word "cadence" itself comes from Latin "cadere" – to fall;
d) establishing the melodic formula that carries a specific function (initio or finalis).

5) **By thematic material** – that is, establishing melodic structures that prevail in the music work or its section (Kholopov 2005). Many types of modal music are based on repetition or variation of the same formula. More complex compositions can use numerous formulas, or contain contrast between formulaic and recitative style melody. Lyrics might prompt to define the thematic makeup, both, by syntax of the phrases and syllabification style (distribution of tones of the melody in relation to a sung syllable of text).

Each of the aspects of modal organization is associated with certain semantic values, determined by cultural conventions, but to some extent, prompted by psychoacoustic properties of an aspect.

Ekmelic intervallic type involves scalable intervals, which might be responsible for common animistic associations shared by its music users: volatility of an interval in a pitch contour and its susceptibility to melodic inertia are symbolic of beliefs that melodic formula represents the spirit of a person, ancestor, animal, or demon. The size of an interval depends on the spiritual state of the performer, so tonal organization is indeed animated by "spirit".

Anhemitonic intervallic type is characterized by the greatest harmonicity amongst all types, due to the absence of semitones, and constant counter-balancing of 2nds with 3rds in stepwise melodic motion. In contrast, hemiolic type bears much more tension due to the contrast in size between semitone next to the gapped interval. In Mediterranean and Central Asian music hemiolic modes are often associated with emotionally stirring dance or song. Chromatic type also usually bears affinity with more emotionally intense expression, often presenting the opposite pole to the anhemitonic type.

The rest of the intervallic types are less specific in their semantics. Hemitonic type occupies the interim position between the chromatic and anhemitonic types. Mixodiatonic type usually features more tension than the hemitonic diatonic type, and often contains more complex melodic morphology, including modal mutability. Quite common is association of microchromatic type with "shadings" in tension, and therefore certain finesse or sensuality.

Finally, mixochromatic type is often brought in Western art music by the need to present exotic method of tonal organization, not as intense as in the chromatic type, but different from conventional schemes. Composers like Liszt, Rimsky-Korsakov, Scriabin, Stravinsky, and Messiaen have used this type to construct modes for expression of strange, supernatural or overwhelming characters.

Scaling types also bear semantic connotations. Music written in oligotonal modes appears simple and straightforward, lacking in capacity to be developed and transformed. Mesotonal modes usually carry more of a balanced characterization, allowing sufficient diversity in expression while sounding clear and easy. Multitonal modes are used for expression of more contrasting or unsettled content.

Typology of ambitus is perhaps the most explicit in semantic denotations, as compared to other aspects. Major inclination is nearly universally accepted as expression of positive emotional state, while minor inclination - of negative. This categorization is not necessarily the ultimate one: even in Western classical music minor key often stands for expression of "personal" emotional state as opposed to "communal" feelings, associated with major (as reflected in the etymology of these two words).

The diminished inclination occurs in modes that mark the tritone as a pentachord base, i.e. Locrian mode (which is quite popular in Eastern European music, against the claims made in many music theory textbooks). Such modes are usually perceived as "darker" than minor.

The other two ambitus types, authentic and plagal, are also identifiable by ear, albeit to a lesser extent. Authentic mode can be recognized by cadence that features strong contrast in stability/instability, making finalization sound more pronounced. Plagal mode can be recognized by cadence that features vague contrast in stability/instability, making finalization sound smooth.

The resolution system manifests itself through the distribution of tension and relaxation within a music work. Stressing of "tonic" and its frequent use makes the music appear confident, relaxed, and/or strong or unified in its emotional expression. Stressing of "dominant" and its prevalence over tonic increases overall instability and dynamism in melodic progression, often generating belaboring or struggling impression.

Cadences are usually heard as "making a statement" in melodic expression. Lack of cadences contributes to confused, unsettled or conflicting characterization.

These are the generalizations about the semantic attributes of aspects of modal organization, shared in the ethnomusicological literature of Russia and other countries of former USSR. Their accuracy remains to be tested, but the wide practice of research, transcriptions, and concert performance of folk music by professional musicians indicates that at least in relation to the music that is native to researchers, subjects of their study, as well as the audience, the above-mentioned framework of modal analysis indeed captures the essence of tonal organization.

Below, is an example of modal analysis according to this scheme.

Press the **Play** button.

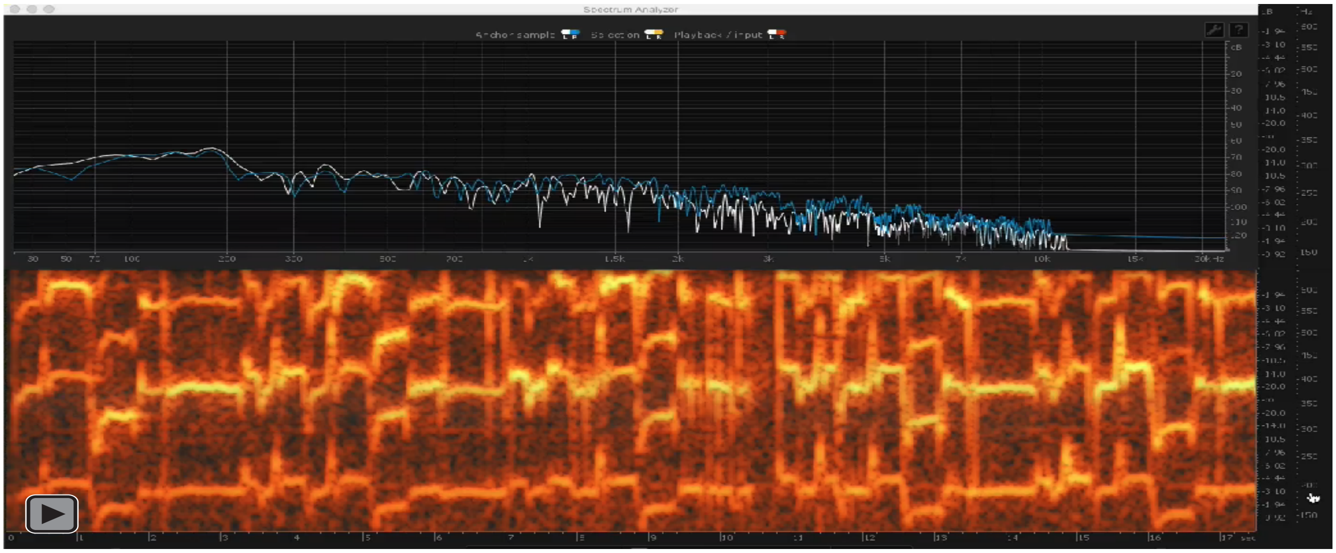

Yakut lullaby Bisik Yryata [Биник ырыата], performed by Ustin Nokhsorov at the Folkloric Research Lab of Moscow Tchaikovsky Conservatory (1946). This example and the figure below are taken from "Problems in genesis of mode" by Eduard Alekseyev (Alekseyev 1976, 49), along with its transcription, used here with the kind permission of the author.

The spectrogram shows the 2-octave range (vertical axis) and a 17-second sample that contains the opening melodic formula and 4 of its repetitions. The pitch contour strictly follows the same wave-like pattern, with elongation of the crest by means of the auxiliary tone. However, the presence of *kylysakh* - the spikes of extremely short tones that anticipate the regular degrees, sounding like dazzling sparks over the melody – complicates the contour. When comparing the repetitions of the formula, we see that the kylysakh pitches stay relatively fixed, and use wider intervals, as compared to the intervals between the principal degrees. By looking at the two formants, one can see how much information is transmitted by spectral recoloring of the tones of the melodic formula. Some tones emphasize the 1st formant, others – the 2nd – the louder audio components have brighter color. In fact, most dynamic activity occurs not in the fundamental tones, but in the formants.

The notation below (the first 5 bars) corresponds to the audio above:

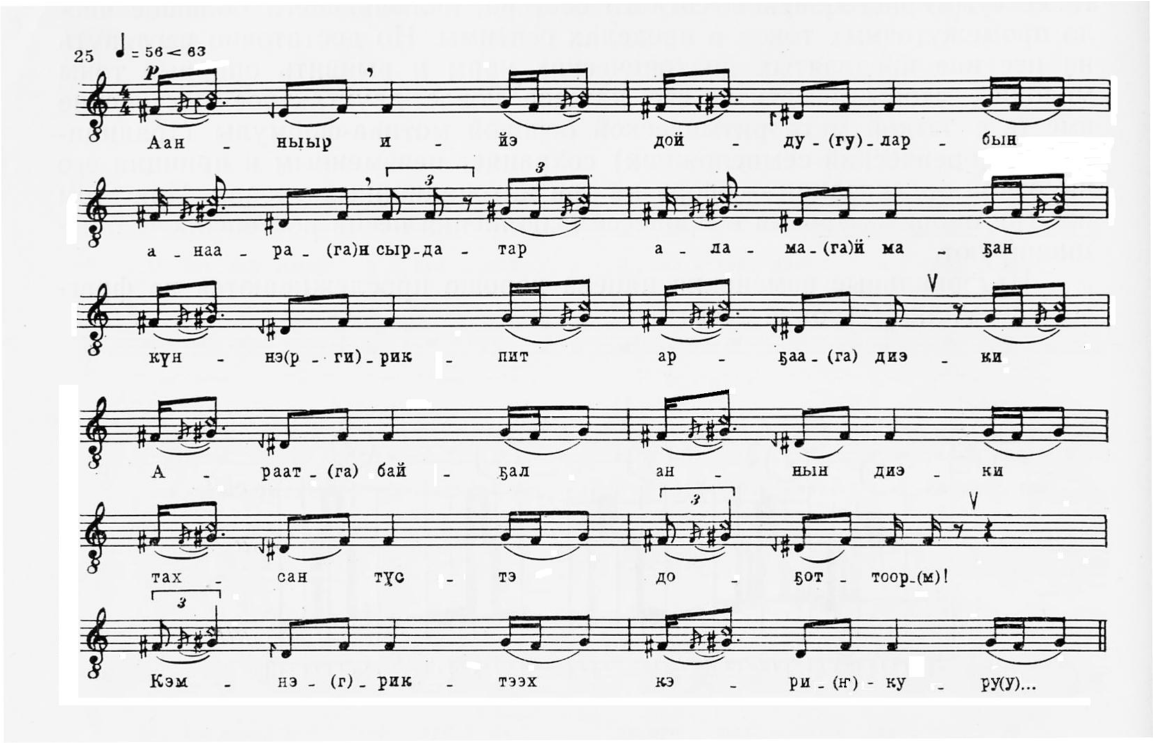

This music is based on the anhemitonic IS, with influence of ekmelic and khasmatonal types. The khasmatonal influence is evident from the presence of kylysakh, reflected by the grace notes. When going from one note of the melody to the next, before reaching that next note, a grace note quickly leaps much higher than this targeted next note of the melody, dynamically and timbrally off-setting the smooth contour of the melodic line.

The ekmelic influence is evident from the changeable tuning of the pitches of the lowest and the highest degrees. At the start of the music, the upper degree is 204.5 Hz (G3-26 cents), and the lower degree is 162.8 Hz (E3-21). In the 4th bar the upper tone sharpens to 208.2 (G#+5), whereas the lower tone flattens to 159.2 (D#+39). In the 12th bar the upper tone reaches 215(A3-40), while the lower tone descends to 152(D#3-40). This centrifugal expansion of the ambitus, as the song progresses, totals 305 cents (minor 3rd) – very characteristic to the ekmelic organization. This expansion is shown on the drawing by Eduard Alekseyev that represents bars 1, 3 and 12 (used with his permission).

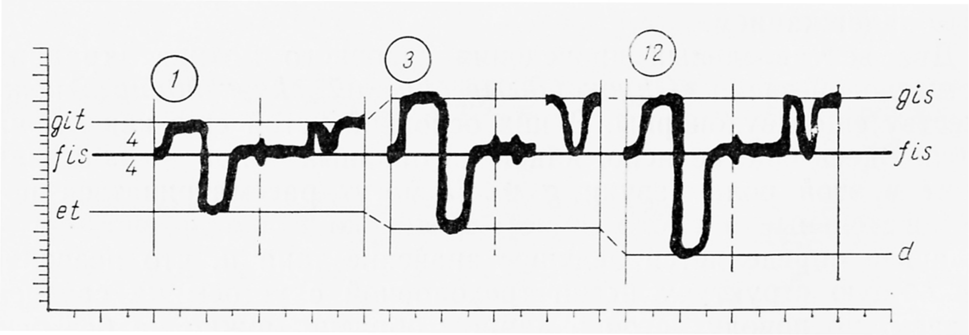

This lullaby is based on the oligotonal PS of 3 degrees. The lowest I degree is separated from the II by a scalable 3rd, and the III from the II – by a scalable 2nd. Hence, the distribution of gravity goes in the following order: II is stable, I is opposing stable, and III is supporting stable II degree. The extreme degrees (I and III) are separated by the scalable 4th.

The ambitus is authentic: both unstable degrees are significantly weaker than the stable one (authentic inclination is usually more intense than plagal). The tonicity of F# is emphasized mainly by the stability in its tuning – in contrast to shifting tuning of the marginal tones. Absence of an extra degree does not allow the melody to display either major, or minor inclination.

The melodic formula starts on the II degree and ends on the III degree. The I degree opposes the II degree, while the III degree compliments the II degree. This sets the I and the III degrees in "extreme" relation: opposite (I) versus supporting (III) functions. Such functionality marks the I degree as bearing more gravity than the III degree: the I degree is used to challenge the stable II degree, whereas the III degree merely serves as an auxiliary tone for the II degree.

The formula consists of 3 melodic intonations, each based on its dedicated degree. The opening intonation energetically bounces of the II degree, with the reverse puncture ("Lombardian") rhythm, further activated by the kylysakh in the ascending motion. The second intonation is similar to the first one in its pitch contour, but differs by leaning on the I degree - in a heavier fashion due to its even rhythm and absence of kylysakh. This intonation is also metrically longer: it adds an extra tone of greater rhythm value, repeating the same pitch, thereby stressing the II degree and giving it tonal priority. The last intonation mirrors the first one in its metric value, but emphasizes the auxiliary III degree. This intonation is the most active of the three: it contains 2 shortest notes followed by a kylysakh. The middle intonation clearly contrasts both marginal ones.

Consecutive repetitions of these 3 symmetric intonations produce the impression of a wheel-like rotation – which agrees with the lullaby genre application of this music.

So, to sum up, the modal organization of this lullaby can be qualified as oligotonal 3-degree authentic F# mode with unfixed I and III degrees.

Western ethnomusicologists usually follow a similar procedure in analysis of musical material collected in the fieldwork. The principal recent innovation is the usage of computer applications to track the changes in tuning of the degrees in question in a PS. Thus, Arom (Arom, Fernando & Marandola 2007) describes modal analysis as:

1) determination of the number of degrees;
2) observation of their change over time, from one position to another;
3) evaluation of the size of the intervals that separate various degrees;
4) definition of ambitus, stable tones, and grouping of tones in the melody.

What is missing here, as compared to the Russian methodology, is categorization of the intervallic type and identification of functions of the discovered intonations. However, Arom's method offers a substantial advantage by providing an opportunity to verify the analysis by generating computer music according to the hypothesized tonal organization, and playing it back to the original performers asking for their feedback (if they recognize this music as a legitimate representation of their practice).

It should be noted that works of classical music can also be analyzed according to this modal scheme, since tonality employs a particular form of modal organization (Kholopov 2005). This task involves greater complexity, since it is very common to have *tonal* functional organization of *vertical* harmony while *modal* organization of *melodic* harmony. Their relative share in tonal organization can vary considerably between:

1) modal melody just flavoring the functional harmonization (Borodin – the opening of the Symphony No.2) to
2) harmonization strictly confined to tones of the melodic PS (Symphonic poem "Caravan" (1946) by Soltan Hajibeyov).

A classical music composition can fit anywhere in between these two poles – in fact, a unique position between the two can probably be qualified as the earmark of tonal organization, a marker of an individual style that a composer is supposed to put forward, according to aesthetic requirements in Western music culture.

REFERENCES:

Alekseyev, Eduard. 1976. *Problems in Genesis of Mode [Проблемы Формирования Лада]*. Muzyka [Музыка].

Arom, Simha, Nathalie Fernando, and Fabrice Marandola. 2007. “An Innovative Method for the Study of African Musical Scales: Cognitive and Technical Aspects.” In *Proceedings of the 4th Sound and Music Computing Conference, Lefkada, Greece*, edited by Charalampos Spyridis, Anastasia Georgaki, Georgios Kouroupetroglou, and Christina Anagnostopoulou, 107–16. Athens, Greece: University of Athens.

Kholopov, Yurii. 1988. *Harmony: A Theoretic Course [Гармония: Теоретический Курс]*. Moscow: Muzyka [Музыка].

———. 2005. “Towards the Problem of Mode in Russian Theoretic Musicology [К Проблеме Лада В Русском Теоретическом Музыкознании].” In *Harmony: Problems of Science and Methodology [Гармония: Проблемы Науки И Методики]*, 2:135–57. Rostov-na-Donu: RGK [Ростовская государственная консерватория].

Starostina, Tatyana. 1973. “Modal Systematization of Russian Traditional Song [Ладовая Систематика Русской Народной Песни].” In *Harmony: Problems of Science and Methodology [Гармония: Проблемы Науки И Методики]*, 1:85–105. Moscow: Muzyka [Музыка].
