# Supplementary material for: Evolution of tonal organization in music mirrors symbolic representation of perceptual reality. Part-1: Prehistoric
Source: Front Psychol. 2015 Oct 16;6:1405. doi: 10.3389/fpsyg.2015.01405 (PMC4607869; doi:10.3389/fpsyg.2015.01405)
Supplement: Supplementary file 5 [file DataSheet1.ZIP › Appendix IV.DOCX]

## Appendix IV. Comparison of the principal stages of tonal organization:

|  | PS | PSC | OD | IS | ISC | GS in PS | Tension | M/A |
| --- | --- | --- | --- | --- | --- | --- | --- | --- |
| Pre-mode | Indefinite pitch | - | Timbre-frequency syncretism of the snapshot-like pitch contour | The entire compass works as a single interval | - | Reference to the central position in the vocal compass | Attraction of tones to start or end points in a pitch contour | - |
| Khasmatonal mode | Indefinite pitch | - | Timbral transformation of tones over the range of the vocal compass | 2-3 melodic registers, each marked by strong timbral contrast | Contrast of gradual vs. abrupt timbral changes, supported by corresponding changes in pitch | Reference to the central position in a register of the vocal compass | Attraction of tones to the same register, and repulsion from another register | - |
| Ekmelic mode | Indefinite pitch | - | Frequency transformation over a specific range (2^nd^ – 4^th^), defined by shifts in melodic contour | Fixed unison & 2^nd^ contrast scalable 3^rd^ & 4^th^, all distinguished by their order in registral distance from an anchor | Adherence of each interval to a particular degree of the mode and influence of that degree’s melodic functionality | Centrifugal model, with strong melodic inertia & weak centrality of anchors | Attraction in complimenting vs. repulsion in opposing or extreme melodic relations of tones | - |
| Oligotonal mode | 1-2 definite pitches and 1-3 pitches variable in tuning within the range of a 3^rd^ | Formative role of zigzags and skips over a tone in the melodic contour + tuning in collective performance | Categorization of degrees by width of their pitch zones, defined by their registral order | Fixed unison, 2^nd^, 3^rd^ & 4^th^ in melodic & vertical versions + vertical octave. Equivalence of 2^nd^ (step equivalence) | Melodic consonance or dissonance of an interval & its position in the ambitus. Genesis of vertical harmony & harmonized texture | Balanced centrality of anchors, weak melodic inertia, bareness of ambitus | Attraction of tones variable in tuning to tones permanent in tuning, which are more frequently used | Transposition of PS (or its subset) by interval |
| Mesotonal mode | 1-3 definite pitches and 2-4 pitches variable in tuning within the range of a 2^nd^ | Contrast of fixed odd & flexible even degrees in pitch, with ^1^/_2_, 1, and 1 ^1^/_2_ increments between them | Categorization of degrees by their registral order & smaller/larger intervals | Added fixed tritone, 5^th^ & 6^th^ + their vertical versions. Equivalence of 3^rd^ (even/odd) | Gradations in melodic consonance/dissonance of a melodic interval + even or odd registral order, equalized by 3^rd^ | Balanced centrality of even or odd degrees, 2-level hierarchy, mutable I-III (II-IV) anchors | Permanent stability or instability of the degrees, induction of resolution & tonicity | Transposition of motifs by degree within a mode |
| Multitonal mode | 3 definite pitches and  4 (or more) pitches flexible in tuning (within a semitone), partial octave equivalence, on the tetrachord base | Contrast between triads on odd & even degrees, odd one becoming central and octave equivalent, while even one – not. Limited octave equivalent tuning | Categorization of degrees by odd/even triads. Expansion of ambitus. Multiple inflections of unstable degrees | Added fixed 7^th^ & octave + compound vertical intervals. Equivalence of 4^th^ & limited octave equivalence afford inversion | Categorization of major/minor intervals, uniformity of intervals across different degrees, discovery of IC & ISC. Codification of vertical harmony | Strong centripetal gravity, with  4-level hierarchy, with common alternation of 2 “tonics” VI-I | Contraction of spaces between the degrees: induction of the leading tone, fine gradations in attraction. Rise of natural “chromatic” modes | Transposition of PS by interval and by degree: from I to low VI degree within a mode |
| Pentatonic mode | 5 fixed diatonic pitch classes, with full octave equivalence, on the trichord base, limited major/minor inclinations | Asymmetry in distribution of three 1 tone- and two 1 ^1^/_2_ tone-apart tones within an octave. Formative role of conjunct vs. disjunct trichords | Categorization of degrees by their position in a trichord in relation to the 5^th^ & 4^th^ between the degrees, octave functionality | Exclusion of minor 2^nd^, major 7^th^ & tritone. Equivalent 5^th^ & octave. Prevalent melodic consonance | Definition of intervals by means of octave & 4^th^/5^th^. Tendency to equalize harmonic consonance & dissonance | Very much diffused gravity, 2-level hierarchy for stable degrees only | Weak attraction, with segmentation in “terraces,” with avoidance of skips. Plagal & authentic typology | Natural “enharmonic” modulation: rotation of trichords. Auxiliary alterations |
| Heptatonic mode | 7 fixed diatonic pitches classes, with full octave equivalence, on the pentachord base, uniform major/minor inclinations | Asymmetry in distribution of five whole tone- and two semitone-apart tones within an octave. Formative role of conjunct vs. disjunct tetrachords | Categorization of degrees by their position within the pentachord base, octave equivalent functionality of all degrees | Opposition of perfect & major/minor intervals, induction of chords. Full octave equivalence. Common melodic dissonance | Definition of intervals through octave & 4^th^/5^th^. Increase in contrast of harmonic & melodic consonance vs. dissonance. Formative role of inversion | Centripetal hierarchy,  2-level for stable & 3-level for unstable degrees, with common mutability I-IV | Uniformity of all degrees in their attraction/gravity values over an octave. Plagal & authentic opposition, minor is overall less stable than major | Natural “enharmonic” modulation: rotation of tetrachords. Auxiliary alterations |
|  | PS | PSC | OD | IS | ISC | GS in PS | Tension | M/A |
| Diatonic polymodal system | 2-6 fixed sets with shared 7 fixed pitch classes, on the pentachord base. Major, minor or diminished inclinations | Each mode’s idiosyncrasy in distribution of tone & semitone within the joint pentachord/tetrachord base, with full octave equivalence | The degrees are inferred from the circle of 5^th^ & octave transposition. Tetrachord marks stability as an inversion of the pentachord | Fixed gradations in harmonicity between ISs of sister-modes. Semantic specialty of each mode (genre), fixedness in its fine tuning | Formative role of the diatonic tritone, rotation of intervals in their stability values across sister-modes, strict inversion rules & genesis of codified music theory | Centripetal, uniform 2-level hierarchy of stable degrees across all modes, with formative I-V functionality | Functional equalization of all degrees between all sister-modes, without pronounced hierarchic relations between unstable degrees. Possible alteration | “Chromatic” modulation: within a mode through chromatic alteration that emulates the leading tone |
| Non-octave supermode | 8-12 (<18) fixed pitches with 1-4 false relations, on the tri-/tetra-/pentachord base. Major, minor or diminished inclinations | Contrast of tone & semitone within the subset, reproduced throughout the ambitus by means of the chain principle | The degrees are inferred from the circle of 4^th^ or 5^th^ through the trichord, tetrachord or pentachord chains | Natural intervals between the adjacent subsets vs. augmented or diminished ones between the marginal subsets | Equivalence of 4^th^ in all subsets or subset inequivalence (contrast) across the ambitus. Avoidance of melodic dissonance. No inversion | Centripetal or centrifugal gravity, without hierarchy, diffused between each subset’s anchors | Weak attraction, with tension defined by false relations, usually increasing in the top subsets & decreasing in the bottom subsets | Abstaining from modulation & alteration. “Shimmering” inflections by false relations |
| Chromatic polymodal system | 16 pitch classes with limited octave equivalence, on the tetrachord base.  Great multitude of inclinations | Contrast between the chromatic clustering (pyknos) and the hemiolic gapping at different sides of a stable tone. Fine gradations in chromatic shading | Contrast between immutable & mutable degrees within and between modal subsets. Diatonic framework of immutable degrees | Wide use of micro-intervals, more so in the mid-ambitus. Ample IS, based on harmonic 4^th^, 5^th^ & octave. Avoidance of melodic dissonance | Formative role of alteration and chromaticism (including microtonal shading). Enormously rich choice of ICs, determined by melodic resolution of the adjacent tones | Centripetal gravity with 2-level hierarchy, diffused between each subset’s anchors, with occasional mutability I-IV | Strong attraction between the pyknos tones vs. weak - in gaps, increasing at the center of the ambitus. 6 subset types of gravitational distribution | Transposition of PSC by degree and by interval. Rich assortment of modulations & alterations |
| Tonality (tonal key system) | 17 pitch classes with full octave equivalence, based on inversion of penta-/tetrachord. Major & minor inclinations are implemented as keys with multiple modes | Codex of complex correlated rules of coordination and subordination relations between the members of a major or minor PSC, determined in reference to “tonic” | Hierarchic division of octave into I-V & V-I subsets; then subdivision of I-III & III-V; then I-II, II-III, III-IV, IV-V, V-VI, VI-VII & VII-I; and then chromatic subdivision | Octave equivalence of vertical intervals & chords, generally “implied” in melody as well. Hierarchy of natural & chromatic ICs for harmonic & melodic intervals | Major/minor ISCs include their natural, harmonic & melodic inflections. Augmented & diminished intervals are used to define gravity & tension. Microtonal shading is fixed into PCs (D#≠ Eb) | “Heliocentric” 5-tier system: balanced centripetal gravity, with satellite centripetal GS in all incorporated subsets | Strong mono-gravity: attraction of all tones to a “solar” tone, despite melodic distance, with hierarchy of “planetary” attractions; multiple options for any tone to go to | System of key relationships, transposable by interval. Applied “dominants.” Chromatic enharmonic modulations |

**PS** = Pitch Set: a set of tones used to make a particular melody.

**PSC** = Pitch Set Categorization: the criteria for the frequency discrimination between the degrees of a mode.

**OD** = *Organization of Degrees*: the method of ordering the degrees within the mode.

**IS** = *Interval Set*: the intervallic distance between neighboring tones in the melody.

**ISC** = *Interval Set* Categorization: the criteria for discrimination between the intervallic values of the relations
 between the neighboring tones in the melody – according to the PSC.

**GS in PS** = *Gravitational Scheme in a Pitch Set*: prevailing method of uniting the tones within the PS.

**Tension**: the method employed for marking stability and instability of the tones.

**M/A** = *Modulation/Alteration*: handling of transitions from one mode to another, and of transposition of
 characteristic intonations within a particular mode.

This table presents the summary of the main features of 12 principal types of tonal organization, including Western
classical tonality. It highlights the most important changes in the way how each of the schemes of tonal organization
generates tonal unity within a mode.

“Pre-mode” to “Heptatonic mode” are covered in Part-1 of this paper.

“Non-octave supermode” to “Tonality” are covered in Part-2 of this paper.
